# Patient and public involvement in developing and validating an instrument for assessing the scaling potential of innovations in health and social services: A consensus study

**DOI:** 10.1371/journal.pone.0336245

**Published:** 2025-11-26

**Authors:** Roberta de Carvalho Corôa, Ali Ben Charif, Claude Bernard Uwizeye, Florence Lizotte, Amédé Gogovor, Robert K.D. McLean, Andrew Milat, Léonel Philibert, Louisa Blair, Kathy Kastner, Jean-Sébastien Renaud, France Légaré

**Affiliations:** 1 Department of Family Medicine and Emergency Medicine, Université Laval, Quebec, Quebec, Canada; 2 VITAM – Centre de recherche en santé durable, Université Laval, Quebec, Quebec, Canada; 3 CubecXpert, Québec, Canada; 4 Unité de Soutien au Système de Santé Apprenant Québec, Quebec, Quebec, Canada; 5 International Development Research Centre, Ottawa, Ontario, Canada; 6 Faculty of Medicine and Health Sciences, Stellenbosch University, South Africa; 7 School of Public Health, University of Sydney, New South Wales, Australia; 8 Université de l’Ontario français, Toronto, Ontario, Canada; 9 Patient Partner, BestEndings, Toronto, Ontario, Canada; Universiti Sains Malaysia - Kampus Kesihatan, MALAYSIA

## Abstract

**Background:**

Before proven health innovations are scaled, an assessment of their scaling potential can save resources and assure quality at scale. Involving the beneficiaries of scaling is necessary for it to be effective and relevant. We aimed to develop, with patient and public involvement (PPI), an instrument for assessing the scalability of innovations in health and social services and to establish content validity.

**Methods:**

We conducted a multiphase study based on the Integrated Knowledge Translation approach and the Montreal Model for PPI. A steering committee provided feedback throughout the project. Informed by a systematic review, the research team and steering committee selected promising items for inclusion in the instrument. In a two-round online Delphi process, patients and public representatives and other expert panellists reached consensus on the relevance, clarity and necessity of each item. Finally, with a patient partner and two scaling teams we developed the instrument and a manual.

**Results:**

The steering committee consisted of a patient partner, an expert in health measures and two policymakers who were experts in scaling. Based on the systematic review, we retained 43 items covering 12 domains. Two new items related to PPI and sex- and gender-sensitive scaling were validated by the committee. A 24-member Delphi panel assessed the resulting 45 items for content validity. Patients and public representatives constituted 29.1% of the panel and researchers 25%. Fourteen items were excluded for not reaching content validity thresholds. The final selection included three items added by panellists (consideration of national and local legislation, disadvantages of not scaling, and equity). Despite a low score, an item on sex and gender was retained as being essential for redressing consequences of inequities in health research.

**Conclusion:**

The final tool, the Innovation Scalability Self-administered Questionnaire (ISSaQ 4.0), includes 37 items across 12 domains and is available in French and English.

## 1. Introduction

Health and social service “innovations” can include interventions, products, technologies, programs, models, frameworks, or policies that are new or perceived as new by target populations [1]. The scaling of innovations in health and social services refers to a systematic, evidence-informed, and ethical process to increase the intended impacts of innovations that have proven effective [2]. The goal of scaling is to expand care to more people, to enhance the quality of care, and to promote more equitable care, i.e., care that includes everyone [2,3].

However, many innovations remain confined to local and pilot contexts [4–6]. Failure to scale in health and social services occurs partly due to a lack of understanding of how to enable effective scaling, and partly to the absence of evidence-based reflection on the potential of an innovation to be successfully scaled [7–10]. Many scaling frameworks recommend assessing scalability, i.e., the scaling potential of an innovation, as the first step toward scaling it [3,9–11]. The scalability assessment of an innovation is the systematic process of determining its potential to be successfully scaled for greater beneficial impact, e.g., measuring to what extent it can be expanded while maintaining its effectiveness [5]. Scalability assessment instruments help scaling teams make informed decisions about which innovations are ready for scaling and what additional evidence or adaptations are needed [6,12].

To build effective scaling strategies, scaling teams must consider multiple factors, including the characteristics of the innovation, contextual factors in the new setting, and the needs and views of the populations targeted by scaling.[5,6,11,13]. To ensure the scaled innovation reaches far, it is important to include a broad range of potential beneficiaries of scaling, i.e., of patients and the public, in the process of designing the scaling strategies [2,3,11].

Patient and public involvement (PPI) in scaling health and social innovations can be defined as the partnership established by scaling teams (e.g., researchers, policymakers, health professionals) with patients, citizens, communities, and other civil society entities to support the scaling [2,14]. PPI in scaling not only fosters dignity and respect by integrating experiential knowledge but also ensures that scaling plans align with the values, needs, and sensitivities of the communities they aim to serve. A 2024 scoping review showed that PPI can occur through all phases of scaling, i.e., in planning, implementing, and evaluating, and that it can improve their quality and appropriateness [2,15]. Working alongside those who will be affected by the scaled innovation helps innovators make values-based decisions and understand in advance the full range of impacts it may generate [2]. Thus in the creation of scalability instruments, too, the questions and item suggestions of patient and public representatives, the direct beneficiaries of scaling efforts, provide an essential perspective on the real-world relevance and scalability of innovations [2–4,16]. Nevertheless, a 2022 systematic review that identified 21 instruments for assessing the scalability of innovations in health and social services found none which reported involving patients or the public or considered them as potential users of the instruments [5].

In this study, we aimed to develop, with PPI, an instrument to assess the scalability of innovations in health and social services, to establish its content validity, and to produce an accompanying manual.

## 2. Methods

### Study design and overview

In this multi-phase study, we followed best practices for developing and validating scales for health research in developing the Innovation Scalability Self-Administered Questionnaire (ISSaQ 4.0) [17–20].

**Fig 1:**
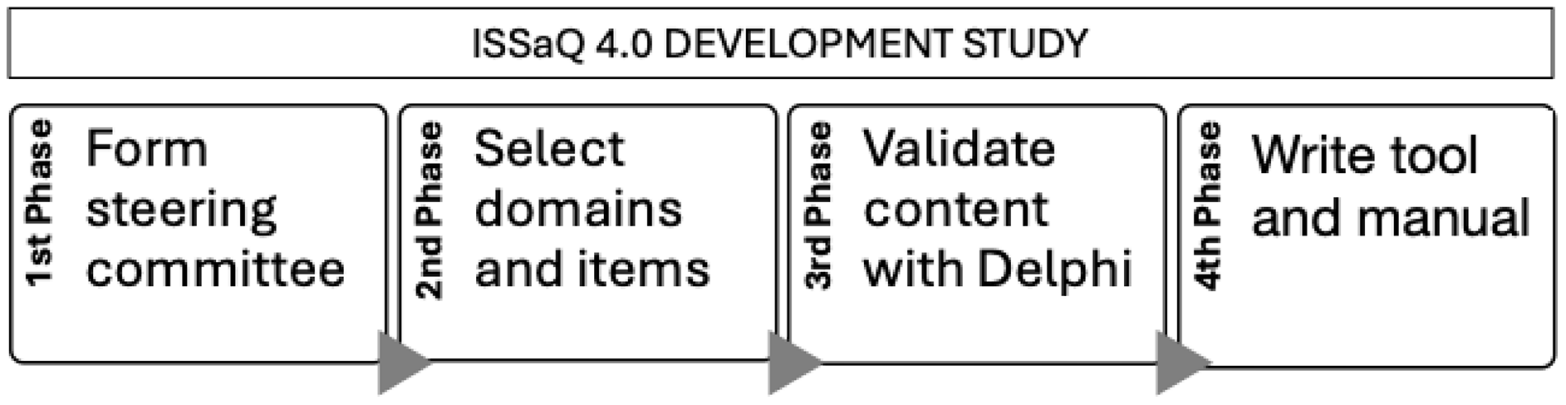
ISSaQ 4.0 development phases.

We collaborated with patient and public representatives alongside other scaling stakeholders throughout our research and incorporated PPI at each of the four phases of the study (Fig 1) [14,21]. We adopted the integrated Knowledge Translation (iKT) approach, which recommends involving knowledge users (e.g., policymakers, health professionals, patients and public representatives) as active participants in knowledge production from beginning to end in research projects [21]; and the Montreal Model, which invites researchers to partner with patients and public representatives and to integrate their experiential knowledge as complementary to scientific knowledge. We used the GRIPP2 checklist to report on PPI in our study (S1 File) [22].

We recruited a steering committee for sharing decisions on the project and giving feedback. With PPI, we reviewed and selected domains (broad thematic areas) and items (specific questions) for the scalability questionnaire based on a systematic review that we published in 2022 [5]. We then used a consensus approach to refine the final product and measure its content validity, i.e., its relevance, clarity, and necessity, using an online Delphi [23–25]. Finally, we wrote the ISSaQ 4.0 scalability instrument and a manual. We followed the Accurate COnsensus Reporting Document (ACCORD) checklist for reporting on the methods and results of the Delphi study (S2 File) [26].

The study protocol was not prospectively registered on any platform. Our systematic review on instruments for assessing the scalability of innovations in health and social services, which provided the set of items and domains for the Delphi, is published in open access form and includes the search strategy and search dates [5].

### Ethics approval

Ethical approval for this project was obtained from the Ethics Board of the Centre Intégré Universitaire de Santé et de Services Sociaux (CIUSSS) de la Capitale-Nationale (#2021−2016). All aspects of this project were designed and performed in accordance with the World Medical Association Declaration of Helsinki. Electronic and written informed consent was obtained from all the participants in the Delphi study.

### Phase 1: Steering committee composition

Our first phase was to form a steering committee of experts and potential users of a scalability assessment instrument. The committee was to give feedback on the research question and protocol and to validate the results of each phase of the project, including selection of promising domains and items.

#### Participants, eligibility criteria and recruitment.

We aimed to recruit at least one expert or user in each of the following categories: patients and public representatives, experts in scaling and health measures, and policymakers. Steering committee members were eligible if they were at least 18 years old, able to actively participate, read and understand English or French, and had knowledge of or interest in the scaling of innovations in health and social services. They were recruited using the snowball method via our network and were initially contacted by email.

#### Data collection and analysis.

Steering committee feedback on project decisions was collected through emails. For validation of project results, the research team sent project documents (e.g., tables and reports) and requested that committee members provide comments and suggestions (see Phase 2). Their comments and suggestions were synthesized into tables with frequencies and into text documents when appropriate. After integrating the feedback, the research team provided the committee members with reports detailing the changes made based on their feedback.

### Phase 2: Selection of promising domains and items

Domains are the concepts or themes that a measuring instrument (e.g., a scalability assessment instrument) is designed to address [18]. These should be described before selection of the instrument items [18,27]. Items are the questions or statements that address specific topics within each domain. Deductive methods are among the recommended practices for identifying items [18,28]. We had performed a literature review, published in 2022, to identify a preliminary list of domains and items for a scalability assessment instrument [5]. With the steering committee we proceeded to select relevant domains and items from this list.

#### Data collection and data analysis.

Our published systematic review on scalability instruments identified 320 scalability assessment items and 12 scalability domains which served as the starting point for the selection process [5]. Initially, we retained all the domains defined by the systematic review. For item selection, we first excluded any duplicates. Second, we merged similar items, i.e., items with the same meaning even if formulated differently. Third, we used 16 criteria designed to evaluate items for preference-based instruments (S3 File) [29]. These criteria included clarity of item phrasing, comprehensive coverage of contexts by the item, and the suitability of the item for use in assessment instruments (e.g., Does the item capture the intended concept? Is the item comprehensible, i.e., not ambiguous or poorly worded?). Two independent reviewers (RCC, LP) voted ‘yes’ or ‘no’ for each selection criterion and disagreements were resolved through consensus meetings with research team members. The wording of ambiguous or misleading items was revised. Items meeting all 16 criteria were then submitted to the steering committee (S4 File). We asked them to vote ‘yes’ or ‘no’ as to whether items should be included for content validity assessment in the Delphi, and to justify any exclusions. We also collected suggestions or comments. We synthesized the votes into tables with frequencies. Suggestions, comments and wording revisions were synthesized into reports. We recorded voting frequencies, and the research team made final decisions on item exclusions by consensus.

### Phase 3: The online Delphi

We conducted a Delphi study, a method whose usefulness is recognized for gathering input from individuals with diverse backgrounds and for its suitability in contexts involving ethical dilemmas, such as decisions about scaling in health and social services [16,23]. An online Delphi approach was chosen to minimize power imbalances through participant anonymity [30]. When it involves patients and the public, policymakers, and researchers as experts in their own right, the Delphi method fosters collective intelligence and serves as a valuable approach for meaningful user involvement [23].

We conducted a two-round online Delphi on the REDCap platform in both French and English [31]. We used the Content Validity Index (CVI) and the Content Validity Ratio (CVR) to establish consensus on item-level content validity, that is, on the relevance, clarity, and necessity of items [25]. CVI and CVR are the two scales most commonly used for content validation in health instruments [20,24,25]. The calculation of CVI and CVR is often achieved using Delphi studies [24].

#### Participants, eligibility criteria and recruitment.

Based on evidence on sampling trends and recommendations for Delphi studies, we aimed to ensure that a minimum of 16 panellists complete all the survey rounds by inviting at least 100 potential participants [32]. We aimed to include at least four patients or public representatives, two clinicians, two policymakers, two trainees, two scaling researchers, two first or last authors of scaling studies, and members of patient-oriented research support institutions (e.g., members of Support for People and Patient-Oriented Research and Trials in Canada) or of community-based primary health care (CBPHC) organizations [33]. Panellists had to 1) be aged at least 18 years old, 2) be able to provide informed consent, 3) be able to actively participate, read and understand English and/or French, and 4) have knowledge of or interest in the scaling of innovations in health and social services. They could not already be members of the steering committee.

We used three recruitment strategies. To recruit patients and public representatives, we asked the members of Quebec’s patient-oriented research support institution (Unité de Soutien SSA Québec) to send our invitation to their patients and public partners. The researcher leading the project contacted each person who had accepted the invitation to explain the project, clarify their role, and communicate her availability to support them during the online Delphi process. To recruit scaling researchers, we emailed authors of studies included in reviews on scaling conducted by our team [4,5]. To recruit clinicians, policymakers, trainees, members of patient-oriented research support institutions and CBPHC organizations, we used the snowball method within our networks. Patients and public representatives received financial compensation of $100 CAD for their participation in the Delphi survey ($50 CAD for each round completed on REDCap). No financial compensation was offered to the other categories of panellists.

### Data Collection

**Data collection instruments.** 

Data collection instruments included a sociodemographic questionnaire and a survey containing the items selected by the steering committee, organized according to their respective domains along with the CVI and CVR scales for assessing the content validity of each item [20,24,25]. The CVI measures the relevance and clarity of each item using two Likert-type scales: a 4-point scale for relevance (1 = “not relevant,” 2 = “unable to assess relevance without item revision” or “item requires substantial revision,” 3 = “relevant but needs minor alteration,” and 4 = “very relevant and succinct”); and a 4-point scale for clarity (1 = “not clear,” 2 = “needs some revision,” 3 = “clear but needs minor revision,” and 4 = “very clear”). The CVR measures the necessity of each item using a 3-point Likert-type scale (1 = “not necessary,” 2 = “useful but not essential,” and 3 = “essential”). Both the sociodemographic questionnaire and the item validity survey were available in French and English. The items and domains included in the survey were translated using a modified cross-cultural adaptation process [34]. Questionnaires and surveys used in both rounds are available in S5 File.

### Delphi consensus procedure

Once registered on the REDCap platform, panellists signed a consent form and were anonymized on the system, being identified only by a code number. The two successive Delphi surveys were sent automatically by REDCap. In each round, panellists were asked to submit the completed survey within two weeks and a reminder email was sent on the day of the deadline with a seven-day extension. In the first round, we collected panellists’ sociodemographic information (e.g., country, sex, areas of expertise, primary occupational role). Both rounds one and two used the CVI and the CVR scales to achieve consensus on the content validity of the items. The first round also included open-ended questions to gather comments and suggestions from the panellists, and we encouraged them to suggest new items for areas they found lacking. In the second round, panellists were presented with the CVR and CVI median scores for items that had not reached the minimum thresholds to establish content validity in the first round. They were invited to confirm or revise their evaluations using the same Likert-type scales. In addition, panellists assessed the new items that had been suggested during the first round. Although comments were still invited in the second round, no further new items could be proposed at this stage.

### Data analysis

Frequencies were calculated and descriptive analysis of sociodemographic data performed using Excel. CVI and CVR scores were calculated by a biostatistician using SAS 9.4 following standard procedures recommended in the literature [20,24,25]. For relevance and clarity ratings, a proportion representing the number of panellists giving a high rating of 3 or 4 for a given item was divided by the total number of panellists having evaluated that item [20,24,25]. We considered that an item’s relevance and clarity were established when 80% (0.80) of panellists gave the item a high rating (3 or 4 on a 4-point scale). Items that scored less than 0.80 in the first round were sent to the second for re-evaluation. Items that scored less than 0.80 for relevance and clarity in the second round were excluded. We applied the formula (*Ne – N/2*)/ (*N/2*) to establish an item’s necessity, where *Ne* is the number of panellists indicating an item as “essential” and *N* is the total number of panellists [20,24,25]. This indicator varies between 1 and −1, and a score over 0.42 indicates an adequate score [35]. Items that scored less than 0.42 for necessity in the first round were sent to the second for revaluation. Items that scored less than 0.42 for necessity in the second round were excluded.

The qualitative data (justifications for exclusions, wording suggestions) was managed in Excel alongside the CVI and CVR item scores. No thematic analysis was necessary since comments were on specific items. In response to comments, the wording of some items was changed and the new suggested items that reached an adequate score for CVR and CVI were added.

### Phase 4: Building the ISSaQ instrument and writing the manual

The research team drafted the final version of the ISSaQ 4.0 instrument and an operating manual. We based the manual on the structures of existing manuals of published scalability assessment instruments [6,36]. First, we refined the description of each scalability domain. We then gathered the items for which content validation was established within their corresponding domains. Words and terms indicated by panellists as difficult for laypeople to understand were replaced with simpler language or else highlighted and included in a glossary. We included in the instrument a 7-point scale to allow users to indicate the extent to which their innovations meet scalability criteria, where 1 represents “Strongly disagree” and 7 represents “Strongly agree.” However, we mentioned in the manual that while the instrument can help them identify gaps and build scalability into their innovations, it does not provide a recommended minimum score for proceeding with scaling.

For PPI in this phase, we invited a patient partner and two scaling practitioners to collaborate with us. We presented them with the instrument and its manual, recorded their suggestions, comments, and wording revisions, and synthesized this feedback into summary reports. Decisions on their integration into the instrument and manual were made by consensus among the research team members. Finally, the instrument and manual were translated into English using a modified cross-cultural adaptation technique [34].

### Deviations from protocol

We did not recruit patients and clinicians in health and social services as originally planned.

## 3. Results

We report the main results from each phase and significant changes made to domains and items in each phase. The steering committee was recruited between November 2021 and January 2022. Selection of promising items occurred between January 2022 and April 2022. Delphi data were collected from November 2022 to January 2023. The final ISSaQ 4.0 instrument was prepared and the manual completed by March 2023. All data collection instruments and anonymized data are available in Additional Files.

### Phase 1: Steering committee characteristics and input

The steering committee included one patient partner, two policymakers with expertise in scaling, and one academic expert in health measures. The committee never met in person but communicated through group emails. The research team also held individual meetings with each steering committee member to discuss specific questions. The committee began by providing their feedback on the research question and the protocol. They then validated each subsequent phase of the project, including the selection of domains and items. The patient partner member of the steering committee also participated, alongside the researcher leading the project, in an international conference to disseminate the project results. This participation strengthened their connection and promoted further exchanges on the topic of PPI in scaling.

### Phase 2: Selection of domains and items

#### Selection by the research team.

Of the 12 scalability domains presented in the 2022 systematic review, the research team selected 11 for validation by the steering committee [5]. Items from the 12th domain (entitled “Other”) were distributed among the other domains, whose constructs were clearly defined. Of the 320 items collected from the 2022 systematic review, independent reviewers excluded 42 duplicates and 218 items that did not meet the 16 criteria designed to evaluate items for preference-based instruments [29]. Of the remaining 60 items, 17 were merged due to their similarity to other items. The research team’s selection process resulted ultimately in 43 items.

#### Contribution of the steering committee.

The feedback from the steering committee focused mainly on the separation of domains that contained two or more aggregated constructs. They proposed dividing the domain “Potential for implementation fidelity and adaptation of the innovation” into two distinct domains, one for fidelity of the innovation and one for adaptability of the innovation. They also recommended separating the domain “Potential reach and acceptability to the target population” into three domains focusing on coverage, acceptability, and adoption. In addition, the wording of the domains was revised based on the Committee’s suggestions. See Table 1 for the evolution of the scalability domain titles throughout the phases of the project.

**Table 1:** Evolution of the scalability domains.

Ben Charif et al., 2022	Phase 2 – After Steering Committee validation	Phase 3 - After eDelphi/ ISSaQ 4.0
(D1) Health problem addressed by the innovation	(D1) Health problem	(D1) Social or health issue addressed by the scaling
(D2) Development process of the innovation	(D2) Scaling development	(D2) Development of the scaling
(D3) Innovation characteristics	(D3) Characteristics of the innovation	(D3) Characteristics of the innovation being scaled
(D4) Strategic, political or environmental context of the innovation;	(D4) Strategic, political or environmental context of the scaling	(D4) Political context for scaling
(D5) Evidence available for effectiveness of the innovation	(D5) Evidence available for effectiveness of the innovation	(D5) Effectiveness of the innovation being scaled
(D6) Innovation costs and quantifiable benefits	(D6) Scaling costs and quantifiable benefits	(D6) Costs of the scaling
(D7) Potential for implementation fidelity and adaptation of the innovation	(D7) Implementation fidelity of the innovation	Excluded
	(D8) Adaptability of the innovation	(D7) Adaptability of the innovation being scaled
(D8) Potential reach and acceptability to the target population	(D9) Coverage of the innovation	(D8) Coverage of the scaling
	(D10) Acceptability of innovation at scale	(D9) Acceptability of the innovation being scaled
	(D11) Adoption of innovation at scale	Excluded
(D9) Delivery setting and workforce	(D12) Scaling environment	(D10) Scaling setting
(D10) Implementation infrastructure required for scale-up	(D13) Infrastructure required for scaling	(D11) Infrastructure required for scaling
(D11) Sustainability (i.e., longer-term outcomes of the scale-up)	(D14) Sustainability	(D12) Sustainability of the scaling
(D12) Others	–	–

**Fig 2:**
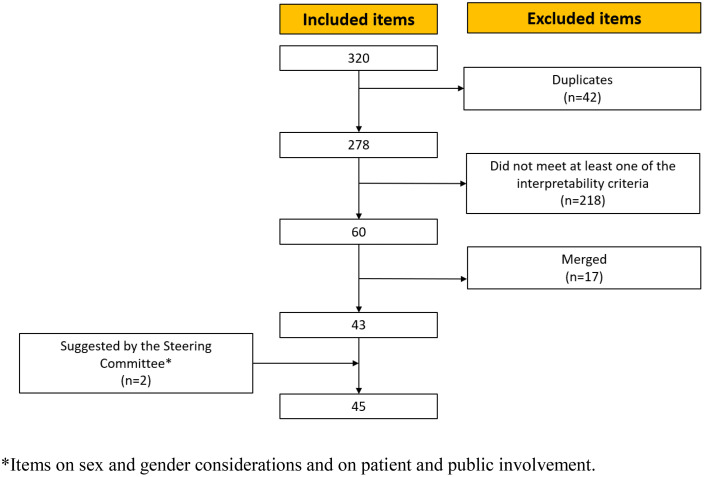
Phase 2: Item selection flow.

Following the principles of patient-oriented research [37] and sex and gender science, [38,39] the research team also suggested that the steering committee add an item on sex and gender considerations and another on patient and public involvement. These suggestions were accepted, resulting in a final list of 45 items to be assessed for content validity during the online Delphi (Fig 2).

### Phase 3: The online Delphi

#### Recruitment.

In total, 123 people were invited to participate in the Delphi. They were mostly from Canada (n = 52), the United States (n = 29), and the United Kingdom (n = 12). Invitees were primarily scaling experts (researchers or authors of published scaling studies) (n = 88), along with patient and public representatives (n = 11), and policymakers (n = 8). Telephone calls with patients and public representatives prior to the Delphi were essential to ensure their understanding of the subject and to build trust with the researchers.

#### Participant characteristics.

**Fig 3:**
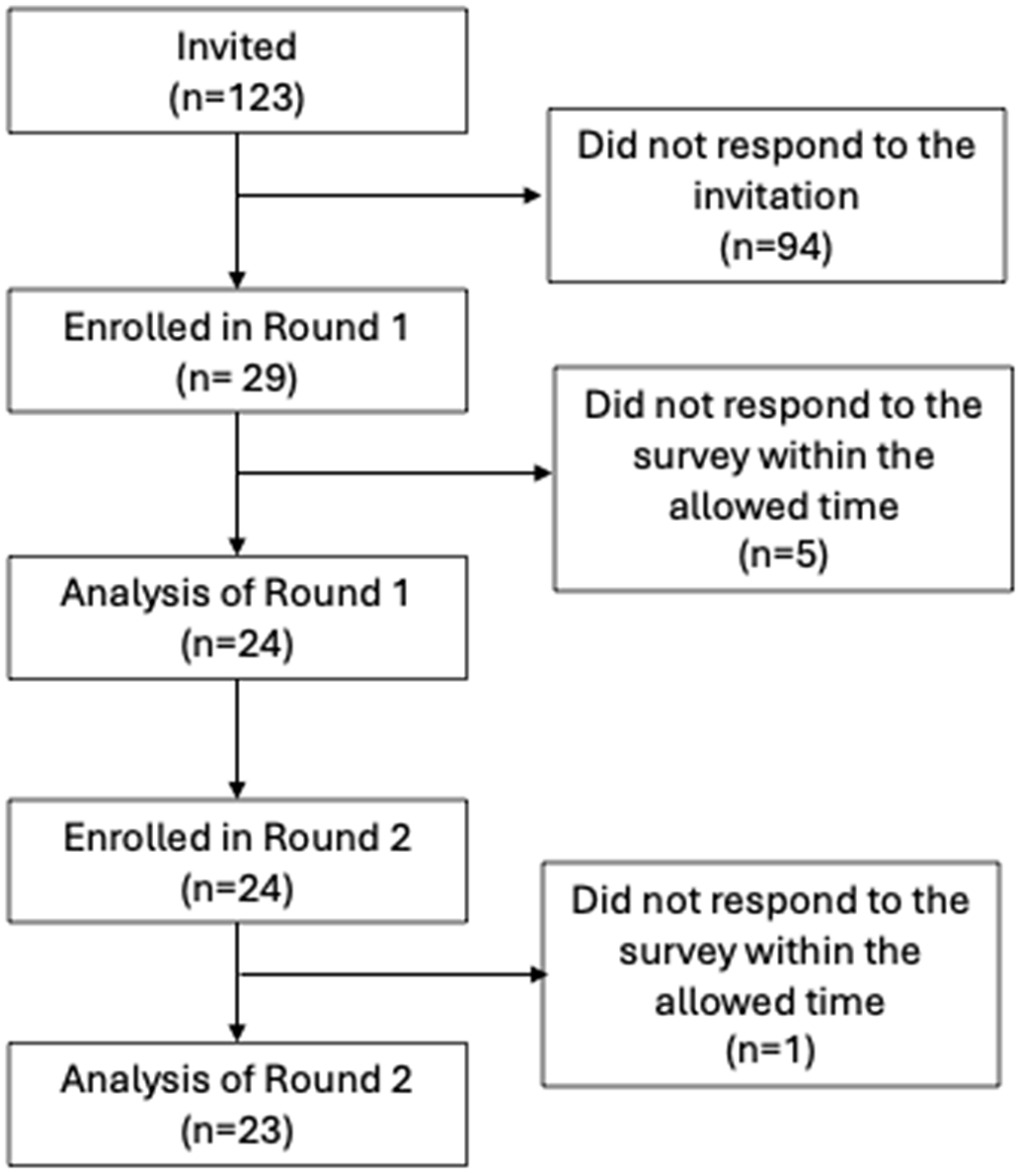
Phase 3: Panellists flowchart.

Twenty-nine panellists agreed to participate and signed the consent form. One scaling expert did not complete the first round. Twenty-three panellists completed both rounds within the allotted time (Fig 3). The sociodemographic characteristics of the Delphi panellists are presented in Table 2.

**Table 2:** Sociodemographic characteristics of Delphi panelists.

Sociodemographic characteristics	n = 24
**Sex**	
Female	13 (54.2%)
Male	11 (45,8%)
**Age (in years)**	
45–54	7 (29.2%)
65–74	5 (20.8%)
35–44	4 (16.7%)
55–64	4 (16.7%)
25–34	3 (12.5%)
75+	1 (4.2%)
**Country of residence**	
Canada	19 (79.2%)
France	2 (8.3%)
Benin	1 (4.2%)
Germany	1 (4.2%)
Mexico	1 (4.2%)
**Racial and ethnic identification**	
Caucasian	17 (70.8%)
African heritage	3 (12.5%)
Latin American	2 (8.3%)
Arab	1 (4.2%)
Multiethnic or Multiracial	1 (4.2%)
**First language**	
French	13 (54.2%)
English	4 (16.7%)
Other than French or English	7 (29.2%)
**Highest level of formal education**	
University degree	24 (100%)
Expertise*	
Community-based primary health care (CBPHC)	13 (54.2%)
Knowledge translation or implementation science	13 (54.2%)
Patient-oriented research	12 (50.0%)
Scaling, spread, or scalability	9 (37.5%)
Other expertise	3 (12.5%)
**Role in Delphi survey**	
Patient or public representatives	7 (29.1%)
Researchers	6 (25.0%)
Family physicians	3 (12.5%)
Authors of scaling studies	2 (8.3%)
Trainees	2 (8.3%)
Members of patient-oriented research support institutions	2 (8.3%)
Policy decision makers	2 (8.3%)

* Non-exclusive categories.

#### Content validity.

In the first round, of the 45 items assessed for content validity, 17 received low validity scores in at least one of the relevance, clarity, or necessity criteria. Due to a setup error in REDCap, one item was not assessed for relevance and clarity in the first round, but it was evaluated in the second. Additionally, panelists suggested five new items. These addressed considerations of national and local legislation; the disadvantages of not scaling; the alignment of the scaling intervention with global health policies, plans, and priorities; alignment with global health needs; and equity.

In the second round, in which panellists were asked to rate a total of 23 items, 15 items were excluded for receiving low validity scores in at least one of the relevance, clarity, or necessity criteria. Excluded items were in the following domains: Development of the scaling (n = 1), Characteristics of the innovation being scaled (n = 2), Political context for scaling (n = 3), Scaling fidelity (n = 3), Coverage of the scaling (n = 1), Adoption of the innovation at scale (n = 1) and, Sustainability of the scaling (n = 1). The item on sex and gender considerations in scaling was excluded by panellists, however the research team decided to retain it to address harmful gaps in health research and policy [38,40,41]. For example, scaling a generic assistive technology innovation for the elderly will have different impacts on men and women or people of diverse genders given that arthritis affects more women than age-matched men and that dexterity impairment affects more men than age-matched women [42]. The three new items included were on considerations of national and local legislation, the disadvantages of not scaling, and equity.

#### Included domains.

Two domains were excluded from the initial list of 14 domains. None of the items in the “Scaling fidelity” domain reached the consensus threshold, and therefore this domain was excluded. One panellist commented, “Questions on fidelity are problematic... Strict fidelity can be an obstacle to successful and sustainable scale up as adaption of the interventions is usually necessary to address changing contexts, target audiences, changes over time.” Only one item in the “Adoption of Innovation at Scale” domain was recommended for inclusion. After discussion, the research team decided to move this remaining item to the “Coverage of Scaling” domain. Scalability domains included are presented in Table 1.

#### Included items.

After two rounds of the online Delphi, content validity was established for 36 items and these were recommended for inclusion in the ISSaQ 4.0. One item was included based on the research team’s judgement of its importance (sex and gender considerations in scaling), despite not being considered essential by the panellists. These 37 items were distributed among 12 scalability domains. The summary of scores for relevance, clarity, and necessity for each item in each round, along with the comments and suggestions from the panellists, is presented in Table 3 and in S6 File.

**Table 3:** Items scores and final decisions.

Item ID	Item description	First round			Second round			Final Decision
		CVI Relevance ≥ 0.80	Clarity ≥ 0.80	CVR Necessity ≥ 0.42	CVI Relevance ≥ 0.80	Clarity ≥ 0.80	CVR Necessity ≥ 0.42	
**(D1) Health problem**								
Item 1	The innovation addresses a relevant health problem.	0.96	0.92	0.92				Included
Item 2	Key stakeholders have explicitly requested scaling of this innovation.	0.96	0.88	0.58				Included
Item 3	Target populations have explicitly requested scaling of this innovation.	1.00	0.96	0.58				Included
**(D2) Scaling development**								
Item 4	The development of the scaling is informed by a theory, a model or framework.	0.75	1.00	0.36	0.86		−0.04	Excluded
Item 5	Stakeholders have given their feedback on the scaling.	0.96	0.83	0.84				Included
Item 6	Target populations have given feedback on the scaling.	0.96	0.88	0.83				Included
**(D3) Characteristics of the innovation**								
Item 7	The stakeholders concerned share a common vision of what is to be scaled (its goal).	0.92	0.88	0.75				Included
Item 8	The intervention ensures continuity of care in a wide range of services.	0.56	0.71	−0.08	0.62	0.62	−0.39	Excluded
Item 9	The innovation is simple and easy to understand for the target population.	0.96	0.96	0.60				Included
Item 10	The innovation is sex- and gender-sensitive.	0.88	0.92	0.33			0.05	Excluded
Item 11	The innovation respects indigenous communities, visible minorities and their culture[s].	0.96	0.84	0.58				Included
**(D4) Strategic, political or environmental context of the scaling**								
Item 12	The innovation is consistent with existing national health policies, plans and priorities.	0.76	0.84	0.12	0.86		0.13	Excluded
Item 13	The innovation addresses needs in government health programs.	0.79	0.78	0.08	0.90	0.86	0	Excluded
Item 14	The innovation complies with policy guidelines in the target setting.	0.84	0.80	0.30			0.27	Excluded
Item 15	The political obstacles to the scaling of this innovation can be overcome.	0.72	0.68	0.20	1.00	0.86	0.65	Included
**(D5) Evidence available for effectiveness of the innovation**								
Item 16	There are data on the effectiveness of the innovation.	0.92	0.92	0.92				Included
Item 17	The advantages of the innovation and its positive impact on the health of individuals and communities are visible and can be easily demonstrated using evidence-based data.	1.00	0.96	1.00				Included
Item 18	There are data on the negative impacts of the innovation.	0.87	0.79	0.42		0.86		Included
**(D6) Scaling costs and quantifiable benefits**								
Item 19	There are data on financial and human resources [full costs] needed to scale the innovation.	0.92	0.96	0.75				Included
Item 20	The innovation requires human and financial resources that can reasonably be expected to be available during the scaling.	0.96	0.92	0.65				Included
Item 21	There are data on the cost-effectiveness of the innovation compared to existing equivalent innovations or alternatives.	0.83	0.96	0.42				Included
**(D7) Implementation fidelity of the innovation**								
Item 22	There are data on fidelity of the innovation regarding its principles and outcomes.	0.79	0.54	0.04	0.76	0.52	0.22	Excluded
Item 23	The fidelity of the innovation can be maintained at scale.	0.92	0.71	0.33		0.81	0.39	Excluded
Item 24	The fidelity of the innovation can be monitored at scale.	0.96	0.83	0.33			0.22	Excluded
**(D8) Adaptability of the innovation**								
Item 25	There are data on the adaptability of the innovation.	0.88	0.71	0.42		0.86		Included
Item 26	Requirements for local adaptations of the innovation (into a new context) have been considered.	1.00	0.92	1.00				Included
Item 27	The innovation can be (or has been) adapted for scaling without altering its fundamental characteristics, goals and outcomes.	0.92	0.92	0.58				Included
**(D9) Coverage of the innovation**								
Item 28	There is a clear definition of the target population (e.g., who will be covered by the scaled innovation and what are their attributes).	0.96	0.83	0.92				Included
Item 29	There are data on the reach of the innovation among the people involved (numerator & denominator).	0.83	0.74	0.17		0.86	0.3	Excluded
Item 30	The scaling of the innovation has the potential to reach the intended target population.	0.92	1.00	0.58				Included
**(D10) Acceptability of innovation at scale**								
Item 31	There are data on the acceptability of the innovation.	0.88	0.75	0.18		0.95	0.83	Included
Item 32	The innovation is presented appropriately using ideas and language that are meaningful to the target populations.	0.96	1.00	0.58				Included
**(D11) Adoption of innovation at scale**								
Item 33	There are data on the adoption of the innovation considering the number of units (people, services, etc.) expected to adopt the innovation and the real number that adopted it.	0.88	0.92	0.33			0.5	Included
Item 34	There are data on the target population’s intention to adopt the innovation.	0.83	0.92	0.04			0.09	Excluded
**(D12) Scaling environment**								
Item 35	The innovation has been tested in the type of environment in which it is to be scaled.*			0.57	0.9	0.81		Included
Item 36	Local multi-stakeholder partnerships have been established to support the scaling.	0.96	0.92	0.58				Included
Item 37	Appropriately trained personnel exist and are available for scaling [in target sites].	1.00	1.00	0.75				Included
**(D13) Infrastructure required for scaling**								
Item 38	There are data on the feasibility of the innovation.	0.83	0.92	0.67				Included
Item 39	Infrastructure requirements for scaling the innovation are achievable.	0.96	0.92	0.75				Included
Item 40	The organizational infrastructure required is available for scaling the innovation.	0.96	0.92	0.67				Included
Item 41	Structures are in place for monitoring the scaling process.	0.96	0.92	0.65				Included
Item 42	Structures are in place for evaluating the scaling process.	0.96	1.00	0.67				Included
**(D14) Sustainability**								
Item 43	The sustainability (maintaining the scaling on a lasting basis) has been considered.	1.00	0.88	0.57				Included
Item 44	The human and financial resources required for scaling are sustainable.	0.87	0.87	0.45				Included
Item 45	There are data on how long the innovation can it be sustained at scale.	0.74	0.65	−0.04	0.81	0.71	0.22	Excluded
New suggested items								
Item 46	The scaling of the innovation follows guidelines to ensure equity, diversity and inclusion of marginalized groups in society.				1	0.94	0.74	Included
Item 47	The scaling of the innovation complies with national and local legislation.				0.88	0.88	0.48	Included
Item 48	The scaling of the innovation is consistent with existing global health policies, plans and priorities.				0.88	0.71	−0.2	Excluded
Item 49	The scaling of the innovation addresses needs in global health.				0.94	0.82	0.2	Excluded
Item 50	The disadvantages of not scaling have been considered.				0.88	0.82	0.55	Included

* Due to an error in how the questionnaire was set up on the REDCap platform, this item could not be assessed for relevance and clarity in the first round. However, it was assessed in the second round.

### Phase 4: The ISSaQ 4.0 instrument and manual

The research team wrote the first draft of ISSaQ 4.0 and its manual based on the Delphi results. The qualitative data informed the research team’s decisions on wording revisions and improved domain descriptions. Patients and public representative panellists suggested including a figure illustrating the process of scaling in the manual. A patient partner who reviewed the document suggested adding a glossary for the main terms used in the item wording and domain descriptions (e.g., “innovation,” “scaling,” “underserved populations,” and “adaptability”). She also made suggestions about wording that was more accessible to laypeople. The two scaling teams who tried out a preliminary version of the ISSaQ 4.0 to assess the scalability of their innovations approved the instrument manual; however, they noted that the instrument was lengthy and would be user-friendly only for those with a deep understanding of the innovation being scaled. This information was used to improve the manual. Both also said the ISSaQ 4.0 fosters collaboration and would be better used by a team.

In developing the ISSaQ 4.0 with PPI, we encountered some challenges, particularly because scaling concepts are not easily communicable to laypeople and academics and policymakers tend to use jargon when discussing the topic. To overcome these challenges, we followed the recommendations of the patient partner on the team who helped us find synonyms for the word “scaling” and develop metaphors and fictional examples to explain the concept.

The final draft of ISSaQ 4.0 was completed on March 31, 2023. It included 37 items distributed among 12 scalability domains (Table 1). The manual explains when and how to use the instrument, including how to use the response scales. Both instrument and manual are available in both English and French in S7 File and on the website of Quebec’s patient-oriented research support institution (Unité SSA Québec) [43].

## 4. Discussion

In this multiphase study we involved patients and the public at each phase alongside experts from diverse backgrounds to develop a scalability assessment instrument to help scaling teams make informed decisions about scaling their innovations in health and social services. The content validity of the items included in the instrument was established by consensus. All items in the domains on use of scaling frameworks and on fidelity were excluded. Conversely, all items in the domain on adaptability of the innovation to be scaled were unanimously recognized as relevant, clear, and necessary. The ISSaQ 4.0 instrument includes five new scalability assessment items not present in previously published scalability assessment instruments [5]. These items address sex and gender considerations in scaling decisions, PPI in scaling, consideration of national and local legislation, potential disadvantages of not scaling, and equity. Of these, two were suggested by the research team and validated by the steering committee while three were proposed by Delphi panellists. Our results led to the following observations.

First, patient and public representatives were keen to collaborate in our study, as reflected by the high number who accepted the invitation to participate (7 out of 11 invited), and by their full retention through both rounds of the Delphi (7 out of 7) (Fig 3). Furthermore, our study demonstrated that patients and the public are important collaborators alongside other stakeholders in designing scaling instruments and interventions, confirming the findings of a previous scoping review published on this topic [2]. This also aligns with other findings that co-production of research in general, i.e., the active involvement of users and beneficiaries, is a crucial predictor of success in moving scientific results into action and improves the quality of the evidence [44]. Indeed, in our study the collaboration of patients and the public was critical to increasing the accessibility of the ISSaQ 4.0 instrument. Their contributions led to the inclusion of illustrations, plain language, and a glossary in the instrument manual, enabling scaling teams to better engage with patients and the public in planning to scale their interventions. Their contributions helped us address a challenge frequently highlighted in the literature, which emphasizes that scaling concepts are often difficult to communicate [2]. Thus, we conclude that PPI in scaling studies and interventions is feasible and may help overcome communication barriers while contributing to the design of more inclusive and effective scaling strategies.

Second, the panellists excluded the item suggesting that the development of scaling must be informed by a theory, model, or framework [45]. The exclusion of the item conflicts with data reported in an umbrella review on scaling which found that reviews on frameworks were among the three most published types of reviews, revealing their importance in scaling science [7]. Despite its importance in scaling science, scaling practitioners who participated in the Delphi did not find it helpful in assessing scalability. They cited their own experience in scaling to justify this exclusion, emphasizing the importance of flexibility and of not forcing every process into a framework, noting that in reality few scaling decisions are informed by a theory of scaling, and that “innovative healthcare is often done in fields where no proper theory is available.” We therefore excluded the item on frameworks, but in keeping with findings of earlier reviews, we noted in the description of the “Scaling development” domain that the literature suggests that frameworks are crucial for scaling planning [2,45]. Similar reasons were given for the exclusion of items from the “innovation fidelity” domain. Despite the importance of fidelity in determining the initial validity of an intervention and in attributing its effects, this item received low scores in the context of scaling [46]. Panellists noted that while fidelity to the expected positive outcomes of the scaled innovation is important, strict adherence to the innovation’s features and characteristics is not. Some indeed found the concept of fidelity contradictory, as scaling requires rather the adaptation of innovations for greater impact. Panellists again highlighted flexibility and adaptability as key determinants of an innovation’s scalability.

Third, ISSaQ 4.0 includes five new items that were not found in previously published scalability assessment instruments [5]. One of the new items, related to asking whether the innovation will comply with national and local legislation when scaled, is of key importance to feasibility as well as the need to conduct a dynamic evaluation throughout the scaling process, since the context may change after the beginning of the scaling intervention, including legislation and regulations [47]. One panellist described an attempt to scale up a smoking cessation program for schools in which the regulations in the target context had to be changed to allow school nurses to administer nicotine. At the macro level, international scaling studies have shown that alignment with government health priorities can result in increased government ownership and enhance the ability to achieve scaling [48]. This item also reinforces the items on collaboration with scaling stakeholders at every level [49]. National and local legislation should be considered before scaling, but also monitored during the scaling process as changes in policy or legislation can have a major impact on feasibility and sustainability [50].

The four other new items reflect the goal of scaling health and social innovations to advance social justice and reduce health inequalities [2,3]. The proposed item relating to the disadvantages of *not* scaling led scaling teams to note that in the interests of health equity, if an innovation has the potential to benefit a larger portion of the population, there may be a moral obligation to scale it [16]. An example would be rape crisis centres that are accessible to women in urban areas but not to women in more remote rural areas [51]. In this sense, in spite of challenges and barriers, additional efforts should be made to ensure that underserved groups can access these innovations and health inequalities are not perpetuated. Indeed, another newly suggested item explicitly recognizes equity as a key component of scaling in health and social services. Equity strategies should be considered from the conceptualization of innovations to be scaled to the implementation and evaluation of the scaling. The two other new items on PPI and sex and gender are consistent with this principle. As our study on instrument development and others have demonstrated, considering patient and public voices in health research and health systems is essential for achieving better and more equitable health impacts [2,4,13]. Moreover, a systematic review has shown that failure to recognize this is a barrier to successful scaling [4]. Addressing sex and gender issues in scaling in health and social services is also essential for closing knowledge gaps in health research and redressing consequent health inequities [13,41,52,53]. Disease manifestations and outcomes differ in important ways between the sexes: twice as many women as men suffer from depression, for example, and three times as many men as women commit suicide [54,55]. Failing to recognize these differences can be life-threatening. Clinical standards for ischemic heart disease, the number one killer of U.S. and European populations, that were based on male psychopathology resulted in decades of misdiagnosis or underdiagnosis of women and gender-diverse individuals. Despite the low ratings for the item on considering sex and gender when scaling, the research team therefore chose to retain it.

## 5. Limitations

Our study had a geographical recruitment bias, as the majority of Delphi panellists were from Canada. However, our approach was grounded in a systematic review of scalability assessment instruments that applied no exclusion criteria based on country. In addition our research team, which included scaling experts from South America and Africa as well as high-income countries, brought invaluable international experience on the topic.

## 6. Conclusions

The ISSaQ 4.0 scalability assessment instrument and its manual are available in both French and English. The instrument includes 37 items distributed across 12 scalability domains. Scaling teams can use this instrument to ensure their innovations are designed for scalability from the outset, to support the planning of future scaling efforts, or retrospectively to explore why scaling efforts were successful or not.

The development of the instrument followed best practices for developing and validating scales in health research while closely involving patients and public representatives. Our development process resulted in new instrument that suggests equity is a key ingredient of scalability [18,19]. This means that scaling teams who choose to use ISSaQ 4.0 to assess the scalability of their innovations and make decisions about scaling will ground their interventions in the public good and move healthcare systems towards social justice. They will be oriented to adapting their innovations to the visions and contexts of local beneficiaries, and have an impact on people whose health differences and needs have historically been neglected.

## Supporting information

S1 File Gripp2 Checklist. (DOCX)

S2 File ACCORD. (DOCX)

S3 File Selecting items. (DOCX)

S4 File Items validation. (DOCX)

S5 File Delphi survey. (PDF)

S6 File Delphi Results. (XLSX)

S7 File ISSaQ 4.0 tool. (DOCX)
