# Supplementary material for: Patient and public involvement in developing and validating an instrument for assessing the scaling potential of innovations in health and social services: A consensus study
Source: PLoS One. 2025 Nov 26;20(11):e0336245. doi: 10.1371/journal.pone.0336245 (PMC12654926; doi:10.1371/journal.pone.0336245)
Supplement: S1 File — (DOCX) [file pone.0336245.s001.docx]

**GRIPP2 for the study**

| **Section and topic** | **Item** | **Reported on page No** |
| --- | --- | --- |
| 1: Background | Report the definition of PPI used in the study and how it links to comparable studies | p. 7 |
|  | Report the theoretical rationale and any theoretical influences relating to PPI in the study | p.6 – p.8 |
| 2. Aim | Report the aim of the study | p.8 |
| 2: Methods | Does the paper provide a clear description of methods by which patients and the public were involved? | p.8, 10, 12, 16 |
|  | Provide a description of patients, carers, and the public involved with the PPI activity in the study | p.17, 19, 21 |
| 4: Discussion and conclusions | Report the results of PPI in the study, including both positive and negative outcomes | p. 22-26 |
|  | Comment on how PPI influenced the study overall. Describe positive and negative effects | p. 22-26 |
| 5: Reflections/critical perspective | Critical comment on the study, a reflection on the things that went well and those that did not, so that others can learn from it | p.26 |
