# Supplementary material for: Patient and public involvement in developing and validating an instrument for assessing the scaling potential of innovations in health and social services: A consensus study
Source: PLoS One. 2025 Nov 26;20(11):e0336245. doi: 10.1371/journal.pone.0336245 (PMC12654926; doi:10.1371/journal.pone.0336245)
Supplement: S3 File — (DOCX) [file pone.0336245.s003.docx]

**Additional File 2**

**Interpretability criteria for selecting items (Peasgood et al., 2020)**

|  | **Criterion** | **Definition** | **Met** | **Unmet** | **Unclear** |
| --- | --- | --- | --- | --- | --- |
| 1 | The item captures the concept that is intended | The content of the item must be able to assess the concepts of the study (e.g. primary health care or scaling up). | ○ | ○ | ○ |
| 2 | The item is relevant to all members of the target population | The content of the item must be relevant to all members of the target population (e.g. researcher, provider or patient). | ○ | ○ | ○ |
| 3 | The item is worded in a manner consistent with expressions used by patients | The vocabularies used in the formulation of the item must be easily understood by the patients or must not be technical | ○ | ○ | ○ |
| 4 | The item is comprehensible, i.e. not ambiguous or poorly worded | The wording of the item must be clear and unambiguous for its interpretation. | ○ | ○ | ○ |
| 5 | The item represents a single concept, rather than a multidimensional concept | The formulation of the item must be done with a single concept, instead of a multidimensional concept. | ○ | ○ | ○ |
| 6 | The item does not contain the words ‘and’, ‘or’, or ‘because’ | Avoid the use of and, or, or because in the wording of the item. | ○ | ○ | ○ |
| 7 | The item is not likely to be vulnerable to ceiling or floor effects within the target population, i.e., it will change with innovation | The content of the item must be over time, it should not be vulnerable to ceiling or floor effects within the target population. | ○ | ○ | ○ |
| 8 | The content of the item is appropriate for the recall period | The content of the item must be appropriate throughout the process. | ○ | ○ | ○ |
| 9 | The content of the item is appropriate for the mode of administration | The content of the item must be appropriate to the method of data collection from the target population. | ○ | ○ | ○ |
| 10 | The item has corresponding response scale | The item must have corresponding response scale to the stem | ○ | ○ | ○ |
| 11 | The item is as short as possible, although not so short that comprehensibility is lost | Item should be as short as possible whilst maintaining comprehensibility to all members of the target population | ○ | ○ | ○ |
| 12 | The item does not contain negated constructs (e.g., no control, not coping) or negative answers (e.g., none of the time) | Items should not be constructed with sentences in which there is a negation (e.g., no control, no adaptation) that promotes a negative response (e.g., never). | ○ | ○ | ○ |
| 13 | The item does not ask a combination of two or more questions | Avoid items with the combination of two or more questions | ○ | ○ | ○ |
| 14 | The item does not ask excessively personal questions (may lead to missing values or annoy responders) | The item must not have exclusively personal or intrusive content which may lead to missing values ​​or annoy the respondents | ○ | ○ | ○ |
| 15 | The item is ethically appropriate | It must be ensured that the content of the items is appropriate for all participants including potentially vulnerable subgroups. | ○ | ○ | ○ |
| 16 | The item does not refer to circumstances, situations or lifestyles that may not be universal across all responders | Items should be avoided whose content is not appropriate for all participants. | ○ | ○ | ○ |

**Critères d'interprétabilité pour la sélection des items**

|  | **Critères** | **Définition** | **Oui** | **Non** | **Pas clair** |
| --- | --- | --- | --- | --- | --- |
| 1 | L'item capture le concept visé. | Le contenu de l'item doit permettre d'évaluer les concepts de l'étude (par exemple, les soins de santé primaires ou la mise à l'échelle). | ○ | ○ | ○ |
| 2 | L'item est pertinent pour tous les membres de la population cible. | Le contenu de l'item doit être pertinent pour tous les membres de la population cible (par exemple, le chercheur, le prestataire ou le patient). | ○ | ○ | ○ |
| 3 | L'item est formulé de manière à correspondre aux expressions utilisées par les patients. | Les vocabulaires utilisés dans la formulation de l'item doivent être facilement compris par les patients ou ne doivent pas être techniques. | ○ | ○ | ○ |
| 4 | L'item est compréhensible, c'est-à-dire qu'il n'est pas ambigu ou mal formulé. | La formulation de l'item doit être claire et sans ambiguïté pour son interprétation. | ○ | ○ | ○ |
| 5 | L'item représente un concept unique, plutôt qu'un concept multidimensionnel. | La formulation de l'item doit se faire avec un seul concept, au lieu d'un concept multidimensionnel. | ○ | ○ | ○ |
| 6 | L'item ne contient pas les mots "et", "ou", ou "parce que". | Évitez l'utilisation de et, ou, ou parce que dans la formulation de l'item. | ○ | ○ | ○ |
| 7 | L'item n'est pas susceptible d'être vulnérable aux effets de plafond ou de plancher au sein de la population cible, c'est-à-dire qu'il évoluera avec l'innovation. | Le contenu de l'item doit s'inscrire dans le temps, il ne doit pas être vulnérable aux effets de plafond ou de plancher au sein de la population cible. | ○ | ○ | ○ |
| 8 | Le contenu de l'item est approprié pour la période de rappel. | Le contenu de l'item doit être approprié tout au long du processus. | ○ | ○ | ○ |
| 9 | Le contenu de l'item est approprié au mode d'administration. | Le contenu de l'item doit être adapté à la méthode de collecte des données auprès de la population cible. | ○ | ○ | ○ |
| 10 | L'item a une échelle de réponse correspondante. | L'item doit avoir une échelle de réponse correspondant à la tige. | ○ | ○ | ○ |
| 11 | L'item est aussi court que possible, mais pas au point de ne plus être compréhensible. | L'item doit être aussi court que possible tout en restant compréhensible pour tous les membres de la population cible. | ○ | ○ | ○ |
| 12 | L'item ne contient pas de constructions négatives (par exemple, pas de contrôle, pas d'adaptation) ou de réponses négatives (par exemple, jamais). | Les items ne doivent pas être construits avec des phrases dans lesquelles il y a une négation (par exemple, aucun contrôle, aucune adaptation) qui favorise une réponse négative (par exemple, jamais). | ○ | ○ | ○ |
| 13 | L'item ne pose pas une combinaison de deux ou plusieurs questions. | Éviter les items comportant la combinaison de deux ou plusieurs questions. | ○ | ○ | ○ |
| 14 | L'item ne pose pas de questions excessivement personnelles (ce qui pourrait entraîner des valeurs manquantes ou ennuyer les répondants). | L'item ne doit pas avoir un contenu exclusivement personnel ou intrusif qui pourrait conduire à des valeurs manquantes ou gêner les répondants. | ○ | ○ | ○ |
| 15 | L'item est éthiquement approprié | Il faut s'assurer que le contenu des items est approprié pour tous les participants, y compris les sous-groupes potentiellement vulnérables. | ○ | ○ | ○ |
| 16 | L'item ne fait pas référence à des circonstances, des situations ou des modes de vie qui pourraient ne pas être universels pour tous les répondants. | Il convient d'éviter les items dont le contenu n'est pas approprié pour tous les participants. | ○ | ○ | ○ |

Peasgood, T., Mukuria, C., Carlton, J., Connell, J., & Brazier, J. (2020, 2020/12/07). Criteria for item selection for a preference-based measure for use in economic evaluation. *Quality of Life Research*. <https://doi.org/10.1007/s11136-020-02718-9>
