# Supplementary material for: Patient and public involvement in developing and validating an instrument for assessing the scaling potential of innovations in health and social services: A consensus study
Source: PLoS One. 2025 Nov 26;20(11):e0336245. doi: 10.1371/journal.pone.0336245 (PMC12654926; doi:10.1371/journal.pone.0336245)
Supplement: S4 File — (DOCX) [file pone.0336245.s004.docx]

**Additional File 3**

**DEVELOPMENT OF A TOOL FOR ASSESSING THE SCALABILITY OF INNOVATIONS IN COMMUNITY-BASED PRIMARY HEALTH CARE IN CANADA**

**Objective:** We seek to refine and validate the tool for assessing the scalability of innovations in community-based primary health (CBPH) with an integrated knowledge translation (iKT) approach. First, we conducted a knowledge synthesis to identify and assess tools that have already been developed and which included scalability criteria (PROSPERO ID: CRD42019107095). Now, we will use evidence from this synthesis to achieve a consensus opinion on the most promising scalability criteria for assessing the scalability of EBIs in the specific context of CBPHC.

**Methodology:** The knowledge synthesis identified a total of 21 existing tools for assessing the scalability of healthcare innovations and 320 related assessment items. These were categorized into 11 scalability components (highlighted in yellow in the table below). Our team has conducted a systematic reduction of items in which 16 selection criteria^[[1]](#footnote-1)^ were applied by two researchers in 4 decision rounds and 2 consensus meetings. Finally, we achieved a total of 35 items for starting the e-Delphi. We prepared the table below to know your opinion about them as steering committee members. The 16 selection criteria are listed at the end of the document, if necessary. We have tried to be as restrictive as possible, but in cases of doubt, we prefer to keep the item and know your decision.

**List of items for decision:**

| **Scalability Components** | **Item** | | **Decision** | **Reason for exclusion** | **Suggestions** |
| --- | --- | --- | --- | --- | --- |
| Problem |  | The outcomes delivered by the innovation address a relevant health problem. | [ ] Exclude  [ ] Include |  |  |
|  |  | There is an explicit request for scaling up the innovation by key stakeholders. | [ ] Exclude  [ ] Include |  |  |
| Development process |  | The stakeholders given input about the innovation pilot and scaling up. | [ ] Exclude  [ ] Include |  |  |
|  |  | A theory, model, or framework inform the scaling up development of the innovation. | [ ] Exclude  [ ] Include |  |  |
| Innovation characteristics |  | The innovation has respect for indigenous peoples and their culture. | [ ] Exclude  [ ] Include |  |  |
|  |  | The innovation provides continuity of care and a broad spectrum of services. | [ ] Exclude  [ ] Include |  |  |
|  |  | The innovation is convenient to use with ideas and language meaningful and easily understood by users. | [ ] Exclude  [ ] Include |  |  |
|  |  | There is a shared vision of what is to be scaled (its aim) among the relevant stakeholders of the innovation. | [ ] Exclude  [ ] Include |  |  |
| Strategic/political context |  | The innovation is consistent with existing national health policies, plans, and priorities in the setting and local context in which it will be scaled. | [ ] Exclude  [ ] Include |  |  |
|  |  | The innovation addresses needs/fills gaps in government health programs. | [ ] Exclude  [ ] Include |  |  |
|  |  | There is no political barrier to implementing this intervention. | [ ] Exclude  [ ] Include |  |  |
| Evidence of effectiveness |  | There are data on the efficacy of the innovation (i.e., testing under optimal conditions). | [ ] Exclude  [ ] Include |  |  |
|  |  | There are data on the effectiveness of the innovation (i.e., testing under optimal conditions) built using appropriate evaluative methods. | [ ] Exclude  [ ] Include |  |  |
|  |  | The innovation has been evaluated for achieving its objectives. | [ ] Exclude  [ ] Include |  |  |
| Costs & benefits |  | There are data on the cost-effectiveness of the innovation. | [ ] Exclude  [ ] Include |  |  |
|  |  | There are data on resources and their full costs needed to scale up the innovation. | [ ] Exclude  [ ] Include |  |  |
|  |  | There are data on the cost-effectiveness of the innovation compared to existing equivalent innovations or alternatives. | [ ] Exclude  [ ] Include |  |  |
|  |  | The innovation requires human and financial resources that can reasonably be expected to be available during scale up. | [ ] Exclude  [ ] Include |  |  |
| Fidelity & adaptation |  | Adaptations can be (or were able to be) made to the primary intervention in the replica context without altering its fundamental nature. | [ ] Exclude  [ ] Include |  |  |
|  |  | The fidelity of the innovation can be monitored and maintained if implemented at scale. | [ ] Exclude  [ ] Include |  |  |
|  |  | There are data on the adaptability of the innovation. | [ ] Exclude  [ ] Include |  |  |
|  |  | There are data on the fidelity of the innovation. | [ ] Exclude  [ ] Include |  |  |
|  |  | The scaling up program considers what is needed to tailor the innovation locally considering the characteristics of the population and the geographical, socioeconomic, and cultural contexts. | [ ] Exclude  [ ] Include |  |  |
| Reach & acceptability |  | There are data on the acceptability of the innovation among key stakeholders. | [ ] Exclude  [ ] Include |  |  |
|  |  | There are data on the adoption of the innovation (numerator & denominator). | [ ] Exclude  [ ] Include |  |  |
|  |  | There are data on the reach of the innovation (numerator & denominator). | [ ] Exclude  [ ] Include |  |  |
|  |  | The innovation has the potential to reach the intended target population at scale. | [ ] Exclude  [ ] Include |  |  |
| Delivery settings & workforce |  | The local multi-stakeholder partnerships are in place to support the scaling up of the innovation. | [ ] Exclude  [ ] Include |  |  |
|  |  | There are the skills, competencies, and trained workforce required to scale up innovation. | [ ] Exclude  [ ] Include |  |  |
| Implementation infrastructure |  | The implementation infrastructure  requirements of the innovation are  feasible for scale up. | [ ] Exclude  [ ] Include |  |  |
|  |  | There are data on the feasibility of the innovation. | [ ] Exclude  [ ] Include |  |  |
|  |  | There are structures in place to monitor and evaluate the scaling up process. | [ ] Exclude  [ ] Include |  |  |
| Sustainability |  | Sustainability (maintaining the scaled up innovation on a lasting basis) has been considered. | [ ] Exclude  [ ] Include |  |  |
|  |  | There are data on the maintenance of the innovation that include how long can it be sustained. | [ ] Exclude  [ ] Include |  |  |
|  |  | The human and financial resources required to implement the innovation at scale is sustainable. | [ ] Exclude  [ ] Include |  |  |

**Interpretability criteria for selecting items (Peasgood et al., 2020)**

|  | **Criterion** | **Definition** |
| --- | --- | --- |
| 1 | The item captures the concept that is intended | The content of the item must be able to assess the concepts of the study (e.g. primary health care or scaling up). |
| 2 | The item is relevant to all members of the target population | The content of the item must be relevant to all members of the target population (e.g. researcher, provider or patient). |
| 3 | The item is worded in a manner consistent with expressions used by patients | The vocabularies used in the formulation of the item must be easily understood by the patients or must not be technical |
| 4 | The item is comprehensible, i.e. not ambiguous or poorly worded | The wording of the item must be clear and unambiguous for its interpretation. |
| 5 | The item represents a single concept, rather than a multidimensional concept | The formulation of the item must be done with a single concept, instead of a multidimensional concept. |
| 6 | The item does not contain the words ‘and’, ‘or’, or ‘because’ | Avoid the use of and, or, or because in the wording of the item. |
| 7 | The item is not likely to be vulnerable to ceiling or floor effects within the target population, i.e., it will change with innovation | The content of the item must be over time, it should not be vulnerable to ceiling or floor effects within the target population. |
| 8 | The content of the item is appropriate for the recall period | The content of the item must be appropriate throughout the process. |
| 9 | The content of the item is appropriate for the mode of administration | The content of the item must be appropriate to the method of data collection from the target population. |
| 10 | The item has corresponding response scale | The item must have corresponding response scale to the stem |
| 11 | The item is as short as possible, although not so short that comprehensibility is lost | Item should be as short as possible whilst maintaining comprehensibility to all members of the target population |
| 12 | The item does not contain negated constructs (e.g., no control, not coping) or negative answers (e.g., none of the time) | Items should not be constructed with sentences in which there is a negation (e.g., no control, no adaptation) that promotes a negative response (e.g., never). |
| 13 | The item does not ask a combination of two or more questions | Avoid items with the combination of two or more questions |
| 14 | The item does not ask excessively personal questions (may lead to missing values or annoy responders) | The item must not have exclusively personal or intrusive content which may lead to missing values ​​or annoy the respondents |
| 15 | The item is ethically appropriate | It must be ensured that the content of the items is appropriate for all participants including potentially vulnerable subgroups. |
| 16 | The item does not refer to circumstances, situations or lifestyles that may not be universal across all responders | Items should be avoided whose content is not appropriate for all participants. |

1. Peasgood, T., Mukuria, C., Carlton, J., Connell, J., & Brazier, J. (2020, 2020/12/07). Criteria for item selection for a preference-based measure for use in economic evaluation. Quality of Life Research. https://doi.org/10.1007/s11136-020-02718-9 [↑](#footnote-ref-1)
