# Supplementary material for: Patient and public involvement in developing and validating an instrument for assessing the scaling potential of innovations in health and social services: A consensus study
Source: PLoS One. 2025 Nov 26;20(11):e0336245. doi: 10.1371/journal.pone.0336245 (PMC12654926; doi:10.1371/journal.pone.0336245)
Supplement: S5 File — (PDF) [file pone.0336245.s005.pdf]

User involvement in developing and validating an instrument for assessing the scalability of innovations in health and social services: a consensus process using the Delphi method. Corôa et al. 2024

Additional File 4

A. Heath Problem This component comprises statements related to the health problem that the scaling of the innovation addresses. In this context, it is important to take into account the perceptions of stakeholders and the target population.

A. Problème de santé Cette composante rassemble des énoncés liés au problème de santé auquel s'adresse la mise à l'échelle de l'innovation. Dans ce cadre, il est important de prendre en compte les perceptions des parties prenantes et de la population ciblée.

*Please ensure that you have answered all questions before submitting.

*The meaning of the scales can be found at the end of the page.

*Veuillez vous assurer que vous avez répondu à toutes les questions avant de les soumettre.

*La signification des échelles se trouve en fin de page.

1. The innovation addresses a relevant health problem. 1. Cette innovation répond à un problème en santé pertinent.

	1	2	3	4
Is the item important? / L'item est-il important ?	○	○	○	○
Is the item clear? / L'item est-il clair ?	○	○	○	○

	1	2	3
Is the item necessary? / L'item est-il nécessaire ?	○	○	○

2. Key stakeholders have explicitly requested scaling of this innovation. 2. Il y a une demande explicite de mise à l'échelle de l'innovation par les parties prenantes.

	1	2	3	4
Is the item important? / L'item est-il important ?	○	○	○	○
Is the item clear? / L'item est-il clair ?	○	○	○	○

	1	2	3
Is the item necessary? / L'item est-il nécessaire ?	○	○	○

3. Target populations have explicitly requested scaling of this innovation. 3. Il y a une demande explicite de mise à l'échelle de l'innovation par les populations ciblées.

	1	2	3	4
Is the item important? / L'item est-il important ?	○	○	○	○
Is the item clear? / L'item est-il clair ?	○	○	○	○

	1	2	3
Is the item necessary? / L'item est-il nécessaire ?	○	○	○

Please provide any additional comments regarding the relevance, clarity and necessity of the items in this section.

Veuillez fournir tout commentaire supplémentaire concernant la pertinence, la clarté et la nécessité des items de cette section.

La version française suit.

For each scalability statement included in our tool, we are asking you to rate: Is it relevant* considering the aim to scale a health innovation for achieving greater impact?

1 = not relevant

2 = can't assess its relevance unless item is revised, or so much revision necessary that it would no longer be relevant

3 = relevant but needs minor alteration

4 = very relevant and succinct

Is it clear to all potential end users of the tool?

1 = not clear

2 = item needs some revision

3 = clear but needs minor revision

4 = very clear

Is it necessary* to be included in a tool for assessing the scalability of health innovations?

1 = not necessary

2 = useful but not essential

3 = essential

*Please note that while a relevant statement bring an important information, which would be interesting to consider regarding the scalability, a necessary statement express an information that is indispensable to decide about the scaling of a health innovation.

Open-ended (free text) questions

There are open-ended (free text) questions to collect comments and suggestions at the end of each section. You can suggest other assessment items and we ask you to explain why they would be useful. We also encourage you to provide reasons for your decision when you give the item a low score.

NOTE: If you cannot complete the survey in one setting, a "save and continue later" feature is available (button located at the bottom of each survey page). Once you have logged out, to return, simply click on the link received by email to return to the survey.

----Pour chaque énoncés inclus dans notre outil, nous vous demandons d'évaluer : Est-il important* compte tenu de l'objectif de mettre à l'échelle une innovation en santé pour obtenir un plus grand impact ?

1 = non pertinent

2 = impossible d'évaluer la pertinence sans révision de l'item, ou l'item a besoin d'une telle révision qu'il n'est même pas pertinent

3 = pertinent mais nécessite des modifications mineures

4 = très pertinent et succinct

Est-il clair pour tous les potentiel.le.s utilisateur.rice.s de l'outil ?

1 = pas clair

2 = l'item nécessite une certaine révision

3 = clair mais nécessite une révision mineure

4 = très clair

Est-il nécessaire* d'être inclus dans un outil dont l'objectif est d'évaluer le potentiel de mise à l'échelle des innovations en santé ?

1 = non nécessaire

2 = utile mais pas essentiel

3 = essentiel

*Les échelles d'évaluation seront disponibles à la fin de chaque session à titre de rappel.

*Veuillez noter que si un énoncé pertinent apporte une information importante, qu'il serait intéressant de considérer en ce qui concerne la mise à l'échelle, un énoncé nécessaire exprime une information indispensable pour décider sur la mise à l'échelle d'une innovation en santé.

Questions ouvertes (texte libre)

Vous trouverez des questions ouvertes (texte libre) à la fin de chaque section pour collecter vos commentaires et suggestions. Vous pouvez suggérer des autres items d'évaluation du potentiel de mise à l'échelle selon vos perceptions et nous vous demandons de justifier leur inclusion. Nous vous encourageons également à justifier vos décisions pour les scores faibles.

NOTE: Vous pourrez compléter le questionnaire à tout moment, en cliquant sur le lien dans votre courriel, qui vous redirigera automatiquement à l'endroit où vous étiez rendu. Une fois que vous vous êtes déconnecté, pour revenir, il suffit de cliquer sur le lien reçu par e-mail pour revenir à l'enquête.

B. Scaling development This component comprises statements related to the innovation scaling development process. This development needs to be systematic and to involve stakeholders and the target population.

B. Développement de la mise à l'échelle Cette composante rassemble des énoncés liés au processus de développement de la mise à l'échelle de l'innovation. Ce développement doit être systématique et il doit impliquer les parties prenantes et la population ciblée.

*Please ensure that you have answered all questions before submitting.

*The meaning of the scales can be found at the end of the page.

*Veuillez vous assurer que vous avez répondu à toutes les questions avant de les soumettre.

*La signification des échelles se trouve en fin de page.

4. The development of the scaling is informed by a theory, a model or framework. 4. Une théorie, un modèle ou un cadre conceptuel informent la mise à l'échelle.

	1	2	3	4
Is the item important? / L'item est-il important ?	○	○	○	○
Is the item clear? / L'item est-il clair ?	○	○	○	○

	1	2	3
Is the item necessary? / L'item est-il nécessaire ?	○	○	○

5. Stakeholders have given their feedback on the scaling. 5. Les parties prenantes ont donné leur avis sur la mise à l'échelle.

	1	2	3	4
Is the item important? / L'item est-il important ?	○	○	○	○
Is the item clear? / L'item est-il clair ?	○	○	○	○

	1	2	3
Is the item necessary? / L'item est-il nécessaire ?	○	○	○

6. Target populations have given feedback on the scaling. 6. Les populations ciblées ont donné leur avis sur la mise à l'échelle.

	1	2	3	4
Is the item important? / L'item est-il important ?	○	○	○	○
Is the item clear? / L'item est-il clair ?	○	○	○	○

	1	2	3
Is the item necessary? / L'item est-il nécessaire ?	○	○	○

Please provide any additional comments regarding the relevance, clarity and necessity of the items in this section.

Veuillez fournir tout commentaire supplémentaire concernant la pertinence, la clarté et la nécessité des items de cette section.

La version française suit.

For each scalability statement included in our tool, we are asking you to rate: Is it relevant* considering the aim to scale a health innovation for achieving greater impact?

1 = not relevant

2 = can't assess its relevance unless item is revised, or so much revision necessary that it would no longer be relevant

3 = relevant but needs minor alteration

4 = very relevant and succinct

Is it clear to all potential end users of the tool?

1 = not clear

2 = item needs some revision

3 = clear but needs minor revision

4 = very clear

Is it necessary* to be included in a tool for assessing the scalability of health innovations?

1 = not necessary

2 = useful but not essential

3 = essential

*Please note that while a relevant statement bring an important information, which would be interesting to consider regarding the scalability, a necessary statement express an information that is indispensable to decide about the scaling of a health innovation.

Open-ended (free text) questions

There are open-ended (free text) questions to collect comments and suggestions at the end of each section. You can suggest other assessment items and we ask you to explain why they would be useful. We also encourage you to provide reasons for your decision when you give the item a low score.

NOTE: If you cannot complete the survey in one setting, a "save and continue later" feature is available (button located at the bottom of each survey page). Once you have logged out, to return, simply click on the link received by email to return to the survey.

----Pour chaque énoncés inclus dans notre outil, nous vous demandons d'évaluer : Est-il important* compte tenu de l'objectif de mettre à l'échelle une innovation en santé pour obtenir un plus grand impact ?

1 = non pertinent

2 = impossible d'évaluer la pertinence sans révision de l'item, ou l'item a besoin d'une telle révision qu'il n'est même pas pertinent

3 = pertinent mais nécessite des modifications mineures

4 = très pertinent et succinct

Est-il clair pour tous les potentiel.le.s utilisateur.rice.s de l'outil ?

1 = pas clair

2 = l'item nécessite une certaine révision

3 = clair mais nécessite une révision mineure

4 = très clair

Est-il nécessaire* d'être inclus dans un outil dont l'objectif est d'évaluer le potentiel de mise à l'échelle des innovations en santé ?

1 = non nécessaire

2 = utile mais pas essentiel

3 = essentiel

*Les échelles d'évaluation seront disponibles à la fin de chaque session à titre de rappel.

*Veuillez noter que si un énoncé pertinent apporte une information importante, qu'il serait intéressant de considérer en ce qui concerne la mise à l'échelle, un énoncé nécessaire exprime une information indispensable pour décider sur la mise à l'échelle d'une innovation en santé.

Questions ouvertes (texte libre)

Vous trouverez des questions ouvertes (texte libre) à la fin de chaque section pour collecter vos commentaires et suggestions. Vous pouvez suggérer des autres items d'évaluation du potentiel de mise à l'échelle selon vos perceptions et nous vous demandons de justifier leur inclusion. Nous vous encourageons également à justifier vos décisions pour les scores faibles.

NOTE: Vous pourrez compléter le questionnaire à tout moment, en cliquant sur le lien dans votre courriel, qui vous redirigera automatiquement à l'endroit où vous étiez rendu. Une fois que vous vous êtes déconnecté, pour revenir, il suffit de cliquer sur le lien reçu par e-mail pour revenir à l'enquête.

C. Characteristics of the innovation This component comprises statements related to the characteristics of the innovation scaling that aim to ensure its integration into existing health services. The component also aims to ensure its understanding by stakeholders and the target population. These questions address ethical aspects and social norms in the context of scaling.

C. Caractéristiques de l'innovation Cette composante rassemble des énoncés liés aux caractéristiques de la mise à l'échelle de l'innovation qui visent à assurer son intégration dans les services de santé existants. Elle vise aussi à assurer sa compréhension par les parties prenantes et par la population ciblée. Ces questions portent sur les aspects éthiques et les normes sociales dans le contexte de mise à échelle.

*Please ensure that you have answered all questions before submitting.

*The meaning of the scales can be found at the end of the page.

*Veuillez vous assurer que vous avez répondu à toutes les questions avant de les soumettre.

*La signification des échelles se trouve en fin de page.

7. The stakeholders concerned share a common vision of what is to be scaled up (its goal). 7. Les parties prenantes impliquées partagent une vision commune sur ce qui signifie la mise à l'échelle (son but).

	1	2	3	4
Is the item important? / L'item est-il important ?	○	○	○	○
Is the item clear? / L'item est-il clair ?	○	○	○	○

	1	2	3
Is the item necessary? / L'item est-il nécessaire ?	○	○	○

8. The intervention ensures continuity of care in a wide range of services. 8. L'innovation assure la continuité des soins dans une vaste gamme de services.

	1	2	3	4
Is the item important? / L'item est-il important ?	○	○	○	○
Is the item clear? / L'item est-il clair ?	○	○	○	○

	1	2	3
Is the item necessary? / L'item est-il nécessaire ?	○	○	○

9. The innovation is simple and easy to understand for the target population. 9. L'innovation est facile à comprendre pour les populations ciblées.

	1	2	3	4
Is the item important? / L'item est-il important ?	○	○	○	○
Is the item clear? / L'item est-il clair ?	○	○	○	○

	1	2	3
Is the item necessary? / L'item est-il nécessaire ?	○	○	○

10. The innovation is sex- and gender-sensitive. 10. L'innovation tient compte des questions de sexe et de genre.

	1	2	3	4
Is the item important? / L'item est-il important ?	○	○	○	○
Is the item clear? / L'item est-il clair ?	○	○	○	○

	1	2	3
Is the item necessary? / L'item est-il nécessaire ?	○	○	○

11. The innovation respects indigenous communities, visible minority and their culture[s]. 11. L'innovation respecte les communautés autochtones et les minorités visibles et leur[s] culture[s].

	1	2	3	4
Is the item important? / L'item est-il important ?	○	○	○	○
Is the item clear? / L'item est-il clair ?	○	○	○	○

	1	2	3
Is the item necessary? / L'item est-il nécessaire ?	○	○	○

Please provide any additional comments regarding the relevance, clarity and necessity of the items in this section.

Veuillez fournir tout commentaire supplémentaire concernant la pertinence, la clarté et la nécessité des items de cette section.

La version française suit.

For each scalability statement included in our tool, we are asking you to rate: Is it relevant* considering the aim to scale a health innovation for achieving greater impact?

1 = not relevant

2 = can't assess its relevance unless item is revised, or so much revision necessary that it would no longer be relevant

3 = relevant but needs minor alteration

4 = very relevant and succinct

Is it clear to all potential end users of the tool?

1 = not clear

2 = item needs some revision

3 = clear but needs minor revision

4 = very clear

Is it necessary* to be included in a tool for assessing the scalability of health innovations?

1 = not necessary

2 = useful but not essential

3 = essential

*Please note that while a relevant statement bring an important information, which would be interesting to consider regarding the scalability, a necessary statement express an information that is indispensable to decide about the scaling of a health innovation.

Open-ended (free text) questions

There are open-ended (free text) questions to collect comments and suggestions at the end of each section. You can suggest other assessment items and we ask you to explain why they would be useful. We also encourage you to provide reasons for your decision when you give the item a low score.

NOTE: If you cannot complete the survey in one setting, a "save and continue later" feature is available (button located at the bottom of each survey page). Once you have logged out, to return, simply click on the link received by email to return to the survey.

----Pour chaque énoncés inclus dans notre outil, nous vous demandons d'évaluer : Est-il important* compte tenu de l'objectif de mettre à l'échelle une innovation en santé pour obtenir un plus grand impact ?

1 = non pertinent

2 = impossible d'évaluer la pertinence sans révision de l'item, ou l'item a besoin d'une telle révision qu'il n'est même pas pertinent

3 = pertinent mais nécessite des modifications mineures

4 = très pertinent et succinct

Est-il clair pour tous les potentiel.le.s utilisateur.rice.s de l'outil ?

1 = pas clair

2 = l'item nécessite une certaine révision

3 = clair mais nécessite une révision mineure

4 = très clair

Est-il nécessaire* d'être inclus dans un outil dont l'objectif est d'évaluer le potentiel de mise à l'échelle des innovations en santé ?

1 = non nécessaire

2 = utile mais pas essentiel

3 = essentiel

*Les échelles d'évaluation seront disponibles à la fin de chaque session à titre de rappel.

*Veuillez noter que si un énoncé pertinent apporte une information importante, qu'il serait intéressant de considérer en ce qui concerne la mise à l'échelle, un énoncé nécessaire exprime une information indispensable pour décider sur la mise à l'échelle d'une innovation en santé.

Questions ouvertes (texte libre)

Vous trouverez des questions ouvertes (texte libre) à la fin de chaque section pour collecter vos commentaires et suggestions. Vous pouvez suggérer des autres items d'évaluation du potentiel de mise à l'échelle selon vos perceptions et nous vous demandons de justifier leur inclusion. Nous vous encourageons également à justifier vos décisions pour les scores faibles.

NOTE: Vous pourrez compléter le questionnaire à tout moment, en cliquant sur le lien dans votre courriel, qui vous redirigera automatiquement à l'endroit où vous étiez rendu. Une fois que vous vous êtes déconnecté, pour revenir, il suffit de cliquer sur le lien reçu par e-mail pour revenir à l'enquête.

D. Strategic, political or environmental context of the scaling This component comprises statements related to existing health policies, both at the broader national level and in the specific context of the innovation. It also invites innovators to reflect on possible existing policy barriers that may reduce scalability.

D. Contexte stratégique, politique et environnemental de la mise à l'échelle Cette composante rassemble des énoncés liés aux politiques de santé existantes, que ce soit au niveau national plus large ou dans le contexte spécifique de l'innovation. De même, il invite les innovateur.rice.s à réfléchir aux éventuels obstacles politiques existants qui peuvent diminuer le potentiel de mise à l'échelle.

*Please ensure that you have answered all questions before submitting.

*The meaning of the scales can be found at the end of the page.

*Veuillez vous assurer que vous avez répondu à toutes les questions avant de les soumettre.

*La signification des échelles se trouve en fin de page.

12. The innovation is consistent with existing national health policies, plans and priorities. 12. L'innovation est conforme aux politiques, plans et priorités de santé du gouvernement.

	1	2	3	4
Is the item important? / L'item est-il important ?	○	○	○	○
Is the item clear? / L'item est-il clair ?	○	○	○	○

	1	2	3
Is the item necessary? / L'item est-il nécessaire ?	○	○	○

13. The innovation addresses needs in government health programs. 13. L'innovation répond aux besoins des programmes de santé du gouvernement.

	1	2	3	4
Is the item important? / L'item est-il important ?	○	○	○	○
Is the item clear? / L'item est-il clair ?	○	○	○	○

	1	2	3
Is the item necessary? / L'item est-il nécessaire ?	○	○	○

14. The innovation complies with policy guidelines in the target scale-up setting. 14. L'innovation est conforme aux directives politiques du milieu dans lequel elle sera mise à l'échelle.

	1	2	3	4
Is the item important? / L'item est-il important ?	○	○	○	○
Is the item clear? / L'item est-il clair ?	○	○	○	○

	1	2	3
Is the item necessary? / L'item est-il nécessaire ?	○	○	○

15. There are no political obstacles to the scaling of this innovation. 15. Il n'y a pas d'obstacle politique à la mise à l'échelle de cette innovation.

	1	2	3	4
Is the item important? / L'item est-il important ?	○	○	○	○
Is the item clear? / L'item est-il clair ?	○	○	○	○

	1	2	3
Is the item necessary? / L'item est-il nécessaire ?	○	○	○

Please provide any additional comments regarding the relevance, clarity and necessity of the items in this section.

Veuillez fournir tout commentaire supplémentaire concernant la pertinence, la clarté et la nécessité des items de cette section.

La version française suit.

For each scalability statement included in our tool, we are asking you to rate: Is it relevant* considering the aim to scale a health innovation for achieving greater impact?

1 = not relevant

2 = can't assess its relevance unless item is revised, or so much revision necessary that it would no longer be relevant

3 = relevant but needs minor alteration

4 = very relevant and succinct

Is it clear to all potential end users of the tool?

1 = not clear

2 = item needs some revision

3 = clear but needs minor revision

4 = very clear

Is it necessary* to be included in a tool for assessing the scalability of health innovations?

1 = not necessary

2 = useful but not essential

3 = essential

*Please note that while a relevant statement bring an important information, which would be interesting to consider regarding the scalability, a necessary statement express an information that is indispensable to decide about the scaling of a health innovation.

Open-ended (free text) questions

There are open-ended (free text) questions to collect comments and suggestions at the end of each section. You can suggest other assessment items and we ask you to explain why they would be useful. We also encourage you to provide reasons for your decision when you give the item a low score.

NOTE: If you cannot complete the survey in one setting, a "save and continue later" feature is available (button located at the bottom of each survey page). Once you have logged out, to return, simply click on the link received by email to return to the survey.

----Pour chaque énoncés inclus dans notre outil, nous vous demandons d'évaluer : Est-il important* compte tenu de l'objectif de mettre à l'échelle une innovation en santé pour obtenir un plus grand impact ?

1 = non pertinent

2 = impossible d'évaluer la pertinence sans révision de l'item, ou l'item a besoin d'une telle révision qu'il n'est même pas pertinent

3 = pertinent mais nécessite des modifications mineures

4 = très pertinent et succinct

Est-il clair pour tous les potentiel.le.s utilisateur.rice.s de l'outil ?1 = pas clair

2 = l'item nécessite une certaine révision

3 = clair mais nécessite une révision mineure

4 = très clair

Est-il nécessaire* d'être inclus dans un outil dont l'objectif est d'évaluer le potentiel de mise à l'échelle des innovations en santé ?

1 = non nécessaire

2 = utile mais pas essentiel

3 = essentiel

*Les échelles d'évaluation seront disponibles à la fin de chaque session à titre de rappel.

*Veuillez noter que si un énoncé pertinent apporte une information importante, qu'il serait intéressant de considérer en ce qui concerne la mise à l'échelle, un énoncé nécessaire exprime une information indispensable pour décider sur la mise à l'échelle d'une innovation en santé.

Questions ouvertes (texte libre)

Vous trouverez des questions ouvertes (texte libre) à la fin de chaque section pour collecter vos commentaires et suggestions. Vous pouvez suggérer des autres items d'évaluation du potentiel de mise à l'échelle selon vos perceptions et nous vous demandons de justifier leur inclusion. Nous vous encourageons également à justifier vos décisions pour les scores faibles.

NOTE: Vous pourrez compléter le questionnaire à tout moment, en cliquant sur le lien dans votre courriel, qui vous redirigera automatiquement à l'endroit où vous étiez rendu. Une fois que vous vous êtes déconnecté, pour revenir, il suffit de cliquer sur le lien reçu par e-mail pour revenir à l'enquête.

E. Evidence available for effectiveness of the innovation This component comprises statements related to evidence of the effectiveness of the innovation. The positive impacts of the innovation, whether in terms of improvements in the health of populations, services, or treatments, need to be taken into account. But it is also important to consider possible negative impacts, such as risks and undesirable side effects.

E. Données probantes sur l'efficacité de l'innovation Cette composante rassemble des énoncés liés aux preuves de l'efficacité de l'innovation. Les impacts positifs de l'innovation, qu'il s'agisse d'améliorations dans la santé des populations, dans les services ou des traitements, doivent être prises en compte. Mais il est également important de considérer les éventuels impacts négatifs, tels que les risques et les effets secondaires indésirables.

*Please ensure that you have answered all questions before submitting.

*The meaning of the scales can be found at the end of the page.

*Veuillez vous assurer que vous avez répondu à toutes les questions avant de les soumettre.

*La signification des échelles se trouve en fin de page.

16. There are data on the effectiveness of the innovation. 16. Il existe des données sur l'efficacité de l'innovation.

	1	2	3	4
Is the item important? / L'item est-il important ?	◯	◯	◯	◯
Is the item clear? / L'item est-il clair ?	◯	◯	◯	◯

	1	2	3
Is the item necessary? / L'item est-il nécessaire ?	◯	◯	◯

	1	2	3	4
Is the item important? / L'item est-il important ?	◯	◯	◯	◯
Is the item clear? / L'item est-il clair ?	◯	◯	◯	◯

	1	2	3
Is the item necessary? / L'item est-il nécessaire ?	◯	◯	◯

18. There are data on the disadvantages of the innovation. 18. Il existe des données sur les désavantages de l'innovation.

	1	2	3	4
Is the item important? / L'item est-il important ?	◯	◯	◯	◯
Is the item clear? / L'item est-il clair ?	◯	◯	◯	◯

	1	2	3
Is the item necessary? / L'item est-il nécessaire ?	◯	◯	◯

Please provide any additional comments regarding the relevance, clarity and necessity of the items in this section.

Veuillez fournir tout commentaire supplémentaire concernant la pertinence, la clarté et la nécessité des items de cette section.

La version française suit.

For each scalability statement included in our tool, we are asking you to rate: Is it relevant* considering the aim to scale a health innovation for achieving greater impact?

1 = not relevant

2 = can't assess its relevance unless item is revised, or so much revision necessary that it would no longer be relevant

3 = relevant but needs minor alteration

4 = very relevant and succinct

Is it clear to all potential end users of the tool?

1 = not clear

2 = item needs some revision

3 = clear but needs minor revision

4 = very clear

Is it necessary* to be included in a tool for assessing the scalability of health innovations?

1 = not necessary

2 = useful but not essential

3 = essential

*Please note that while a relevant statement bring an important information, which would be interesting to consider regarding the scalability, a necessary statement express an information that is indispensable to decide about the scaling of a health innovation.

Open-ended (free text) questions

There are open-ended (free text) questions to collect comments and suggestions at the end of each section. You can suggest other assessment items and we ask you to explain why they would be useful. We also encourage you to provide reasons for your decision when you give the item a low score.

NOTE: If you cannot complete the survey in one setting, a "save and continue later" feature is available (button located at the bottom of each survey page). Once you have logged out, to return, simply click on the link received by email to return to the survey.

----Pour chaque énoncés inclus dans notre outil, nous vous demandons d'évaluer : Est-il important* compte tenu de l'objectif de mettre à l'échelle une innovation en santé pour obtenir un plus grand impact ?

1 = non pertinent

2 = impossible d'évaluer la pertinence sans révision de l'item, ou l'item a besoin d'une telle révision qu'il n'est même pas pertinent

3 = pertinent mais nécessite des modifications mineures

4 = très pertinent et succinct

Est-il clair pour tous les potentiel.le.s utilisateur.rice.s de l'outil ?

1 = pas clair

2 = l'item nécessite une certaine révision

3 = clair mais nécessite une révision mineure

4 = très clair

Est-il nécessaire* d'être inclus dans un outil dont l'objectif est d'évaluer le potentiel de mise à l'échelle des innovations en santé ?

1 = non nécessaire

2 = utile mais pas essentiel

3 = essentiel

*Les échelles d'évaluation seront disponibles à la fin de chaque session à titre de rappel.

*Veuillez noter que si un énoncé pertinent apporte une information importante, qu'il serait intéressant de considérer en ce qui concerne la mise à l'échelle, un énoncé nécessaire exprime une information indispensable pour décider sur la mise à l'échelle d'une innovation en santé.

Questions ouvertes (texte libre)

Vous trouverez des questions ouvertes (texte libre) à la fin de chaque section pour collecter vos commentaires et suggestions. Vous pouvez suggérer des autres items d'évaluation du potentiel de mise à l'échelle selon vos perceptions et nous vous demandons de justifier leur inclusion. Nous vous encourageons également à justifier vos décisions pour les scores faibles.

NOTE: Vous pourrez compléter le questionnaire à tout moment, en cliquant sur le lien dans votre courriel, qui vous redirigera automatiquement à l'endroit où vous étiez rendu. Une fois que vous vous êtes déconnecté, pour revenir, il suffit de cliquer sur le lien reçu par e-mail pour revenir à l'enquête.

---

17. The advantages of the innovation and its positive impact on the health of individuals and communities are visible and can be easily demonstrated using evidence-based data. 17. Les avantages de l'innovation et les impacts positifs sur la santé des individus et des communautés sont visibles et facilement démontrés par des données probantes.

F. Scaling costs and quantifiable benefits This component comprises statements related to the costs the innovation scaling. Consideration should be given to whether all the necessary costs can be covered (human resources, financial resources, supplements, and other materials, etc.) and whether they are justifiable in relation to the benefits provided by the innovation (cost-effectiveness). It is important that innovators compare the cost-effectiveness of innovation scaling with the cost-effectiveness of other existing alternatives in order to justify their project as opposed to others.

F. Coûts et avantages quantifiables de la mise à l'échelle Cette composante rassemble des énoncés liés aux coûts de la mise à l'échelle de l'innovation. Il convient d'examiner si l'ensemble des coûts nécessaires sont disponibles (ressources humaines, ressources financières, suppléments et autres matériaux, etc.) et s'ils sont justifiables par rapport aux avantages apportés par l'innovation (coût-efficacité). Il est important que les innovateur.rice.s comparent le coût-efficacité de la mise à l'échelle de l'innovation avec le coût-efficacité d'autres alternatives existantes afin de justifier leur projet par rapport aux autres.

*Please ensure that you have answered all questions before submitting.

*The meaning of the scales can be found at the end of the page.

*Veuillez vous assurer que vous avez répondu à toutes les questions avant de les soumettre.

*La signification des échelles se trouve en fin de page.

19. There are data on financial and human resources [full costs] needed to scaling the innovation. 19. Il existe des données sur les ressources financières et humaines (coûts totaux) nécessaires à la mise à l'échelle de l'innovation.

	1	2	3	4
Is the item important? / L'item est-il important ?	○	○	○	○
Is the item clear? / L'item est-il clair ?	○	○	○	○

	1	2	3
Is the item necessary? / L'item est-il nécessaire ?	○	○	○

20. The innovation requires human and financial resources that can reasonably be expected to be available during the scaling. 20. L'innovation nécessite des ressources humaines et financières dont on peut raisonnablement s'attendre à ce qu'elles soient disponibles pendant la mise à l'échelle.

	1	2	3	4
Is the item important? / L'item est-il important ?	○	○	○	○

Is the item clear? / L'item est-il clair ?	○	○	○	○

	1	2	3
Is the item necessary? / L'item est-il nécessaire ?	○	○	○

21. There are data on the cost-effectiveness of the innovation compared to existing equivalent innovations or alternatives. 21. Il existe des données sur les coût-efficacité de l'innovation (en comparaison aux alternatives existantes).

	1	2	3	4
Is the item important? / L'item est-il important ?	○	○	○	○
Is the item clear? / L'item est-il clair ?	○	○	○	○

	1	2	3
Is the item necessary? / L'item est-il nécessaire ?	○	○	○

Please provide any additional comments regarding the relevance, clarity and necessity of the items in this section.

Veuillez fournir tout commentaire supplémentaire concernant la pertinence, la clarté et la nécessité des items de cette section.

La version française suit.

For each scalability statement included in our tool, we are asking you to rate: Is it relevant* considering the aim to scale a health innovation for achieving greater impact?

1 = not relevant

2 = can't assess its relevance unless item is revised, or so much revision necessary that it would no longer be relevant

3 = relevant but needs minor alteration

4 = very relevant and succinct

Is it clear to all potential end users of the tool?

1 = not clear

2 = item needs some revision

3 = clear but needs minor revision

4 = very clear

Is it necessary* to be included in a tool for assessing the scalability of health innovations?

1 = not necessary

2 = useful but not essential

3 = essential

*Please note that while a relevant statement bring an important information, which would be interesting to consider regarding the scalability, a necessary statement express an information that is indispensable to decide about the scaling of a health innovation.

Open-ended (free text) questions

There are open-ended (free text) questions to collect comments and suggestions at the end of each section. You can suggest other assessment items and we ask you to explain why they would be useful. We also encourage you to provide reasons for your decision when you give the item a low score.

NOTE: If you cannot complete the survey in one setting, a "save and continue later" feature is available (button located at the bottom of each survey page). Once you have logged out, to return, simply click on the link received by email to return to the survey.

----Pour chaque énoncés inclus dans notre outil, nous vous demandons d'évaluer : Est-il important* compte tenu de l'objectif de mettre à l'échelle une innovation en santé pour obtenir un plus grand impact ?

1 = non pertinent

2 = impossible d'évaluer la pertinence sans révision de l'item, ou l'item a besoin d'une telle révision qu'il n'est même pas pertinent

3 = pertinent mais nécessite des modifications mineures

4 = très pertinent et succinct

Est-il clair pour tous les potentiel.le.s utilisateur.rice.s de l'outil ?

1 = pas clair

2 = l'item nécessite une certaine révision

3 = clair mais nécessite une révision mineure

4 = très clair

Est-il nécessaire* d'être inclus dans un outil dont l'objectif est d'évaluer le potentiel de mise à l'échelle des innovations en santé ?

1 = non nécessaire

2 = utile mais pas essentiel

3 = essentiel

*Les échelles d'évaluation seront disponibles à la fin de chaque session à titre de rappel.

*Veuillez noter que si un énoncé pertinent apporte une information importante, qu'il serait intéressant de considérer en ce qui concerne la mise à l'échelle, un énoncé nécessaire exprime une information indispensable pour décider sur la mise à l'échelle d'une innovation en santé.

Questions ouvertes (texte libre)

Vous trouverez des questions ouvertes (texte libre) à la fin de chaque section pour collecter vos commentaires et suggestions. Vous pouvez suggérer des autres items d'évaluation du potentiel de mise à l'échelle selon vos perceptions et nous vous demandons de justifier leur inclusion. Nous vous encourageons également à justifier vos décisions pour les scores faibles.

NOTE: Vous pourrez compléter le questionnaire à tout moment, en cliquant sur le lien dans votre courriel, qui vous redirigera automatiquement à l'endroit où vous étiez rendu. Une fois que vous vous êtes déconnecté, pour revenir, il suffit de cliquer sur le lien reçu par e-mail pour revenir à l'enquête.

G. Implementation fidelity of the innovation This component comprises statements related to the reliability of the innovation during the scaling process. This means considering whether the fundamentals, characteristics, and benefits of the innovation can be maintained. Maintaining reliability means ensuring that the innovation will retain the key aspects of its conception and purpose. Monitoring reliability involves considering the processes and measures that will assess whether the innovation remains reliable during scale-up.

G. Fidélité d'implantation de l'innovation Cette composante rassemble des énoncés liés à la fidélité de l'innovation lors du processus de mise à l'échelle. Cela signifie qu'il faut réfléchir si les bases, les caractéristiques et les bénéficies de l'innovation peuvent être maintenus. Maintenir la fidélité signifie s'assurer que l'innovation conservera les principaux aspects de sa conception et son objectif. Surveiller la fidélité implique de réfléchir sur les processus et les mesures qui permettront d'évaluer si la fidélité de l'innovation est maintenue pendant la mise à l'échelle.

*Please ensure that you have answered all questions before submitting.

*The meaning of the scales can be found at the end of the page.

*Veuillez vous assurer que vous avez répondu à toutes les questions avant de les soumettre.

*La signification des échelles se trouve en fin de page.

22. There are data on implementation fidelity of the innovation. 22. Il existe des données sur la fidélité de l'implantation de l'innovation.

	1	2	3	4
Is the item important? / L'item est-il important ?	○	○	○	○
Is the item clear? / L'item est-il clair ?	○	○	○	○

	1	2	3
Is the item necessary? / L'item est-il nécessaire ?	○	○	○

23. Implementation fidelity of the innovation can be maintained at scale. 23. La fidélité de l'implantation de l'innovation peut être maintenue lorsqu'elle est mise à l'échelle.

	1	2	3	4
Is the item important? / L'item est-il important ?	○	○	○	○

Is the item clear? / L'item est-il clair ?	○	○	○	○

	1	2	3
Is the item necessary? / L'item est-il nécessaire ?	○	○	○

24. Implementation fidelity of the innovation can be monitored at scale. 24. La fidélité de l'implantation de l'innovation peut être surveillée lorsqu'elle est mise à l'échelle.

	1	2	3	4
Is the item important? / L'item est-il important ?	○	○	○	○
Is the item clear? / L'item est-il clair ?	○	○	○	○

	1	2	3
Is the item necessary? / L'item est-il nécessaire ?	○	○	○

Please provide any additional comments regarding the relevance, clarity and necessity of the items in this section.

Veuillez fournir tout commentaire supplémentaire concernant la pertinence, la clarté et la nécessité des items de cette section.

La version française suit.

For each scalability statement included in our tool, we are asking you to rate: Is it relevant* considering the aim to scale a health innovation for achieving greater impact?

1 = not relevant

2 = can't assess its relevance unless item is revised, or so much revision necessary that it would no longer be relevant

3 = relevant but needs minor alteration

4 = very relevant and succinct

Is it clear to all potential end users of the tool?

1 = not clear

2 = item needs some revision

3 = clear but needs minor revision

4 = very clear

Is it necessary* to be included in a tool for assessing the scalability of health innovations?

1 = not necessary

2 = useful but not essential

3 = essential

*Please note that while a relevant statement bring an important information, which would be interesting to consider regarding the scalability, a necessary statement express an information that is indispensable to decide about the scaling of a health innovation.

Open-ended (free text) questions

There are open-ended (free text) questions to collect comments and suggestions at the end of each section. You can suggest other assessment items and we ask you to explain why they would be useful. We also encourage you to provide reasons for your decision when you give the item a low score.

NOTE: If you cannot complete the survey in one setting, a "save and continue later" feature is available (button located at the bottom of each survey page). Once you have logged out, to return, simply click on the link received by email to return to the survey.

----Pour chaque énoncés inclus dans notre outil, nous vous demandons d'évaluer : Est-il important* compte tenu de l'objectif de mettre à l'échelle une innovation en santé pour obtenir un plus grand impact ?

1 = non pertinent

2 = impossible d'évaluer la pertinence sans révision de l'item, ou l'item a besoin d'une telle révision qu'il n'est même pas pertinent

3 = pertinent mais nécessite des modifications mineures

4 = très pertinent et succinct

Est-il clair pour tous les potentiel.le.s utilisateur.rice.s de l'outil ?

1 = pas clair

2 = l'item nécessite une certaine révision

3 = clair mais nécessite une révision mineure

4 = très clair

Est-il nécessaire* d'être inclus dans un outil dont l'objectif est d'évaluer le potentiel de mise à l'échelle des innovations en santé ?

1 = non nécessaire

2 = utile mais pas essentiel

3 = essentiel

*Les échelles d'évaluation seront disponibles à la fin de chaque session à titre de rappel.

*Veuillez noter que si un énoncé pertinent apporte une information importante, qu'il serait intéressant de considérer en ce qui concerne la mise à l'échelle, un énoncé nécessaire exprime une information indispensable pour décider sur la mise à l'échelle d'une innovation en santé.

Questions ouvertes (texte libre)

Vous trouverez des questions ouvertes (texte libre) à la fin de chaque section pour collecter vos commentaires et suggestions. Vous pouvez suggérer des autres items d'évaluation du potentiel de mise à l'échelle selon vos perceptions et nous vous demandons de justifier leur inclusion. Nous vous encourageons également à justifier vos décisions pour les scores faibles.

NOTE: Vous pourrez compléter le questionnaire à tout moment, en cliquant sur le lien dans votre courriel, qui vous redirigera automatiquement à l'endroit où vous étiez rendu. Une fois que vous vous êtes déconnecté, pour revenir, il suffit de cliquer sur le lien reçu par e-mail pour revenir à l'enquête.

H. Adaptability of the innovation This component comprises statements related to the adaptability of the innovation to the scaling context and to the needs of the target population. The innovation must be able to integrate the demands and values of the local population. It is also important to note that there is a tension between the reliability and adaptability components: the adaptations required for innovation scaling must be made without changing its fundamental aspects (fidelity).

H. Adaptabilité de l'innovation Cette composante rassemble des énoncés liés à l'adaptabilité de l'innovation au contexte de mise â l'échelle et aux besoins de la population ciblée. L'innovation doit pouvoir intégrer les demandes et les valeurs de la population locale. Il est aussi important de noter qu'il existe une tension entre les composantes de fidélité et d'adaptabilité : les adaptations nécessaires à la mise à l'échelle de l'innovation doivent être réalisées sans changer ses aspects fondamentaux (fidélité).

*Please ensure that you have answered all questions before submitting.

*The meaning of the scales can be found at the end of the page.

*Veuillez vous assurer que vous avez répondu à toutes les questions avant de les soumettre.

*La signification des échelles se trouve en fin de page.

25. There are data on the adaptability of the innovation. 25. Il existe des données sur l'adaptabilité de l'innovation.

	1	2	3	4
Is the item important? / L'item est-il important ?	○	○	○	○
Is the item clear? / L'item est-il clair ?	○	○	○	○

	1	2	3
Is the item necessary? / L'item est-il nécessaire ?	○	○	○

26. Requirements for local adaptations of the innovation (into a new context) have been considered. 26. Ce qui est nécessaire pour adapter l'innovation localement (nouveau contexte) a été considéré.

	1	2	3	4
Is the item important? / L'item est-il important ?	○	○	○	○

Is the item clear? / L'item est-il clair ?	○	○	○	○

	1	2	3
Is the item necessary? / L'item est-il nécessaire ?	○	○	○

27. The innovation can be (or has been) adapted for scaling without altering its fundamental characteristics, goals and outcomes. 27. Des adaptations peuvent être (ou ont pu être) apportées à l'innovation sans altérer les caractéristiques, les objectifs et les résultats fondamentaux.

	1	2	3	4
Is the item important? / L'item est-il important ?	○	○	○	○
Is the item clear? / L'item est-il clair ?	○	○	○	○

	1	2	3
Is the item necessary? / L'item est-il nécessaire ?	○	○	○

Please provide any additional comments regarding the relevance, clarity and necessity of the items in this section.

Veuillez fournir tout commentaire supplémentaire concernant la pertinence, la clarté et la nécessité des items de cette section.

La version française suit.

For each scalability statement included in our tool, we are asking you to rate: Is it relevant* considering the aim to scale a health innovation for achieving greater impact?

1 = not relevant

2 = can't assess its relevance unless item is revised, or so much revision necessary that it would no longer be relevant

3 = relevant but needs minor alteration

4 = very relevant and succinct

Is it clear to all potential end users of the tool?

1 = not clear

2 = item needs some revision

3 = clear but needs minor revision

4 = very clear

Is it necessary* to be included in a tool for assessing the scalability of health innovations?

1 = not necessary

2 = useful but not essential

3 = essential

*Please note that while a relevant statement bring an important information, which would be interesting to consider regarding the scalability, a necessary statement express an information that is indispensable to decide about the scaling of a health innovation.

Open-ended (free text) questions

There are open-ended (free text) questions to collect comments and suggestions at the end of each section. You can suggest other assessment items and we ask you to explain why they would be useful. We also encourage you to provide reasons for your decision when you give the item a low score.

NOTE: If you cannot complete the survey in one setting, a "save and continue later" feature is available (button located at the bottom of each survey page). Once you have logged out, to return, simply click on the link received by email to return to the survey.

----Pour chaque énoncés inclus dans notre outil, nous vous demandons d'évaluer : Est-il important* compte tenu de l'objectif de mettre à l'échelle une innovation en santé pour obtenir un plus grand impact ?

1 = non pertinent

2 = impossible d'évaluer la pertinence sans révision de l'item, ou l'item a besoin d'une telle révision qu'il n'est même pas pertinent

3 = pertinent mais nécessite des modifications mineures

4 = très pertinent et succinct

Est-il clair pour tous les potentiel.le.s utilisateur.rice.s de l'outil ?

1 = pas clair

2 = l'item nécessite une certaine révision

3 = clair mais nécessite une révision mineure

4 = très clair

Est-il nécessaire* d'être inclus dans un outil dont l'objectif est d'évaluer le potentiel de mise à l'échelle des innovations en santé ?

1 = non nécessaire

2 = utile mais pas essentiel

3 = essentiel

*Les échelles d'évaluation seront disponibles à la fin de chaque session à titre de rappel.

*Veuillez noter que si un énoncé pertinent apporte une information importante, qu'il serait intéressant de considérer en ce qui concerne la mise à l'échelle, un énoncé nécessaire exprime une information indispensable pour décider sur la mise à l'échelle d'une innovation en santé.

Questions ouvertes (texte libre)

Vous trouverez des questions ouvertes (texte libre) à la fin de chaque section pour collecter vos commentaires et suggestions. Vous pouvez suggérer des autres items d'évaluation du potentiel de mise à l'échelle selon vos perceptions et nous vous demandons de justifier leur inclusion. Nous vous encourageons également à justifier vos décisions pour les scores faibles.

NOTE: Vous pourrez compléter le questionnaire à tout moment, en cliquant sur le lien dans votre courriel, qui vous redirigera automatiquement à l'endroit où vous étiez rendu. Une fois que vous vous êtes déconnecté, pour revenir, il suffit de cliquer sur le lien reçu par e-mail pour revenir à l'enquête.

I. Coverage of the innovation This component comprises statements related to the coverage and reach of the innovation scaling. It is about who and how many people or groups (target population) are the beneficiaries. The numerator and denominator of coverage mean, respectively, the number of individual units that were actually covered by the innovation and the total number targeted since its conception.

I. Couverture de l'innovation Cette composante rassemble des énoncés liés à la couverture de la mise à l'échelle de l'innovation. Il s'agit de déterminer qui et combien de personnes ou de groupes (population ciblée) sont les bénéficiaires. Le numérateur et le dénominateur de la couverture signifient, respectivement, le nombre d'unités individuelles qui ont été effectivement couvertes par l'innovation et le nombre total ciblé au début de la conception.

*Please ensure that you have answered all questions before submitting.

*The meaning of the scales can be found at the end of the page.

*Veuillez vous assurer que vous avez répondu à toutes les questions avant de les soumettre.

*La signification des échelles se trouve en fin de page.

28. There is a clear definition of the target population (e.g who will be covered by the scaled innovation and what are their attributes) 28. Il y a une définition claire de qui sont la population ciblée par la mise à l'échelle de l'innovation (c'est-à-dire qui sera couvert.e et quelles sont ses attributs).

	1	2	3	4
Is the item important? / L'item est-il important ?	○	○	○	○
Is the item clear? / L'item est-il clair ?	○	○	○	○

	1	2	3
Is the item necessary? / L'item est-il nécessaire ?	○	○	○

29. There are data on the reach of the innovation among the people involved (numerator & denominator). 29. Il existe des données sur la couverture de l'innovation parmi les personnes concernées (numérateur et dénominateur).

	1	2	3	4
Is the item important? / L'item est-il important ?	○	○	○	○
Is the item clear? / L'item est-il clair ?	○	○	○	○

	1	2	3
Is the item necessary? / L'item est-il nécessaire ?	○	○	○

30. The scaling of the innovation has the potential to reach the intended target population. 30. La mise à l'échelle de l'innovation a le potentiel de couvrir l'ensemble des populations ciblées.

	1	2	3	4
Is the item important? / L'item est-il important ?	○	○	○	○
Is the item clear? / L'item est-il clair ?	○	○	○	○

	1	2	3
Is the item necessary? / L'item est-il nécessaire ?	○	○	○

Please provide any additional comments regarding the relevance, clarity and necessity of the items in this section.

Veuillez fournir tout commentaire supplémentaire concernant la pertinence, la clarté et la nécessité des items de cette section.

La version française suit.

For each scalability statement included in our tool, we are asking you to rate: Is it relevant* considering the aim to scale a health innovation for achieving greater impact?

1 = not relevant

2 = can't assess its relevance unless item is revised, or so much revision necessary that it would no longer be relevant

3 = relevant but needs minor alteration

4 = very relevant and succinct

Is it clear to all potential end users of the tool?

1 = not clear

2 = item needs some revision

3 = clear but needs minor revision

4 = very clear

Is it necessary* to be included in a tool for assessing the scalability of health innovations?

1 = not necessary

2 = useful but not essential

3 = essential

*Please note that while a relevant statement bring an important information, which would be interesting to consider regarding the scalability, a necessary statement express an information that is indispensable to decide about the scaling of a health innovation.

Open-ended (free text) questions

There are open-ended (free text) questions to collect comments and suggestions at the end of each section. You can suggest other assessment items and we ask you to explain why they would be useful. We also encourage you to provide reasons for your decision when you give the item a low score.

NOTE: If you cannot complete the survey in one setting, a "save and continue later" feature is available (button located at the bottom of each survey page). Once you have logged out, to return, simply click on the link received by email to return to the survey.

----Pour chaque énoncés inclus dans notre outil, nous vous demandons d'évaluer : Est-il important* compte tenu de l'objectif de mettre à l'échelle une innovation en santé pour obtenir un plus grand impact ?

1 = non pertinent

2 = impossible d'évaluer la pertinence sans révision de l'item, ou l'item a besoin d'une telle révision qu'il n'est même pas pertinent

3 = pertinent mais nécessite des modifications mineures

4 = très pertinent et succinct

Est-il clair pour tous les potentiel.le.s utilisateur.rice.s de l'outil ?

1 = pas clair

2 = l'item nécessite une certaine révision

3 = clair mais nécessite une révision mineure

4 = très clair

Est-il nécessaire* d'être inclus dans un outil dont l'objectif est d'évaluer le potentiel de mise à l'échelle des innovations en santé ?

1 = non nécessaire

2 = utile mais pas essentiel

3 = essentiel

*Les échelles d'évaluation seront disponibles à la fin de chaque session à titre de rappel.

*Veuillez noter que si un énoncé pertinent apporte une information importante, qu'il serait intéressant de considérer en ce qui concerne la mise à l'échelle, un énoncé nécessaire exprime une information indispensable pour décider sur la mise à l'échelle d'une innovation en santé.

Questions ouvertes (texte libre)

Vous trouverez des questions ouvertes (texte libre) à la fin de chaque section pour collecter vos commentaires et suggestions. Vous pouvez suggérer des autres items d'évaluation du potentiel de mise à l'échelle selon vos perceptions et nous vous demandons de justifier leur inclusion. Nous vous encourageons également à justifier vos décisions pour les scores faibles.

NOTE: Vous pourrez compléter le questionnaire à tout moment, en cliquant sur le lien dans votre courriel, qui vous redirigera automatiquement à l'endroit où vous étiez rendu. Une fois que vous vous êtes déconnecté, pour revenir, il suffit de cliquer sur le lien reçu par e-mail pour revenir à l'enquête.

J. Acceptability of innovation at scale This component comprises statements related to the acceptability of the innovation. It should be well accepted and considered appropriate among stakeholders and the target population.

J. Acceptabilité de l'innovation Cette composante rassemble des énoncés liés à l'acceptabilité de l'innovation. Elle devra être bien acceptée et considérée comme appropriée parmi les parties prenantes et la population ciblée.

*Please ensure that you have answered all questions before submitting.

*The meaning of the scales can be found at the end of the page.

*Veuillez vous assurer que vous avez répondu à toutes les questions avant de les soumettre.

*La signification des échelles se trouve en fin de page.

31. There are data on the adaptability of the innovation. 31. Il existe des données sur l'adaptabilité de l'innovation.

	1	2	3	4
Is the item important? / L'item est-il important ?	○	○	○	○
Is the item clear? / L'item est-il clair ?	○	○	○	○

	1	2	3
L'item est-il nécessaire ?	○	○	○

32. The innovation is presented appropriately using ideas and language that are meaningful to the target populations. 32. L'innovation est présentée de façon appropriée en utilisant un langage significatif pour les populations ciblées.

	1	2	3	4
Is the item important? / L'item est-il important ?	○	○	○	○
Is the item clear? / L'item est-il clair ?	○	○	○	○

	1	2	3
Is the item necessary? / L'item est-il nécessaire ?	○	○	○

Please provide any additional comments regarding the relevance, clarity and necessity of the items in this section.

Veuillez fournir tout commentaire supplémentaire concernant la pertinence, la clarté et la nécessité des items de cette section.

La version française suit.

For each scalability statement included in our tool, we are asking you to rate: Is it relevant* considering the aim to scale a health innovation for achieving greater impact?

1 = not relevant

2 = can't assess its relevance unless item is revised, or so much revision necessary that it would no longer be relevant

3 = relevant but needs minor alteration

4 = very relevant and succinct

Is it clear to all potential end users of the tool?

1 = not clear

2 = item needs some revision

3 = clear but needs minor revision

4 = very clear

Is it necessary* to be included in a tool for assessing the scalability of health innovations?

1 = not necessary

2 = useful but not essential

3 = essential

*Please note that while a relevant statement bring an important information, which would be interesting to consider regarding the scalability, a necessary statement express an information that is indispensable to decide about the scaling of a health innovation.

Open-ended (free text) questions

There are open-ended (free text) questions to collect comments and suggestions at the end of each section. You can suggest other assessment items and we ask you to explain why they would be useful. We also encourage you to provide reasons for your decision when you give the item a low score.

NOTE: If you cannot complete the survey in one setting, a "save and continue later" feature is available (button located at the bottom of each survey page). Once you have logged out, to return, simply click on the link received by email to return to the survey.

----Pour chaque énoncés inclus dans notre outil, nous vous demandons d'évaluer : Est-il important* compte tenu de l'objectif de mettre à l'échelle une innovation en santé pour obtenir un plus grand impact ?

1 = non pertinent

2 = impossible d'évaluer la pertinence sans révision de l'item, ou l'item a besoin d'une telle révision qu'il n'est même pas pertinent

3 = pertinent mais nécessite des modifications mineures

4 = très pertinent et succinct

Est-il clair pour tous les potentiel.le.s utilisateur.rice.s de l'outil ?

1 = pas clair

2 = l'item nécessite une certaine révision

3 = clair mais nécessite une révision mineure

4 = très clair

Est-il nécessaire* d'être inclus dans un outil dont l'objectif est d'évaluer le potentiel de mise à l'échelle des innovations en santé ?

1 = non nécessaire

2 = utile mais pas essentiel

3 = essentiel

*Les échelles d'évaluation seront disponibles à la fin de chaque session à titre de rappel.

*Veuillez noter que si un énoncé pertinent apporte une information importante, qu'il serait intéressant de considérer en ce qui concerne la mise à l'échelle, un énoncé nécessaire exprime une information indispensable pour décider sur la mise à l'échelle d'une innovation en santé.

Questions ouvertes (texte libre)

Vous trouverez des questions ouvertes (texte libre) à la fin de chaque section pour collecter vos commentaires et suggestions. Vous pouvez suggérer des autres items d'évaluation du potentiel de mise à l'échelle selon vos perceptions et nous vous demandons de justifier leur inclusion. Nous vous encourageons également à justifier vos décisions pour les scores faibles.

NOTE: Vous pourrez compléter le questionnaire à tout moment, en cliquant sur le lien dans votre courriel, qui vous redirigera automatiquement à l'endroit où vous étiez rendu. Une fois que vous vous êtes déconnecté, pour revenir, il suffit de cliquer sur le lien reçu par e-mail pour revenir à l'enquête.

K. Adoption of innovation at scale This component comprises statements related to the adoption of the innovation. It is important to consider how many ordinary people, professionals, or service units will use or even integrate the innovation as a practice. The numerator and denominator, respectively, reflect the number of units (people, services, etc.) that have adopted the innovation and the total number targeted since its conception.

K. Adoption de l'innovation à grande échelle Cette composante rassemble des énoncés liés à l'adoption de l'innovation. Il est important de considérer combien de personnes, de professionnels ou d'unités de service utiliseront ou même intégreront l'innovation en tant que pratique. Le numérateur et le dénominateur, respectivement, reflètent le nombre d'unités (personnes, services, etc.) qui ont adopté l'innovation et le nombre total ciblé depuis sa conception.

*Please ensure that you have answered all questions before submitting.

*The meaning of the scales can be found at the end of the page.

*Veuillez vous assurer que vous avez répondu à toutes les questions avant de les soumettre.

*La signification des échelles se trouve en fin de page.

33. There are data on the adoption of the innovation among the target population (numerator & denominator). 33. Il existe des données sur l'adoption de l'innovation parmi les populations ciblées (numérateur et dénominateur).

	1	2	3	4
Is the item important? / L'item est-il important ?	○	○	○	○
Is the item clear? / L'item est-il clair ?	○	○	○	○

	1	2	3
Is the item necessary? / L'item est-il nécessaire ?	○	○	○

34. There are data on target population intention to adopt the innovation. 34. Il existe des données sur l'intention des populations ciblées d'adopter l'innovation.

	1	2	3	4
Is the item important? / L'item est-il important ?	○	○	○	○
Is the item clear? / L'item est-il clair ?	○	○	○	○

	1	2	3
Is the item necessary? / L'item est-il nécessaire ?	◯	◯	◯

Please provide any additional comments regarding the relevance, clarity and necessity of the items in this section.

Veuillez fournir tout commentaire supplémentaire concernant la pertinence, la clarté et la nécessité des items de cette section.

La version française suit.

For each scalability statement included in our tool, we are asking you to rate: Is it relevant* considering the aim to scale a health innovation for achieving greater impact?

1 = not relevant

2 = can't assess its relevance unless item is revised, or so much revision necessary that it would no longer be relevant

3 = relevant but needs minor alteration

4 = very relevant and succinct

Is it clear to all potential end users of the tool?

1 = not clear

2 = item needs some revision

3 = clear but needs minor revision

4 = very clear

Is it necessary* to be included in a tool for assessing the scalability of health innovations?

1 = not necessary

2 = useful but not essential

3 = essential

*Please note that while a relevant statement bring an important information, which would be interesting to consider regarding the scalability, a necessary statement express an information that is indispensable to decide about the scaling of a health innovation.

Open-ended (free text) questions

There are open-ended (free text) questions to collect comments and suggestions at the end of each section. You can suggest other assessment items and we ask you to explain why they would be useful. We also encourage you to provide reasons for your decision when you give the item a low score.

NOTE: If you cannot complete the survey in one setting, a "save and continue later" feature is available (button located at the bottom of each survey page). Once you have logged out, to return, simply click on the link received by email to return to the survey.

----Pour chaque énoncés inclus dans notre outil, nous vous demandons d'évaluer : Est-il important* compte tenu de l'objectif de mettre à l'échelle une innovation en santé pour obtenir un plus grand impact ?

1 = non pertinent

2 = impossible d'évaluer la pertinence sans révision de l'item, ou l'item a besoin d'une telle révision qu'il n'est même pas pertinent

3 = pertinent mais nécessite des modifications mineures

4 = très pertinent et succinct

Est-il clair pour tous les potentiel.le.s utilisateur.rice.s de l'outil ?

1 = pas clair

2 = l'item nécessite une certaine révision

3 = clair mais nécessite une révision mineure

4 = très clair

Est-il nécessaire* d'être inclus dans un outil dont l'objectif est d'évaluer le potentiel de mise à l'échelle des innovations en santé ?

1 = non nécessaire

2 = utile mais pas essentiel

3 = essentiel

*Les échelles d'évaluation seront disponibles à la fin de chaque session à titre de rappel.

*Veuillez noter que si un énoncé pertinent apporte une information importante, qu'il serait intéressant de considérer en ce qui concerne la mise à l'échelle, un énoncé nécessaire exprime une information indispensable pour décider sur la mise à l'échelle d'une innovation en santé.

Questions ouvertes (texte libre)

Vous trouverez des questions ouvertes (texte libre) à la fin de chaque section pour collecter vos commentaires et suggestions. Vous pouvez suggérer des autres items d'évaluation du potentiel de mise à l'échelle selon vos perceptions et nous vous demandons de justifier leur inclusion. Nous vous encourageons également à justifier vos décisions pour les scores faibles.

NOTE: Vous pourrez compléter le questionnaire à tout moment, en cliquant sur le lien dans votre courriel, qui vous redirigera automatiquement à l'endroit où vous étiez rendu. Une fois que vous vous êtes déconnecté, pour revenir, il suffit de cliquer sur le lien reçu par e-mail pour revenir à l'enquête.

L. Scaling environment This component comprises statements related to the setting in which the innovation will be scaled. It is necessary to consider whether the context in which the innovation will be disseminated is sufficiently similar or compatible with the pilot context from which evidence of the innovation's impact was gathered. In addition, key stakeholders must be present locally for scaling.

L. Milieu de mise à l'échelle Cette composante rassemble des énoncés liés au milieu de mise à l'échelle de l'innovation. Il est nécessaire d'examiner si le contexte dans lequel l'innovation sera diffusée est suffisamment similaire ou compatible avec le contexte pilote à partir duquel les preuves de l'impact de l'innovation ont été recueillies. En outre, il est nécessaire que les principaux acteurs soient présents localement pour la mise à l'échelle.

*Please ensure that you have answered all questions before submitting.

*The meaning of the scales can be found at the end of the page.

*Veuillez vous assurer que vous avez répondu à toutes les questions avant de les soumettre.

*La signification des échelles se trouve en fin de page.

35. The innovation has been tested in the type of environment in which it is to be scaled up. 35. L'innovation a été testée dans le même type de milieu où elle sera mise à l'échelle.

	1	2	3	4
Is the item important? / L'item est-il important ?	○	○	○	○
Is the item clear? / L'item est-il clair ?	○	○	○	○

	1	2	3
Is the item necessary? / L'item est-il nécessaire ?	○	○	○

36. Local multi-stakeholder partnerships have been established to support the scaling. 36. Les partenariats multipartites locaux sont en place pour soutenir la mise à l'échelle.

	1	2	3	4
Is the item important? / L'item est-il important ?	○	○	○	○
Is the item clear? / L'item est-il clair ?	○	○	○	○

	1	2	3
Is the item necessary? / L'item est-il nécessaire ?	○	○	○

37. Appropriately trained personnel exists and is available for scaling [in target sites]. 37. Il existe une main-d'œuvre formée de façon appropriée et elle est disponible dans le milieu où l'innovation sera mise à l'échelle.

	1	2	3	4
Is the item important? / L'item est-il important ?	○	○	○	○
Is the item clear? / L'item est-il clair ?	○	○	○	○

	1	2	3
Is the item necessary? / L'item est-il nécessaire ?	○	○	○

Please provide any additional comments regarding the relevance, clarity and necessity of the items in this section.

Veuillez fournir tout commentaire supplémentaire concernant la pertinence, la clarté et la nécessité des items de cette section.

La version française suit.

For each scalability statement included in our tool, we are asking you to rate: Is it relevant* considering the aim to scale a health innovation for achieving greater impact?

1 = not relevant

2 = can't assess its relevance unless item is revised, or so much revision necessary that it would no longer be relevant

3 = relevant but needs minor alteration

4 = very relevant and succinct

Is it clear to all potential end users of the tool?

1 = not clear

2 = item needs some revision

3 = clear but needs minor revision

4 = very clear

Is it necessary* to be included in a tool for assessing the scalability of health innovations?

1 = not necessary

2 = useful but not essential

3 = essential

*Please note that while a relevant statement bring an important information, which would be interesting to consider regarding the scalability, a necessary statement express an information that is indispensable to decide about the scaling of a health innovation.

Open-ended (free text) questions

There are open-ended (free text) questions to collect comments and suggestions at the end of each section. You can suggest other assessment items and we ask you to explain why they would be useful. We also encourage you to provide reasons for your decision when you give the item a low score.

NOTE: If you cannot complete the survey in one setting, a "save and continue later" feature is available (button located at the bottom of each survey page). Once you have logged out, to return, simply click on the link received by email to return to the survey.

----Pour chaque énoncés inclus dans notre outil, nous vous demandons d'évaluer : Est-il important* compte tenu de l'objectif de mettre à l'échelle une innovation en santé pour obtenir un plus grand impact ?

1 = non pertinent

2 = impossible d'évaluer la pertinence sans révision de l'item, ou l'item a besoin d'une telle révision qu'il n'est même pas pertinent

3 = pertinent mais nécessite des modifications mineures

4 = très pertinent et succinct

Est-il clair pour tous les potentiel.le.s utilisateur.rice.s de l'outil ?

1 = pas clair

2 = l'item nécessite une certaine révision

3 = clair mais nécessite une révision mineure

4 = très clair

Est-il nécessaire* d'être inclus dans un outil dont l'objectif est d'évaluer le potentiel de mise à l'échelle des innovations en santé ?

1 = non nécessaire

2 = utile mais pas essentiel

3 = essentiel

*Les échelles d'évaluation seront disponibles à la fin de chaque session à titre de rappel.

*Veuillez noter que si un énoncé pertinent apporte une information importante, qu'il serait intéressant de considérer en ce qui concerne la mise à l'échelle, un énoncé nécessaire exprime une information indispensable pour décider sur la mise à l'échelle d'une innovation en santé.

Questions ouvertes (texte libre)

Vous trouverez des questions ouvertes (texte libre) à la fin de chaque section pour collecter vos commentaires et suggestions. Vous pouvez suggérer des autres items d'évaluation du potentiel de mise à l'échelle selon vos perceptions et nous vous demandons de justifier leur inclusion. Nous vous encourageons également à justifier vos décisions pour les scores faibles.

NOTE: Vous pourrez compléter le questionnaire à tout moment, en cliquant sur le lien dans votre courriel, qui vous redirigera automatiquement à l'endroit où vous étiez rendu. Une fois que vous vous êtes déconnecté, pour revenir, il suffit de cliquer sur le lien reçu par e-mail pour revenir à l'enquête.

M. Infrastructure required for scaling This component comprises statements related to the infrastructure required for innovation scaling. Evidence must be gathered concerning the feasibility of the innovation in relation to the infrastructure needed for its realisation. For scaling to be successful, it is important for the organisational infrastructure to be available throughout the process, including monitoring and assessment structures (professionals, processes, measures, and the like) and a skilled workforce.

M. Infrastructure requise pour la mise à l'échelle Cette composante rassemble des énoncés liés à l'infrastructure requise pour la mise à l'échelle de l'innovation. Il est nécessaire de rassembler des preuves de la faisabilité de l'innovation par rapport à l'infrastructure nécessaire à sa réalisation. Pour une mise à l'échelle réussie, il est important que l'infrastructure organisationnelle soit disponible tout au long du processus, y compris la main-d'œuvre qualifiée et les structures de suivi et d'évaluation (professionnels, processus, mesures, etc.).

*Please ensure that you have answered all questions before submitting.

*The meaning of the scales can be found at the end of the page.

*Veuillez vous assurer que vous avez répondu à toutes les questions avant de les soumettre.

*La signification des échelles se trouve en fin de page.

38. There are data on the feasibility of the innovation. 38. Il existe des données sur la faisabilité de l'innovation.

	1	2	3	4
Is the item important? / L'item est-il important ?	○	○	○	○
Is the item clear? / L'item est-il clair ?	○	○	○	○

	1	2	3
Is the item necessary? / L'item est-il nécessaire ?	○	○	○

39. Infrastructure requirements for scaling the innovation are achievable. 39. Les exigences d'infrastructure pour la mise à l'échelle de l'innovation sont réalisables.

	1	2	3	4
Is the item important? / L'item est-il important ?	○	○	○	○

Is the item clear? / L'item est-il clair ?	○	○	○	○

	1	2	3
Is the item necessary? / L'item est-il nécessaire ?	○	○	○

40. The organizational infrastructure required is available for scaling the innovation. 40. L'infrastructure organisationnelle requise est disponible pour la mise à l'échelle de l'innovation.

	1	2	3	4
Is the item important? / L'item est-il important ?	○	○	○	○
Is the item clear? / L'item est-il clair ?	○	○	○	○

	1	2	3
Is the item necessary? / L'item est-il nécessaire ?	○	○	○

41. Structures are in place for monitoring the scaling process. 41. Des structures sont en place pour surveiller le processus de mise à l'échelle.

	1	2	3	4
Is the item important? / L'item est-il important ?	○	○	○	○
Is the item clear? / L'item est-il clair ?	○	○	○	○

	1	2	3
Is the item necessary? / L'item est-il nécessaire ?	○	○	○

42. Structures are in place for evaluating the scaling process. 42. Des structures sont en place pour évaluer le processus de mise à l'échelle.

	1	2	3	4
Is the item important? / L'item est-il important ?	○	○	○	○
Is the item clear? / L'item est-il clair ?	○	○	○	○

	1	2	3
Is the item necessary? / L'item est-il nécessaire ?	○	○	○

Please provide any additional comments regarding the relevance, clarity and necessity of the items in this section.

Veuillez fournir tout commentaire supplémentaire concernant la pertinence, la clarté et la nécessité des items de cette section.

La version française suit.

For each scalability statement included in our tool, we are asking you to rate: Is it relevant* considering the aim to scale a health innovation for achieving greater impact?

1 = not relevant

2 = can't assess its relevance unless item is revised, or so much revision necessary that it would no longer be relevant

3 = relevant but needs minor alteration

4 = very relevant and succinct

Is it clear to all potential end users of the tool?

1 = not clear

2 = item needs some revision

3 = clear but needs minor revision

4 = very clear

Is it necessary* to be included in a tool for assessing the scalability of health innovations?

1 = not necessary

2 = useful but not essential

3 = essential

*Please note that while a relevant statement bring an important information, which would be interesting to consider regarding the scalability, a necessary statement express an information that is indispensable to decide about the scaling of a health innovation.

Open-ended (free text) questions

There are open-ended (free text) questions to collect comments and suggestions at the end of each section. You can suggest other assessment items and we ask you to explain why they would be useful. We also encourage you to provide reasons for your decision when you give the item a low score.

NOTE: If you cannot complete the survey in one setting, a "save and continue later" feature is available (button located at the bottom of each survey page). Once you have logged out, to return, simply click on the link received by email to return to the survey.

----Pour chaque énoncés inclus dans notre outil, nous vous demandons d'évaluer : Est-il important* compte tenu de l'objectif de mettre à l'échelle une innovation en santé pour obtenir un plus grand impact ?

1 = non pertinent

2 = impossible d'évaluer la pertinence sans révision de l'item, ou l'item a besoin d'une telle révision qu'il n'est même pas pertinent

3 = pertinent mais nécessite des modifications mineures

4 = très pertinent et succinct

Est-il clair pour tous les potentiel.le.s utilisateur.rice.s de l'outil ?

1 = pas clair

2 = l'item nécessite une certaine révision

3 = clair mais nécessite une révision mineure

4 = très clair

Est-il nécessaire* d'être inclus dans un outil dont l'objectif est d'évaluer le potentiel de mise à l'échelle des innovations en santé ?

1 = non nécessaire

2 = utile mais pas essentiel

3 = essentiel

*Les échelles d'évaluation seront disponibles à la fin de chaque session à titre de rappel.

*Veuillez noter que si un énoncé pertinent apporte une information importante, qu'il serait intéressant de considérer en ce qui concerne la mise à l'échelle, un énoncé nécessaire exprime une information indispensable pour décider sur la mise à l'échelle d'une innovation en santé.

Questions ouvertes (texte libre)

Vous trouverez des questions ouvertes (texte libre) à la fin de chaque section pour collecter vos commentaires et suggestions. Vous pouvez suggérer des autres items d'évaluation du potentiel de mise à l'échelle selon vos perceptions et nous vous demandons de justifier leur inclusion. Nous vous encourageons également à justifier vos décisions pour les scores faibles.

NOTE: Vous pourrez compléter le questionnaire à tout moment, en cliquant sur le lien dans votre courriel, qui vous redirigera automatiquement à l'endroit où vous étiez rendu. Une fois que vous vous êtes déconnecté, pour revenir, il suffit de cliquer sur le lien reçu par e-mail pour revenir à l'enquête.

N. Sustainability This component comprises statements related to the sustainability of the innovation and involves considering the availability of human and material resources over time and how long they will be available.

N. Pérennisation Cette composante rassemble des énoncés liés à la pérennisation de l'innovation. Cela implique de réfléchir à la disponibilité des ressources humaines et matérielles dans le temps et à la durée pendant lesquelles elles seront disponibles.

*Please ensure that you have answered all questions before submitting.

*The meaning of the scales can be found at the end of the page.

*Veuillez vous assurer que vous avez répondu à toutes les questions avant de les soumettre.

*La signification des échelles se trouve en fin de page.

43. The sustainability (maintaining the scaling on a lasting basis) has been considered. 43. La pérennisation de la mise à l'échelle a été considérée.

	1	2	3	4
Is the item important? / L'item est-il important ?	○	○	○	○
Is the item clear? / L'item est-il clair ?	○	○	○	○

	1	2	3
Is the item necessary? / L'item est-il nécessaire ?	○	○	○

44. The human and financial resources required for scaling are sustainable. 44. Les ressources humaines et financières requises pour la mise à l'échelle de l'innovation sont pérennes.

	1	2	3	4
Is the item important? / L'item est-il important ?	○	○	○	○
Is the item clear? / L'item est-il clair ?	○	○	○	○

	1	2	3
Is the item necessary? / L'item est-il nécessaire ?	○	○	○

45. There are data on how long the innovation can it be sustained at scale. 45. Il existe des données sur la durée de la pérennité de l'innovation à grande échelle.

	1	2	3	4
Is the item important? / L'item est-il important ?	◯	◯	◯	◯
Is the item clear? / L'item est-il clair ?	◯	◯	◯	◯

	1	2	3
Is the item necessary? / L'item est-il nécessaire ?	◯	◯	◯

Please provide any additional comments regarding the relevance, clarity and necessity of the items in this section.

Veuillez fournir tout commentaire supplémentaire concernant la pertinence, la clarté et la nécessité des items de cette section.

La version française suit.

For each scalability statement included in our tool, we are asking you to rate: Is it relevant* considering the aim to scale a health innovation for achieving greater impact?

1 = not relevant

2 = can't assess its relevance unless item is revised, or so much revision necessary that it would no longer be relevant

3 = relevant but needs minor alteration

4 = very relevant and succinct

Is it clear to all potential end users of the tool?

1 = not clear

2 = item needs some revision

3 = clear but needs minor revision

4 = very clear

Is it necessary* to be included in a tool for assessing the scalability of health innovations?

1 = not necessary

2 = useful but not essential

3 = essential

*Please note that while a relevant statement bring an important information, which would be interesting to consider regarding the scalability, a necessary statement express an information that is indispensable to decide about the scaling of a health innovation.

Open-ended (free text) questions

There are open-ended (free text) questions to collect comments and suggestions at the end of each section. You can suggest other assessment items and we ask you to explain why they would be useful. We also encourage you to provide reasons for your decision when you give the item a low score.

NOTE: If you cannot complete the survey in one setting, a "save and continue later" feature is available (button located at the bottom of each survey page). Once you have logged out, to return, simply click on the link received by email to return to the survey.

----Pour chaque énoncés inclus dans notre outil, nous vous demandons d'évaluer : Est-il important* compte tenu de l'objectif de mettre à l'échelle une innovation en santé pour obtenir un plus grand impact ?

1 = non pertinent

2 = impossible d'évaluer la pertinence sans révision de l'item, ou l'item a besoin d'une telle révision qu'il n'est même pas pertinent

3 = pertinent mais nécessite des modifications mineures

4 = très pertinent et succinct

Est-il clair pour tous les potentiel.le.s utilisateur.rice.s de l'outil ?1 = pas clair

2 = l'item nécessite une certaine révision

3 = clair mais nécessite une révision mineure

4 = très clair

Est-il nécessaire* d'être inclus dans un outil dont l'objectif est d'évaluer le potentiel de mise à l'échelle des innovations en santé ?

1 = non nécessaire

2 = utile mais pas essentiel

3 = essentiel

*Les échelles d'évaluation seront disponibles à la fin de chaque session à titre de rappel.

*Veuillez noter que si un énoncé pertinent apporte une information importante, qu'il serait intéressant de considérer en ce qui concerne la mise à l'échelle, un énoncé nécessaire exprime une information indispensable pour décider sur la mise à l'échelle d'une innovation en santé.

Questions ouvertes (texte libre)

Vous trouverez des questions ouvertes (texte libre) à la fin de chaque section pour collecter vos commentaires et suggestions. Vous pouvez suggérer des autres items d'évaluation du potentiel de mise à l'échelle selon vos perceptions et nous vous demandons de justifier leur inclusion. Nous vous encourageons également à justifier vos décisions pour les scores faibles.

NOTE: Vous pourrez compléter le questionnaire à tout moment, en cliquant sur le lien dans votre courriel, qui vous redirigera automatiquement à l'endroit où vous étiez rendu. Une fois que vous vous êtes déconnecté, pour revenir, il suffit de cliquer sur le lien reçu par e-mail pour revenir à l'enquête.

User involvement in developing and validating an instrument for assessing the scalability of innovations in health and social services: a consensus process using the Delphi method. Corôa et al. 2024

Additional File 4

A. Heath Problem This component comprises statements related to the health problem that the scaling of the innovation addresses. In this context, it is important to take into account the perceptions of stakeholders and the target population.

A. Problème de santé Cette composante rassemble des énoncés liés au problème de santé auquel s'adresse la mise à l'échelle de l'innovation. Dans ce cadre, il est important de prendre en compte les perceptions des parties prenantes et de la population ciblée.

*Please ensure that you have answered all questions before submitting.

*The meaning of the scales can be found at the end of the page.

*Veuillez vous assurer que vous avez répondu à toutes les questions avant de les soumettre.

*La signification des échelles se trouve en fin de page.

1. The innovation addresses a relevant health problem. 1. Cette innovation répond à un problème en santé pertinent.

	1	2	3	4
Is the item important? / L'item est-il important ?	○	○	○	○
Is the item clear? / L'item est-il clair ?	○	○	○	○

	1	2	3
Is the item necessary? / L'item est-il nécessaire ?	○	○	○

2. Key stakeholders have explicitly requested scaling of this innovation. 2. Il y a une demande explicite de mise à l'échelle de l'innovation par les parties prenantes.

	1	2	3	4
Is the item important? / L'item est-il important ?	○	○	○	○
Is the item clear? / L'item est-il clair ?	○	○	○	○

	1	2	3
Is the item necessary? / L'item est-il nécessaire ?	○	○	○

3. Target populations have explicitly requested scaling of this innovation. 3. Il y a une demande explicite de mise à l'échelle de l'innovation par les populations ciblées.

	1	2	3	4
Is the item important? / L'item est-il important ?	○	○	○	○
Is the item clear? / L'item est-il clair ?	○	○	○	○

	1	2	3
Is the item necessary? / L'item est-il nécessaire ?	○	○	○

Please provide any additional comments regarding the relevance, clarity and necessity of the items in this section.

Veuillez fournir tout commentaire supplémentaire concernant la pertinence, la clarté et la nécessité des items de cette section.

La version française suit.

For each scalability statement included in our tool, we are asking you to rate: Is it relevant* considering the aim to scale a health innovation for achieving greater impact?

1 = not relevant

2 = can't assess its relevance unless item is revised, or so much revision necessary that it would no longer be relevant

3 = relevant but needs minor alteration

4 = very relevant and succinct

Is it clear to all potential end users of the tool?

1 = not clear

2 = item needs some revision

3 = clear but needs minor revision

4 = very clear

Is it necessary* to be included in a tool for assessing the scalability of health innovations?

1 = not necessary

2 = useful but not essential

3 = essential

*Please note that while a relevant statement bring an important information, which would be interesting to consider regarding the scalability, a necessary statement express an information that is indispensable to decide about the scaling of a health innovation.

Open-ended (free text) questions

There are open-ended (free text) questions to collect comments and suggestions at the end of each section. You can suggest other assessment items and we ask you to explain why they would be useful. We also encourage you to provide reasons for your decision when you give the item a low score.

NOTE: If you cannot complete the survey in one setting, a "save and continue later" feature is available (button located at the bottom of each survey page). Once you have logged out, to return, simply click on the link received by email to return to the survey.

----Pour chaque énoncés inclus dans notre outil, nous vous demandons d'évaluer : Est-il important* compte tenu de l'objectif de mettre à l'échelle une innovation en santé pour obtenir un plus grand impact ?

1 = non pertinent

2 = impossible d'évaluer la pertinence sans révision de l'item, ou l'item a besoin d'une telle révision qu'il n'est même pas pertinent

3 = pertinent mais nécessite des modifications mineures

4 = très pertinent et succinct

Est-il clair pour tous les potentiel.le.s utilisateur.rice.s de l'outil ?

1 = pas clair

2 = l'item nécessite une certaine révision

3 = clair mais nécessite une révision mineure

4 = très clair

Est-il nécessaire* d'être inclus dans un outil dont l'objectif est d'évaluer le potentiel de mise à l'échelle des innovations en santé ?

1 = non nécessaire

2 = utile mais pas essentiel

3 = essentiel

*Les échelles d'évaluation seront disponibles à la fin de chaque session à titre de rappel.

*Veuillez noter que si un énoncé pertinent apporte une information importante, qu'il serait intéressant de considérer en ce qui concerne la mise à l'échelle, un énoncé nécessaire exprime une information indispensable pour décider sur la mise à l'échelle d'une innovation en santé.

Questions ouvertes (texte libre)

Vous trouverez des questions ouvertes (texte libre) à la fin de chaque section pour collecter vos commentaires et suggestions. Vous pouvez suggérer des autres items d'évaluation du potentiel de mise à l'échelle selon vos perceptions et nous vous demandons de justifier leur inclusion. Nous vous encourageons également à justifier vos décisions pour les scores faibles.

NOTE: Vous pourrez compléter le questionnaire à tout moment, en cliquant sur le lien dans votre courriel, qui vous redirigera automatiquement à l'endroit où vous étiez rendu. Une fois que vous vous êtes déconnecté, pour revenir, il suffit de cliquer sur le lien reçu par e-mail pour revenir à l'enquête.

B. Scaling development This component comprises statements related to the innovation scaling development process. This development needs to be systematic and to involve stakeholders and the target population.

B. Développement de la mise à l'échelle Cette composante rassemble des énoncés liés au processus de développement de la mise à l'échelle de l'innovation. Ce développement doit être systématique et il doit impliquer les parties prenantes et la population ciblée.

*Please ensure that you have answered all questions before submitting.

*The meaning of the scales can be found at the end of the page.

*Veuillez vous assurer que vous avez répondu à toutes les questions avant de les soumettre.

*La signification des échelles se trouve en fin de page.

4. The development of the scaling is informed by a theory, a model or framework. 4. Une théorie, un modèle ou un cadre conceptuel informent la mise à l'échelle.

	1	2	3	4
Is the item important? / L'item est-il important ?	○	○	○	○
Is the item clear? / L'item est-il clair ?	○	○	○	○

	1	2	3
Is the item necessary? / L'item est-il nécessaire ?	○	○	○

5. Stakeholders have given their feedback on the scaling. 5. Les parties prenantes ont donné leur avis sur la mise à l'échelle.

	1	2	3	4
Is the item important? / L'item est-il important ?	○	○	○	○
Is the item clear? / L'item est-il clair ?	○	○	○	○

	1	2	3
Is the item necessary? / L'item est-il nécessaire ?	○	○	○

6. Target populations have given feedback on the scaling. 6. Les populations ciblées ont donné leur avis sur la mise à l'échelle.

	1	2	3	4
Is the item important? / L'item est-il important ?	○	○	○	○
Is the item clear? / L'item est-il clair ?	○	○	○	○

	1	2	3
Is the item necessary? / L'item est-il nécessaire ?	○	○	○

Please provide any additional comments regarding the relevance, clarity and necessity of the items in this section.

Veuillez fournir tout commentaire supplémentaire concernant la pertinence, la clarté et la nécessité des items de cette section.

La version française suit.

For each scalability statement included in our tool, we are asking you to rate: Is it relevant* considering the aim to scale a health innovation for achieving greater impact?

1 = not relevant

2 = can't assess its relevance unless item is revised, or so much revision necessary that it would no longer be relevant

3 = relevant but needs minor alteration

4 = very relevant and succinct

Is it clear to all potential end users of the tool?

1 = not clear

2 = item needs some revision

3 = clear but needs minor revision

4 = very clear

Is it necessary* to be included in a tool for assessing the scalability of health innovations?

1 = not necessary

2 = useful but not essential

3 = essential

*Please note that while a relevant statement bring an important information, which would be interesting to consider regarding the scalability, a necessary statement express an information that is indispensable to decide about the scaling of a health innovation.

Open-ended (free text) questions

There are open-ended (free text) questions to collect comments and suggestions at the end of each section. You can suggest other assessment items and we ask you to explain why they would be useful. We also encourage you to provide reasons for your decision when you give the item a low score.

NOTE: If you cannot complete the survey in one setting, a "save and continue later" feature is available (button located at the bottom of each survey page). Once you have logged out, to return, simply click on the link received by email to return to the survey.

----Pour chaque énoncés inclus dans notre outil, nous vous demandons d'évaluer : Est-il important* compte tenu de l'objectif de mettre à l'échelle une innovation en santé pour obtenir un plus grand impact ?

1 = non pertinent

2 = impossible d'évaluer la pertinence sans révision de l'item, ou l'item a besoin d'une telle révision qu'il n'est même pas pertinent

3 = pertinent mais nécessite des modifications mineures

4 = très pertinent et succinct

Est-il clair pour tous les potentiel.le.s utilisateur.rice.s de l'outil ?

1 = pas clair

2 = l'item nécessite une certaine révision

3 = clair mais nécessite une révision mineure

4 = très clair

Est-il nécessaire* d'être inclus dans un outil dont l'objectif est d'évaluer le potentiel de mise à l'échelle des innovations en santé ?

1 = non nécessaire

2 = utile mais pas essentiel

3 = essentiel

*Les échelles d'évaluation seront disponibles à la fin de chaque session à titre de rappel.

*Veuillez noter que si un énoncé pertinent apporte une information importante, qu'il serait intéressant de considérer en ce qui concerne la mise à l'échelle, un énoncé nécessaire exprime une information indispensable pour décider sur la mise à l'échelle d'une innovation en santé.

Questions ouvertes (texte libre)

Vous trouverez des questions ouvertes (texte libre) à la fin de chaque section pour collecter vos commentaires et suggestions. Vous pouvez suggérer des autres items d'évaluation du potentiel de mise à l'échelle selon vos perceptions et nous vous demandons de justifier leur inclusion. Nous vous encourageons également à justifier vos décisions pour les scores faibles.

NOTE: Vous pourrez compléter le questionnaire à tout moment, en cliquant sur le lien dans votre courriel, qui vous redirigera automatiquement à l'endroit où vous étiez rendu. Une fois que vous vous êtes déconnecté, pour revenir, il suffit de cliquer sur le lien reçu par e-mail pour revenir à l'enquête.

C. Characteristics of the innovation This component comprises statements related to the characteristics of the innovation scaling that aim to ensure its integration into existing health services. The component also aims to ensure its understanding by stakeholders and the target population. These questions address ethical aspects and social norms in the context of scaling.

C. Caractéristiques de l'innovation Cette composante rassemble des énoncés liés aux caractéristiques de la mise à l'échelle de l'innovation qui visent à assurer son intégration dans les services de santé existants. Elle vise aussi à assurer sa compréhension par les parties prenantes et par la population ciblée. Ces questions portent sur les aspects éthiques et les normes sociales dans le contexte de mise à échelle.

*Please ensure that you have answered all questions before submitting.

*The meaning of the scales can be found at the end of the page.

*Veuillez vous assurer que vous avez répondu à toutes les questions avant de les soumettre.

*La signification des échelles se trouve en fin de page.

7. The stakeholders concerned share a common vision of what is to be scaled up (its goal). 7. Les parties prenantes impliquées partagent une vision commune sur ce qui signifie la mise à l'échelle (son but).

	1	2	3	4
Is the item important? / L'item est-il important ?	○	○	○	○
Is the item clear? / L'item est-il clair ?	○	○	○	○

	1	2	3
Is the item necessary? / L'item est-il nécessaire ?	○	○	○

8. The intervention ensures continuity of care in a wide range of services. 8. L'innovation assure la continuité des soins dans une vaste gamme de services.

	1	2	3	4
Is the item important? / L'item est-il important ?	○	○	○	○
Is the item clear? / L'item est-il clair ?	○	○	○	○

	1	2	3
Is the item necessary? / L'item est-il nécessaire ?	○	○	○

9. The innovation is simple and easy to understand for the target population. 9. L'innovation est facile à comprendre pour les populations ciblées.

	1	2	3	4
Is the item important? / L'item est-il important ?	○	○	○	○
Is the item clear? / L'item est-il clair ?	○	○	○	○

	1	2	3
Is the item necessary? / L'item est-il nécessaire ?	○	○	○

10. The innovation is sex- and gender-sensitive. 10. L'innovation tient compte des questions de sexe et de genre.

	1	2	3	4
Is the item important? / L'item est-il important ?	○	○	○	○
Is the item clear? / L'item est-il clair ?	○	○	○	○

	1	2	3
Is the item necessary? / L'item est-il nécessaire ?	○	○	○

11. The innovation respects indigenous communities, visible minority and their culture[s]. 11. L'innovation respecte les communautés autochtones et les minorités visibles et leur[s] culture[s].

	1	2	3	4
Is the item important? / L'item est-il important ?	○	○	○	○
Is the item clear? / L'item est-il clair ?	○	○	○	○

	1	2	3
Is the item necessary? / L'item est-il nécessaire ?	○	○	○

Please provide any additional comments regarding the relevance, clarity and necessity of the items in this section.

Veuillez fournir tout commentaire supplémentaire concernant la pertinence, la clarté et la nécessité des items de cette section.

La version française suit.

For each scalability statement included in our tool, we are asking you to rate: Is it relevant* considering the aim to scale a health innovation for achieving greater impact?

1 = not relevant

2 = can't assess its relevance unless item is revised, or so much revision necessary that it would no longer be relevant

3 = relevant but needs minor alteration

4 = very relevant and succinct

Is it clear to all potential end users of the tool?

1 = not clear

2 = item needs some revision

3 = clear but needs minor revision

4 = very clear

Is it necessary* to be included in a tool for assessing the scalability of health innovations?

1 = not necessary

2 = useful but not essential

3 = essential

*Please note that while a relevant statement bring an important information, which would be interesting to consider regarding the scalability, a necessary statement express an information that is indispensable to decide about the scaling of a health innovation.

Open-ended (free text) questions

There are open-ended (free text) questions to collect comments and suggestions at the end of each section. You can suggest other assessment items and we ask you to explain why they would be useful. We also encourage you to provide reasons for your decision when you give the item a low score.

NOTE: If you cannot complete the survey in one setting, a "save and continue later" feature is available (button located at the bottom of each survey page). Once you have logged out, to return, simply click on the link received by email to return to the survey.

----Pour chaque énoncés inclus dans notre outil, nous vous demandons d'évaluer : Est-il important* compte tenu de l'objectif de mettre à l'échelle une innovation en santé pour obtenir un plus grand impact ?

1 = non pertinent

2 = impossible d'évaluer la pertinence sans révision de l'item, ou l'item a besoin d'une telle révision qu'il n'est même pas pertinent

3 = pertinent mais nécessite des modifications mineures

4 = très pertinent et succinct

Est-il clair pour tous les potentiel.le.s utilisateur.rice.s de l'outil ?

1 = pas clair

2 = l'item nécessite une certaine révision

3 = clair mais nécessite une révision mineure

4 = très clair

Est-il nécessaire* d'être inclus dans un outil dont l'objectif est d'évaluer le potentiel de mise à l'échelle des innovations en santé ?

1 = non nécessaire

2 = utile mais pas essentiel

3 = essentiel

*Les échelles d'évaluation seront disponibles à la fin de chaque session à titre de rappel.

*Veuillez noter que si un énoncé pertinent apporte une information importante, qu'il serait intéressant de considérer en ce qui concerne la mise à l'échelle, un énoncé nécessaire exprime une information indispensable pour décider sur la mise à l'échelle d'une innovation en santé.

Questions ouvertes (texte libre)

Vous trouverez des questions ouvertes (texte libre) à la fin de chaque section pour collecter vos commentaires et suggestions. Vous pouvez suggérer des autres items d'évaluation du potentiel de mise à l'échelle selon vos perceptions et nous vous demandons de justifier leur inclusion. Nous vous encourageons également à justifier vos décisions pour les scores faibles.

NOTE: Vous pourrez compléter le questionnaire à tout moment, en cliquant sur le lien dans votre courriel, qui vous redirigera automatiquement à l'endroit où vous étiez rendu. Une fois que vous vous êtes déconnecté, pour revenir, il suffit de cliquer sur le lien reçu par e-mail pour revenir à l'enquête.

D. Strategic, political or environmental context of the scaling This component comprises statements related to existing health policies, both at the broader national level and in the specific context of the innovation. It also invites innovators to reflect on possible existing policy barriers that may reduce scalability.

D. Contexte stratégique, politique et environnemental de la mise à l'échelle Cette composante rassemble des énoncés liés aux politiques de santé existantes, que ce soit au niveau national plus large ou dans le contexte spécifique de l'innovation. De même, il invite les innovateur.rice.s à réfléchir aux éventuels obstacles politiques existants qui peuvent diminuer le potentiel de mise à l'échelle.

*Please ensure that you have answered all questions before submitting.

*The meaning of the scales can be found at the end of the page.

*Veuillez vous assurer que vous avez répondu à toutes les questions avant de les soumettre.

*La signification des échelles se trouve en fin de page.

12. The innovation is consistent with existing national health policies, plans and priorities. 12. L'innovation est conforme aux politiques, plans et priorités de santé du gouvernement.

	1	2	3	4
Is the item important? / L'item est-il important ?	○	○	○	○
Is the item clear? / L'item est-il clair ?	○	○	○	○

	1	2	3
Is the item necessary? / L'item est-il nécessaire ?	○	○	○

13. The innovation addresses needs in government health programs. 13. L'innovation répond aux besoins des programmes de santé du gouvernement.

	1	2	3	4
Is the item important? / L'item est-il important ?	○	○	○	○
Is the item clear? / L'item est-il clair ?	○	○	○	○

	1	2	3
Is the item necessary? / L'item est-il nécessaire ?	○	○	○

14. The innovation complies with policy guidelines in the target scale-up setting. 14. L'innovation est conforme aux directives politiques du milieu dans lequel elle sera mise à l'échelle.

	1	2	3	4
Is the item important? / L'item est-il important ?	◯	◯	◯	◯
Is the item clear? / L'item est-il clair ?	◯	◯	◯	◯

	1	2	3
Is the item necessary? / L'item est-il nécessaire ?	◯	◯	◯

15. There are no political obstacles to the scaling of this innovation. 15. Il n'y a pas d'obstacle politique à la mise à l'échelle de cette innovation.

	1	2	3	4
Is the item important? / L'item est-il important ?	◯	◯	◯	◯
Is the item clear? / L'item est-il clair ?	◯	◯	◯	◯

	1	2	3
Is the item necessary? / L'item est-il nécessaire ?	◯	◯	◯

Please provide any additional comments regarding the relevance, clarity and necessity of the items in this section.

Veuillez fournir tout commentaire supplémentaire concernant la pertinence, la clarté et la nécessité des items de cette section.

La version française suit.

For each scalability statement included in our tool, we are asking you to rate: Is it relevant* considering the aim to scale a health innovation for achieving greater impact?

1 = not relevant

2 = can't assess its relevance unless item is revised, or so much revision necessary that it would no longer be relevant

3 = relevant but needs minor alteration

4 = very relevant and succinct

Is it clear to all potential end users of the tool?

1 = not clear

2 = item needs some revision

3 = clear but needs minor revision

4 = very clear

Is it necessary* to be included in a tool for assessing the scalability of health innovations?

1 = not necessary

2 = useful but not essential

3 = essential

*Please note that while a relevant statement bring an important information, which would be interesting to consider regarding the scalability, a necessary statement express an information that is indispensable to decide about the scaling of a health innovation.

Open-ended (free text) questions

There are open-ended (free text) questions to collect comments and suggestions at the end of each section. You can suggest other assessment items and we ask you to explain why they would be useful. We also encourage you to provide reasons for your decision when you give the item a low score.

NOTE: If you cannot complete the survey in one setting, a "save and continue later" feature is available (button located at the bottom of each survey page). Once you have logged out, to return, simply click on the link received by email to return to the survey.

----Pour chaque énoncés inclus dans notre outil, nous vous demandons d'évaluer : Est-il important* compte tenu de l'objectif de mettre à l'échelle une innovation en santé pour obtenir un plus grand impact ?

1 = non pertinent

2 = impossible d'évaluer la pertinence sans révision de l'item, ou l'item a besoin d'une telle révision qu'il n'est même pas pertinent

3 = pertinent mais nécessite des modifications mineures

4 = très pertinent et succinct

Est-il clair pour tous les potentiel.le.s utilisateur.rice.s de l'outil ?1 = pas clair

2 = l'item nécessite une certaine révision

3 = clair mais nécessite une révision mineure

4 = très clair

Est-il nécessaire* d'être inclus dans un outil dont l'objectif est d'évaluer le potentiel de mise à l'échelle des innovations en santé ?

1 = non nécessaire

2 = utile mais pas essentiel

3 = essentiel

*Les échelles d'évaluation seront disponibles à la fin de chaque session à titre de rappel.

*Veuillez noter que si un énoncé pertinent apporte une information importante, qu'il serait intéressant de considérer en ce qui concerne la mise à l'échelle, un énoncé nécessaire exprime une information indispensable pour décider sur la mise à l'échelle d'une innovation en santé.

Questions ouvertes (texte libre)

Vous trouverez des questions ouvertes (texte libre) à la fin de chaque section pour collecter vos commentaires et suggestions. Vous pouvez suggérer des autres items d'évaluation du potentiel de mise à l'échelle selon vos perceptions et nous vous demandons de justifier leur inclusion. Nous vous encourageons également à justifier vos décisions pour les scores faibles.

NOTE: Vous pourrez compléter le questionnaire à tout moment, en cliquant sur le lien dans votre courriel, qui vous redirigera automatiquement à l'endroit où vous étiez rendu. Une fois que vous vous êtes déconnecté, pour revenir, il suffit de cliquer sur le lien reçu par e-mail pour revenir à l'enquête.

E. Evidence available for effectiveness of the innovation This component comprises statements related to evidence of the effectiveness of the innovation. The positive impacts of the innovation, whether in terms of improvements in the health of populations, services, or treatments, need to be taken into account. But it is also important to consider possible negative impacts, such as risks and undesirable side effects.

E. Données probantes sur l'efficacité de l'innovation Cette composante rassemble des énoncés liés aux preuves de l'efficacité de l'innovation. Les impacts positifs de l'innovation, qu'il s'agisse d'améliorations dans la santé des populations, dans les services ou des traitements, doivent être prises en compte. Mais il est également important de considérer les éventuels impacts négatifs, tels que les risques et les effets secondaires indésirables.

*Please ensure that you have answered all questions before submitting.

*The meaning of the scales can be found at the end of the page.

*Veuillez vous assurer que vous avez répondu à toutes les questions avant de les soumettre.

*La signification des échelles se trouve en fin de page.

16. There are data on the effectiveness of the innovation. 16. Il existe des données sur l'efficacité de l'innovation.

	1	2	3	4
Is the item important? / L'item est-il important ?	○	○	○	○
Is the item clear? / L'item est-il clair ?	○	○	○	○

	1	2	3
Is the item necessary? / L'item est-il nécessaire ?	○	○	○

	1	2	3	4
Is the item important? / L'item est-il important ?	○	○	○	○
Is the item clear? / L'item est-il clair ?	○	○	○	○

	1	2	3
Is the item necessary? / L'item est-il nécessaire ?	○	○	○

18. There are data on the disadvantages of the innovation. 18. Il existe des données sur les désavantages de l'innovation.

	1	2	3	4
Is the item important? / L'item est-il important ?	◯	◯	◯	◯
Is the item clear? / L'item est-il clair ?	◯	◯	◯	◯

	1	2	3
Is the item necessary? / L'item est-il nécessaire ?	◯	◯	◯

Please provide any additional comments regarding the relevance, clarity and necessity of the items in this section.

Veuillez fournir tout commentaire supplémentaire concernant la pertinence, la clarté et la nécessité des items de cette section.

La version française suit.

For each scalability statement included in our tool, we are asking you to rate: Is it relevant* considering the aim to scale a health innovation for achieving greater impact?

1 = not relevant

2 = can't assess its relevance unless item is revised, or so much revision necessary that it would no longer be relevant

3 = relevant but needs minor alteration

4 = very relevant and succinct

Is it clear to all potential end users of the tool?

1 = not clear

2 = item needs some revision

3 = clear but needs minor revision

4 = very clear

Is it necessary* to be included in a tool for assessing the scalability of health innovations?

1 = not necessary

2 = useful but not essential

3 = essential

*Please note that while a relevant statement bring an important information, which would be interesting to consider regarding the scalability, a necessary statement express an information that is indispensable to decide about the scaling of a health innovation.

Open-ended (free text) questions

There are open-ended (free text) questions to collect comments and suggestions at the end of each section. You can suggest other assessment items and we ask you to explain why they would be useful. We also encourage you to provide reasons for your decision when you give the item a low score.

NOTE: If you cannot complete the survey in one setting, a "save and continue later" feature is available (button located at the bottom of each survey page). Once you have logged out, to return, simply click on the link received by email to return to the survey.

----Pour chaque énoncés inclus dans notre outil, nous vous demandons d'évaluer : Est-il important* compte tenu de l'objectif de mettre à l'échelle une innovation en santé pour obtenir un plus grand impact ?

1 = non pertinent

2 = impossible d'évaluer la pertinence sans révision de l'item, ou l'item a besoin d'une telle révision qu'il n'est même pas pertinent

3 = pertinent mais nécessite des modifications mineures

4 = très pertinent et succinct

Est-il clair pour tous les potentiel.le.s utilisateur.rice.s de l'outil ?

1 = pas clair

2 = l'item nécessite une certaine révision

3 = clair mais nécessite une révision mineure

4 = très clair

Est-il nécessaire* d'être inclus dans un outil dont l'objectif est d'évaluer le potentiel de mise à l'échelle des innovations en santé ?

1 = non nécessaire

2 = utile mais pas essentiel

3 = essentiel

*Les échelles d'évaluation seront disponibles à la fin de chaque session à titre de rappel.

*Veuillez noter que si un énoncé pertinent apporte une information importante, qu'il serait intéressant de considérer en ce qui concerne la mise à l'échelle, un énoncé nécessaire exprime une information indispensable pour décider sur la mise à l'échelle d'une innovation en santé.

Questions ouvertes (texte libre)

Vous trouverez des questions ouvertes (texte libre) à la fin de chaque section pour collecter vos commentaires et suggestions. Vous pouvez suggérer des autres items d'évaluation du potentiel de mise à l'échelle selon vos perceptions et nous vous demandons de justifier leur inclusion. Nous vous encourageons également à justifier vos décisions pour les scores faibles.

NOTE: Vous pourrez compléter le questionnaire à tout moment, en cliquant sur le lien dans votre courriel, qui vous redirigera automatiquement à l'endroit où vous étiez rendu. Une fois que vous vous êtes déconnecté, pour revenir, il suffit de cliquer sur le lien reçu par e-mail pour revenir à l'enquête.

---

17. The advantages of the innovation and its positive impact on the health of individuals and communities are visible and can be easily demonstrated using evidence-based data. 17. Les avantages de l'innovation et les impacts positifs sur la santé des individus et des communautés sont visibles et facilement démontrés par des données probantes.

F. Scaling costs and quantifiable benefits This component comprises statements related to the costs the innovation scaling. Consideration should be given to whether all the necessary costs can be covered (human resources, financial resources, supplements, and other materials, etc.) and whether they are justifiable in relation to the benefits provided by the innovation (cost-effectiveness). It is important that innovators compare the cost-effectiveness of innovation scaling with the cost-effectiveness of other existing alternatives in order to justify their project as opposed to others.

F. Coûts et avantages quantifiables de la mise à l'échelle Cette composante rassemble des énoncés liés aux coûts de la mise à l'échelle de l'innovation. Il convient d'examiner si l'ensemble des coûts nécessaires sont disponibles (ressources humaines, ressources financières, suppléments et autres matériaux, etc.) et s'ils sont justifiables par rapport aux avantages apportés par l'innovation (coût-efficacité). Il est important que les innovateur.rice.s comparent le coût-efficacité de la mise à l'échelle de l'innovation avec le coût-efficacité d'autres alternatives existantes afin de justifier leur projet par rapport aux autres.

*Please ensure that you have answered all questions before submitting.

*The meaning of the scales can be found at the end of the page.

*Veuillez vous assurer que vous avez répondu à toutes les questions avant de les soumettre.

*La signification des échelles se trouve en fin de page.

19. There are data on financial and human resources [full costs] needed to scaling the innovation. 19. Il existe des données sur les ressources financières et humaines (coûts totaux) nécessaires à la mise à l'échelle de l'innovation.

	1	2	3	4
Is the item important? / L'item est-il important ?	○	○	○	○
Is the item clear? / L'item est-il clair ?	○	○	○	○

	1	2	3
Is the item necessary? / L'item est-il nécessaire ?	○	○	○

20. The innovation requires human and financial resources that can reasonably be expected to be available during the scaling. 20. L'innovation nécessite des ressources humaines et financières dont on peut raisonnablement s'attendre à ce qu'elles soient disponibles pendant la mise à l'échelle.

	1	2	3	4
Is the item important? / L'item est-il important ?	○	○	○	○

Is the item clear? / L'item est-il clair ?	○	○	○	○

	1	2	3
Is the item necessary? / L'item est-il nécessaire ?	○	○	○

21. There are data on the cost-effectiveness of the innovation compared to existing equivalent innovations or alternatives. 21. Il existe des données sur les coût-efficacité de l'innovation (en comparaison aux alternatives existantes).

	1	2	3	4
Is the item important? / L'item est-il important ?	○	○	○	○
Is the item clear? / L'item est-il clair ?	○	○	○	○

	1	2	3
Is the item necessary? / L'item est-il nécessaire ?	○	○	○

Please provide any additional comments regarding the relevance, clarity and necessity of the items in this section.

Veuillez fournir tout commentaire supplémentaire concernant la pertinence, la clarté et la nécessité des items de cette section.

La version française suit.

For each scalability statement included in our tool, we are asking you to rate: Is it relevant* considering the aim to scale a health innovation for achieving greater impact?

1 = not relevant

2 = can't assess its relevance unless item is revised, or so much revision necessary that it would no longer be relevant

3 = relevant but needs minor alteration

4 = very relevant and succinct

Is it clear to all potential end users of the tool?

1 = not clear

2 = item needs some revision

3 = clear but needs minor revision

4 = very clear

Is it necessary* to be included in a tool for assessing the scalability of health innovations?

1 = not necessary

2 = useful but not essential

3 = essential

*Please note that while a relevant statement bring an important information, which would be interesting to consider regarding the scalability, a necessary statement express an information that is indispensable to decide about the scaling of a health innovation.

Open-ended (free text) questions

There are open-ended (free text) questions to collect comments and suggestions at the end of each section. You can suggest other assessment items and we ask you to explain why they would be useful. We also encourage you to provide reasons for your decision when you give the item a low score.

NOTE: If you cannot complete the survey in one setting, a "save and continue later" feature is available (button located at the bottom of each survey page). Once you have logged out, to return, simply click on the link received by email to return to the survey.

----Pour chaque énoncés inclus dans notre outil, nous vous demandons d'évaluer : Est-il important* compte tenu de l'objectif de mettre à l'échelle une innovation en santé pour obtenir un plus grand impact ?

1 = non pertinent

2 = impossible d'évaluer la pertinence sans révision de l'item, ou l'item a besoin d'une telle révision qu'il n'est même pas pertinent

3 = pertinent mais nécessite des modifications mineures

4 = très pertinent et succinct

Est-il clair pour tous les potentiel.le.s utilisateur.rice.s de l'outil ?

1 = pas clair

2 = l'item nécessite une certaine révision

3 = clair mais nécessite une révision mineure

4 = très clair

Est-il nécessaire* d'être inclus dans un outil dont l'objectif est d'évaluer le potentiel de mise à l'échelle des innovations en santé ?

1 = non nécessaire

2 = utile mais pas essentiel

3 = essentiel

*Les échelles d'évaluation seront disponibles à la fin de chaque session à titre de rappel.

*Veuillez noter que si un énoncé pertinent apporte une information importante, qu'il serait intéressant de considérer en ce qui concerne la mise à l'échelle, un énoncé nécessaire exprime une information indispensable pour décider sur la mise à l'échelle d'une innovation en santé.

Questions ouvertes (texte libre)

Vous trouverez des questions ouvertes (texte libre) à la fin de chaque section pour collecter vos commentaires et suggestions. Vous pouvez suggérer des autres items d'évaluation du potentiel de mise à l'échelle selon vos perceptions et nous vous demandons de justifier leur inclusion. Nous vous encourageons également à justifier vos décisions pour les scores faibles.

NOTE: Vous pourrez compléter le questionnaire à tout moment, en cliquant sur le lien dans votre courriel, qui vous redirigera automatiquement à l'endroit où vous étiez rendu. Une fois que vous vous êtes déconnecté, pour revenir, il suffit de cliquer sur le lien reçu par e-mail pour revenir à l'enquête.

G. Implementation fidelity of the innovation This component comprises statements related to the reliability of the innovation during the scaling process. This means considering whether the fundamentals, characteristics, and benefits of the innovation can be maintained. Maintaining reliability means ensuring that the innovation will retain the key aspects of its conception and purpose. Monitoring reliability involves considering the processes and measures that will assess whether the innovation remains reliable during scale-up.

G. Fidélité d'implantation de l'innovation Cette composante rassemble des énoncés liés à la fidélité de l'innovation lors du processus de mise à l'échelle. Cela signifie qu'il faut réfléchir si les bases, les caractéristiques et les bénéficies de l'innovation peuvent être maintenus. Maintenir la fidélité signifie s'assurer que l'innovation conservera les principaux aspects de sa conception et son objectif. Surveiller la fidélité implique de réfléchir sur les processus et les mesures qui permettront d'évaluer si la fidélité de l'innovation est maintenue pendant la mise à l'échelle.

*Please ensure that you have answered all questions before submitting.

*The meaning of the scales can be found at the end of the page.

*Veuillez vous assurer que vous avez répondu à toutes les questions avant de les soumettre.

*La signification des échelles se trouve en fin de page.

22. There are data on implementation fidelity of the innovation. 22. Il existe des données sur la fidélité de l'implantation de l'innovation.

	1	2	3	4
Is the item important? / L'item est-il important ?	○	○	○	○
Is the item clear? / L'item est-il clair ?	○	○	○	○

	1	2	3
Is the item necessary? / L'item est-il nécessaire ?	○	○	○

23. Implementation fidelity of the innovation can be maintained at scale. 23. La fidélité de l'implantation de l'innovation peut être maintenue lorsqu'elle est mise à l'échelle.

	1	2	3	4
Is the item important? / L'item est-il important ?	○	○	○	○

Is the item clear? / L'item est-il clair ?	○	○	○	○

	1	2	3
Is the item necessary? / L'item est-il nécessaire ?	○	○	○

24. Implementation fidelity of the innovation can be monitored at scale. 24. La fidélité de l'implantation de l'innovation peut être surveillée lorsqu'elle est mise à l'échelle.

	1	2	3	4
Is the item important? / L'item est-il important ?	○	○	○	○
Is the item clear? / L'item est-il clair ?	○	○	○	○

	1	2	3
Is the item necessary? / L'item est-il nécessaire ?	○	○	○

Please provide any additional comments regarding the relevance, clarity and necessity of the items in this section.

Veuillez fournir tout commentaire supplémentaire concernant la pertinence, la clarté et la nécessité des items de cette section.

La version française suit.

For each scalability statement included in our tool, we are asking you to rate: Is it relevant* considering the aim to scale a health innovation for achieving greater impact?

1 = not relevant

2 = can't assess its relevance unless item is revised, or so much revision necessary that it would no longer be relevant

3 = relevant but needs minor alteration

4 = very relevant and succinct

Is it clear to all potential end users of the tool?

1 = not clear

2 = item needs some revision

3 = clear but needs minor revision

4 = very clear

Is it necessary* to be included in a tool for assessing the scalability of health innovations?

1 = not necessary

2 = useful but not essential

3 = essential

*Please note that while a relevant statement bring an important information, which would be interesting to consider regarding the scalability, a necessary statement express an information that is indispensable to decide about the scaling of a health innovation.

Open-ended (free text) questions

There are open-ended (free text) questions to collect comments and suggestions at the end of each section. You can suggest other assessment items and we ask you to explain why they would be useful. We also encourage you to provide reasons for your decision when you give the item a low score.

NOTE: If you cannot complete the survey in one setting, a "save and continue later" feature is available (button located at the bottom of each survey page). Once you have logged out, to return, simply click on the link received by email to return to the survey.

----Pour chaque énoncés inclus dans notre outil, nous vous demandons d'évaluer : Est-il important* compte tenu de l'objectif de mettre à l'échelle une innovation en santé pour obtenir un plus grand impact ?

1 = non pertinent

2 = impossible d'évaluer la pertinence sans révision de l'item, ou l'item a besoin d'une telle révision qu'il n'est même pas pertinent

3 = pertinent mais nécessite des modifications mineures

4 = très pertinent et succinct

Est-il clair pour tous les potentiel.le.s utilisateur.rice.s de l'outil ?

1 = pas clair

2 = l'item nécessite une certaine révision

3 = clair mais nécessite une révision mineure

4 = très clair

Est-il nécessaire* d'être inclus dans un outil dont l'objectif est d'évaluer le potentiel de mise à l'échelle des innovations en santé ?

1 = non nécessaire

2 = utile mais pas essentiel

3 = essentiel

*Les échelles d'évaluation seront disponibles à la fin de chaque session à titre de rappel.

*Veuillez noter que si un énoncé pertinent apporte une information importante, qu'il serait intéressant de considérer en ce qui concerne la mise à l'échelle, un énoncé nécessaire exprime une information indispensable pour décider sur la mise à l'échelle d'une innovation en santé.

Questions ouvertes (texte libre)

Vous trouverez des questions ouvertes (texte libre) à la fin de chaque section pour collecter vos commentaires et suggestions. Vous pouvez suggérer des autres items d'évaluation du potentiel de mise à l'échelle selon vos perceptions et nous vous demandons de justifier leur inclusion. Nous vous encourageons également à justifier vos décisions pour les scores faibles.

NOTE: Vous pourrez compléter le questionnaire à tout moment, en cliquant sur le lien dans votre courriel, qui vous redirigera automatiquement à l'endroit où vous étiez rendu. Une fois que vous vous êtes déconnecté, pour revenir, il suffit de cliquer sur le lien reçu par e-mail pour revenir à l'enquête.

H. Adaptability of the innovation This component comprises statements related to the adaptability of the innovation to the scaling context and to the needs of the target population. The innovation must be able to integrate the demands and values of the local population. It is also important to note that there is a tension between the reliability and adaptability components: the adaptations required for innovation scaling must be made without changing its fundamental aspects (fidelity).

H. Adaptabilité de l'innovation Cette composante rassemble des énoncés liés à l'adaptabilité de l'innovation au contexte de mise â l'échelle et aux besoins de la population ciblée. L'innovation doit pouvoir intégrer les demandes et les valeurs de la population locale. Il est aussi important de noter qu'il existe une tension entre les composantes de fidélité et d'adaptabilité : les adaptations nécessaires à la mise à l'échelle de l'innovation doivent être réalisées sans changer ses aspects fondamentaux (fidélité).

*Please ensure that you have answered all questions before submitting.

*The meaning of the scales can be found at the end of the page.

*Veuillez vous assurer que vous avez répondu à toutes les questions avant de les soumettre.

*La signification des échelles se trouve en fin de page.

25. There are data on the adaptability of the innovation. 25. Il existe des données sur l'adaptabilité de l'innovation.

	1	2	3	4
Is the item important? / L'item est-il important ?	○	○	○	○
Is the item clear? / L'item est-il clair ?	○	○	○	○

	1	2	3
Is the item necessary? / L'item est-il nécessaire ?	○	○	○

26. Requirements for local adaptations of the innovation (into a new context) have been considered. 26. Ce qui est nécessaire pour adapter l'innovation localement (nouveau contexte) a été considéré.

	1	2	3	4
Is the item important? / L'item est-il important ?	○	○	○	○

Is the item clear? / L'item est-il clair ?	○	○	○	○

	1	2	3
Is the item necessary? / L'item est-il nécessaire ?	○	○	○

27. The innovation can be (or has been) adapted for scaling without altering its fundamental characteristics, goals and outcomes. 27. Des adaptations peuvent être (ou ont pu être) apportées à l'innovation sans altérer les caractéristiques, les objectifs et les résultats fondamentaux.

	1	2	3	4
Is the item important? / L'item est-il important ?	○	○	○	○
Is the item clear? / L'item est-il clair ?	○	○	○	○

	1	2	3
Is the item necessary? / L'item est-il nécessaire ?	○	○	○

Please provide any additional comments regarding the relevance, clarity and necessity of the items in this section.

Veuillez fournir tout commentaire supplémentaire concernant la pertinence, la clarté et la nécessité des items de cette section.

La version française suit.

For each scalability statement included in our tool, we are asking you to rate: Is it relevant* considering the aim to scale a health innovation for achieving greater impact?

1 = not relevant

2 = can't assess its relevance unless item is revised, or so much revision necessary that it would no longer be relevant

3 = relevant but needs minor alteration

4 = very relevant and succinct

Is it clear to all potential end users of the tool?

1 = not clear

2 = item needs some revision

3 = clear but needs minor revision

4 = very clear

Is it necessary* to be included in a tool for assessing the scalability of health innovations?

1 = not necessary

2 = useful but not essential

3 = essential

*Please note that while a relevant statement bring an important information, which would be interesting to consider regarding the scalability, a necessary statement express an information that is indispensable to decide about the scaling of a health innovation.

Open-ended (free text) questions

There are open-ended (free text) questions to collect comments and suggestions at the end of each section. You can suggest other assessment items and we ask you to explain why they would be useful. We also encourage you to provide reasons for your decision when you give the item a low score.

NOTE: If you cannot complete the survey in one setting, a "save and continue later" feature is available (button located at the bottom of each survey page). Once you have logged out, to return, simply click on the link received by email to return to the survey.

----Pour chaque énoncés inclus dans notre outil, nous vous demandons d'évaluer : Est-il important* compte tenu de l'objectif de mettre à l'échelle une innovation en santé pour obtenir un plus grand impact ?

1 = non pertinent

2 = impossible d'évaluer la pertinence sans révision de l'item, ou l'item a besoin d'une telle révision qu'il n'est même pas pertinent

3 = pertinent mais nécessite des modifications mineures

4 = très pertinent et succinct

Est-il clair pour tous les potentiel.le.s utilisateur.rice.s de l'outil ?

1 = pas clair

2 = l'item nécessite une certaine révision

3 = clair mais nécessite une révision mineure

4 = très clair

Est-il nécessaire* d'être inclus dans un outil dont l'objectif est d'évaluer le potentiel de mise à l'échelle des innovations en santé ?

1 = non nécessaire

2 = utile mais pas essentiel

3 = essentiel

*Les échelles d'évaluation seront disponibles à la fin de chaque session à titre de rappel.

*Veuillez noter que si un énoncé pertinent apporte une information importante, qu'il serait intéressant de considérer en ce qui concerne la mise à l'échelle, un énoncé nécessaire exprime une information indispensable pour décider sur la mise à l'échelle d'une innovation en santé.

Questions ouvertes (texte libre)

Vous trouverez des questions ouvertes (texte libre) à la fin de chaque section pour collecter vos commentaires et suggestions. Vous pouvez suggérer des autres items d'évaluation du potentiel de mise à l'échelle selon vos perceptions et nous vous demandons de justifier leur inclusion. Nous vous encourageons également à justifier vos décisions pour les scores faibles.

NOTE: Vous pourrez compléter le questionnaire à tout moment, en cliquant sur le lien dans votre courriel, qui vous redirigera automatiquement à l'endroit où vous étiez rendu. Une fois que vous vous êtes déconnecté, pour revenir, il suffit de cliquer sur le lien reçu par e-mail pour revenir à l'enquête.

I. Coverage of the innovation This component comprises statements related to the coverage and reach of the innovation scaling. It is about who and how many people or groups (target population) are the beneficiaries. The numerator and denominator of coverage mean, respectively, the number of individual units that were actually covered by the innovation and the total number targeted since its conception.

I. Couverture de l'innovation Cette composante rassemble des énoncés liés à la couverture de la mise à l'échelle de l'innovation. Il s'agit de déterminer qui et combien de personnes ou de groupes (population ciblée) sont les bénéficiaires. Le numérateur et le dénominateur de la couverture signifient, respectivement, le nombre d'unités individuelles qui ont été effectivement couvertes par l'innovation et le nombre total ciblé au début de la conception.

*Please ensure that you have answered all questions before submitting.

*The meaning of the scales can be found at the end of the page.

*Veuillez vous assurer que vous avez répondu à toutes les questions avant de les soumettre.

*La signification des échelles se trouve en fin de page.

28. There is a clear definition of the target population (e.g who will be covered by the scaled innovation and what are their attributes) 28. Il y a une définition claire de qui sont la population ciblée par la mise à l'échelle de l'innovation (c'est-à-dire qui sera couvert.e et quelles sont ses attributs).

	1	2	3	4
Is the item important? / L'item est-il important ?	○	○	○	○
Is the item clear? / L'item est-il clair ?	○	○	○	○

	1	2	3
Is the item necessary? / L'item est-il nécessaire ?	○	○	○

29. There are data on the reach of the innovation among the people involved (numerator & denominator). 29. Il existe des données sur la couverture de l'innovation parmi les personnes concernées (numérateur et dénominateur).

	1	2	3	4
Is the item important? / L'item est-il important ?	○	○	○	○
Is the item clear? / L'item est-il clair ?	○	○	○	○

	1	2	3
Is the item necessary? / L'item est-il nécessaire ?	◯	◯	◯

30. The scaling of the innovation has the potential to reach the intended target population. 30. La mise à l'échelle de l'innovation a le potentiel de couvrir l'ensemble des populations ciblées.

	1	2	3	4
Is the item important? / L'item est-il important ?	◯	◯	◯	◯
Is the item clear? / L'item est-il clair ?	◯	◯	◯	◯

	1	2	3
Is the item necessary? / L'item est-il nécessaire ?	◯	◯	◯

Please provide any additional comments regarding the relevance, clarity and necessity of the items in this section.

Veuillez fournir tout commentaire supplémentaire concernant la pertinence, la clarté et la nécessité des items de cette section.

La version française suit.

For each scalability statement included in our tool, we are asking you to rate: Is it relevant* considering the aim to scale a health innovation for achieving greater impact?

1 = not relevant

2 = can't assess its relevance unless item is revised, or so much revision necessary that it would no longer be relevant

3 = relevant but needs minor alteration

4 = very relevant and succinct

Is it clear to all potential end users of the tool?

1 = not clear

2 = item needs some revision

3 = clear but needs minor revision

4 = very clear

Is it necessary* to be included in a tool for assessing the scalability of health innovations?

1 = not necessary

2 = useful but not essential

3 = essential

*Please note that while a relevant statement bring an important information, which would be interesting to consider regarding the scalability, a necessary statement express an information that is indispensable to decide about the scaling of a health innovation.

Open-ended (free text) questions

There are open-ended (free text) questions to collect comments and suggestions at the end of each section. You can suggest other assessment items and we ask you to explain why they would be useful. We also encourage you to provide reasons for your decision when you give the item a low score.

NOTE: If you cannot complete the survey in one setting, a "save and continue later" feature is available (button located at the bottom of each survey page). Once you have logged out, to return, simply click on the link received by email to return to the survey.

----Pour chaque énoncés inclus dans notre outil, nous vous demandons d'évaluer : Est-il important* compte tenu de l'objectif de mettre à l'échelle une innovation en santé pour obtenir un plus grand impact ?

1 = non pertinent

2 = impossible d'évaluer la pertinence sans révision de l'item, ou l'item a besoin d'une telle révision qu'il n'est même pas pertinent

3 = pertinent mais nécessite des modifications mineures

4 = très pertinent et succinct

Est-il clair pour tous les potentiel.le.s utilisateur.rice.s de l'outil ?

1 = pas clair

2 = l'item nécessite une certaine révision

3 = clair mais nécessite une révision mineure

4 = très clair

Est-il nécessaire* d'être inclus dans un outil dont l'objectif est d'évaluer le potentiel de mise à l'échelle des innovations en santé ?

1 = non nécessaire

2 = utile mais pas essentiel

3 = essentiel

*Les échelles d'évaluation seront disponibles à la fin de chaque session à titre de rappel.

*Veuillez noter que si un énoncé pertinent apporte une information importante, qu'il serait intéressant de considérer en ce qui concerne la mise à l'échelle, un énoncé nécessaire exprime une information indispensable pour décider sur la mise à l'échelle d'une innovation en santé.

Questions ouvertes (texte libre)

Vous trouverez des questions ouvertes (texte libre) à la fin de chaque section pour collecter vos commentaires et suggestions. Vous pouvez suggérer des autres items d'évaluation du potentiel de mise à l'échelle selon vos perceptions et nous vous demandons de justifier leur inclusion. Nous vous encourageons également à justifier vos décisions pour les scores faibles.

NOTE: Vous pourrez compléter le questionnaire à tout moment, en cliquant sur le lien dans votre courriel, qui vous redirigera automatiquement à l'endroit où vous étiez rendu. Une fois que vous vous êtes déconnecté, pour revenir, il suffit de cliquer sur le lien reçu par e-mail pour revenir à l'enquête.

J. Acceptability of innovation at scale This component comprises statements related to the acceptability of the innovation. It should be well accepted and considered appropriate among stakeholders and the target population.

J. Acceptabilité de l'innovation Cette composante rassemble des énoncés liés à l'acceptabilité de l'innovation. Elle devra être bien acceptée et considérée comme appropriée parmi les parties prenantes et la population ciblée.

*Please ensure that you have answered all questions before submitting.

*The meaning of the scales can be found at the end of the page.

*Veuillez vous assurer que vous avez répondu à toutes les questions avant de les soumettre.

*La signification des échelles se trouve en fin de page.

31. There are data on the adaptability of the innovation. 31. Il existe des données sur l'adaptabilité de l'innovation.

	1	2	3	4
Is the item important? / L'item est-il important ?	○	○	○	○
Is the item clear? / L'item est-il clair ?	○	○	○	○

	1	2	3
L'item est-il nécessaire ?	○	○	○

32. The innovation is presented appropriately using ideas and language that are meaningful to the target populations. 32. L'innovation est présentée de façon appropriée en utilisant un langage significatif pour les populations ciblées.

	1	2	3	4
Is the item important? / L'item est-il important ?	○	○	○	○
Is the item clear? / L'item est-il clair ?	○	○	○	○

	1	2	3
Is the item necessary? / L'item est-il nécessaire ?	○	○	○

Please provide any additional comments regarding the relevance, clarity and necessity of the items in this section.

Veuillez fournir tout commentaire supplémentaire concernant la pertinence, la clarté et la nécessité des items de cette section.

La version française suit.

For each scalability statement included in our tool, we are asking you to rate: Is it relevant* considering the aim to scale a health innovation for achieving greater impact?

1 = not relevant

2 = can't assess its relevance unless item is revised, or so much revision necessary that it would no longer be relevant

3 = relevant but needs minor alteration

4 = very relevant and succinct

Is it clear to all potential end users of the tool?

1 = not clear

2 = item needs some revision

3 = clear but needs minor revision

4 = very clear

Is it necessary* to be included in a tool for assessing the scalability of health innovations?

1 = not necessary

2 = useful but not essential

3 = essential

*Please note that while a relevant statement bring an important information, which would be interesting to consider regarding the scalability, a necessary statement express an information that is indispensable to decide about the scaling of a health innovation.

Open-ended (free text) questions

There are open-ended (free text) questions to collect comments and suggestions at the end of each section. You can suggest other assessment items and we ask you to explain why they would be useful. We also encourage you to provide reasons for your decision when you give the item a low score.

NOTE: If you cannot complete the survey in one setting, a "save and continue later" feature is available (button located at the bottom of each survey page). Once you have logged out, to return, simply click on the link received by email to return to the survey.

----Pour chaque énoncés inclus dans notre outil, nous vous demandons d'évaluer : Est-il important* compte tenu de l'objectif de mettre à l'échelle une innovation en santé pour obtenir un plus grand impact ?

1 = non pertinent

2 = impossible d'évaluer la pertinence sans révision de l'item, ou l'item a besoin d'une telle révision qu'il n'est même pas pertinent

3 = pertinent mais nécessite des modifications mineures

4 = très pertinent et succinct

Est-il clair pour tous les potentiel.le.s utilisateur.rice.s de l'outil ?

1 = pas clair

2 = l'item nécessite une certaine révision

3 = clair mais nécessite une révision mineure

4 = très clair

Est-il nécessaire* d'être inclus dans un outil dont l'objectif est d'évaluer le potentiel de mise à l'échelle des innovations en santé ?

1 = non nécessaire

2 = utile mais pas essentiel

3 = essentiel

*Les échelles d'évaluation seront disponibles à la fin de chaque session à titre de rappel.

*Veuillez noter que si un énoncé pertinent apporte une information importante, qu'il serait intéressant de considérer en ce qui concerne la mise à l'échelle, un énoncé nécessaire exprime une information indispensable pour décider sur la mise à l'échelle d'une innovation en santé.

Questions ouvertes (texte libre)

Vous trouverez des questions ouvertes (texte libre) à la fin de chaque section pour collecter vos commentaires et suggestions. Vous pouvez suggérer des autres items d'évaluation du potentiel de mise à l'échelle selon vos perceptions et nous vous demandons de justifier leur inclusion. Nous vous encourageons également à justifier vos décisions pour les scores faibles.

NOTE: Vous pourrez compléter le questionnaire à tout moment, en cliquant sur le lien dans votre courriel, qui vous redirigera automatiquement à l'endroit où vous étiez rendu. Une fois que vous vous êtes déconnecté, pour revenir, il suffit de cliquer sur le lien reçu par e-mail pour revenir à l'enquête.

K. Adoption of innovation at scale This component comprises statements related to the adoption of the innovation. It is important to consider how many ordinary people, professionals, or service units will use or even integrate the innovation as a practice. The numerator and denominator, respectively, reflect the number of units (people, services, etc.) that have adopted the innovation and the total number targeted since its conception.

K. Adoption de l'innovation à grande échelle Cette composante rassemble des énoncés liés à l'adoption de l'innovation. Il est important de considérer combien de personnes, de professionnels ou d'unités de service utiliseront ou même intégreront l'innovation en tant que pratique. Le numérateur et le dénominateur, respectivement, reflètent le nombre d'unités (personnes, services, etc.) qui ont adopté l'innovation et le nombre total ciblé depuis sa conception.

*Please ensure that you have answered all questions before submitting.

*The meaning of the scales can be found at the end of the page.

*Veuillez vous assurer que vous avez répondu à toutes les questions avant de les soumettre.

*La signification des échelles se trouve en fin de page.

33. There are data on the adoption of the innovation among the target population (numerator & denominator). 33. Il existe des données sur l'adoption de l'innovation parmi les populations ciblées (numérateur et dénominateur).

	1	2	3	4
Is the item important? / L'item est-il important ?	○	○	○	○
Is the item clear? / L'item est-il clair ?	○	○	○	○

	1	2	3
Is the item necessary? / L'item est-il nécessaire ?	○	○	○

34. There are data on target population intention to adopt the innovation. 34. Il existe des données sur l'intention des populations ciblées d'adopter l'innovation.

	1	2	3	4
Is the item important? / L'item est-il important ?	○	○	○	○
Is the item clear? / L'item est-il clair ?	○	○	○	○

	1	2	3
Is the item necessary? / L'item est-il nécessaire ?	○	○	○

Please provide any additional comments regarding the relevance, clarity and necessity of the items in this section.

Veuillez fournir tout commentaire supplémentaire concernant la pertinence, la clarté et la nécessité des items de cette section.

La version française suit.

For each scalability statement included in our tool, we are asking you to rate: Is it relevant* considering the aim to scale a health innovation for achieving greater impact?

1 = not relevant

2 = can't assess its relevance unless item is revised, or so much revision necessary that it would no longer be relevant

3 = relevant but needs minor alteration

4 = very relevant and succinct

Is it clear to all potential end users of the tool?

1 = not clear

2 = item needs some revision

3 = clear but needs minor revision

4 = very clear

Is it necessary* to be included in a tool for assessing the scalability of health innovations?

1 = not necessary

2 = useful but not essential

3 = essential

*Please note that while a relevant statement bring an important information, which would be interesting to consider regarding the scalability, a necessary statement express an information that is indispensable to decide about the scaling of a health innovation.

Open-ended (free text) questions

There are open-ended (free text) questions to collect comments and suggestions at the end of each section. You can suggest other assessment items and we ask you to explain why they would be useful. We also encourage you to provide reasons for your decision when you give the item a low score.

NOTE: If you cannot complete the survey in one setting, a "save and continue later" feature is available (button located at the bottom of each survey page). Once you have logged out, to return, simply click on the link received by email to return to the survey.

----Pour chaque énoncés inclus dans notre outil, nous vous demandons d'évaluer : Est-il important* compte tenu de l'objectif de mettre à l'échelle une innovation en santé pour obtenir un plus grand impact ?

1 = non pertinent

2 = impossible d'évaluer la pertinence sans révision de l'item, ou l'item a besoin d'une telle révision qu'il n'est même pas pertinent

3 = pertinent mais nécessite des modifications mineures

4 = très pertinent et succinct

Est-il clair pour tous les potentiel.le.s utilisateur.rice.s de l'outil ?

1 = pas clair

2 = l'item nécessite une certaine révision

3 = clair mais nécessite une révision mineure

4 = très clair

Est-il nécessaire* d'être inclus dans un outil dont l'objectif est d'évaluer le potentiel de mise à l'échelle des innovations en santé ?

1 = non nécessaire

2 = utile mais pas essentiel

3 = essentiel

*Les échelles d'évaluation seront disponibles à la fin de chaque session à titre de rappel.

*Veuillez noter que si un énoncé pertinent apporte une information importante, qu'il serait intéressant de considérer en ce qui concerne la mise à l'échelle, un énoncé nécessaire exprime une information indispensable pour décider sur la mise à l'échelle d'une innovation en santé.

Questions ouvertes (texte libre)

Vous trouverez des questions ouvertes (texte libre) à la fin de chaque section pour collecter vos commentaires et suggestions. Vous pouvez suggérer des autres items d'évaluation du potentiel de mise à l'échelle selon vos perceptions et nous vous demandons de justifier leur inclusion. Nous vous encourageons également à justifier vos décisions pour les scores faibles.

NOTE: Vous pourrez compléter le questionnaire à tout moment, en cliquant sur le lien dans votre courriel, qui vous redirigera automatiquement à l'endroit où vous étiez rendu. Une fois que vous vous êtes déconnecté, pour revenir, il suffit de cliquer sur le lien reçu par e-mail pour revenir à l'enquête.

L. Scaling environment This component comprises statements related to the setting in which the innovation will be scaled. It is necessary to consider whether the context in which the innovation will be disseminated is sufficiently similar or compatible with the pilot context from which evidence of the innovation's impact was gathered. In addition, key stakeholders must be present locally for scaling.

L. Milieu de mise à l'échelle Cette composante rassemble des énoncés liés au milieu de mise à l'échelle de l'innovation. Il est nécessaire d'examiner si le contexte dans lequel l'innovation sera diffusée est suffisamment similaire ou compatible avec le contexte pilote à partir duquel les preuves de l'impact de l'innovation ont été recueillies. En outre, il est nécessaire que les principaux acteurs soient présents localement pour la mise à l'échelle.

*Please ensure that you have answered all questions before submitting.

*The meaning of the scales can be found at the end of the page.

*Veuillez vous assurer que vous avez répondu à toutes les questions avant de les soumettre.

*La signification des échelles se trouve en fin de page.

35. The innovation has been tested in the type of environment in which it is to be scaled up. 35. L'innovation a été testée dans le même type de milieu où elle sera mise à l'échelle.

	1	2	3	4
Is the item important? / L'item est-il important ?	○	○	○	○
Is the item clear? / L'item est-il clair ?	○	○	○	○

	1	2	3
Is the item necessary? / L'item est-il nécessaire ?	○	○	○

36. Local multi-stakeholder partnerships have been established to support the scaling. 36. Les partenariats multipartites locaux sont en place pour soutenir la mise à l'échelle.

	1	2	3	4
Is the item important? / L'item est-il important ?	○	○	○	○
Is the item clear? / L'item est-il clair ?	○	○	○	○

	1	2	3
Is the item necessary? / L'item est-il nécessaire ?	○	○	○

37. Appropriately trained personnel exists and is available for scaling [in target sites]. 37. Il existe une main-d'œuvre formée de façon appropriée et elle est disponible dans le milieu où l'innovation sera mise à l'échelle.

	1	2	3	4
Is the item important? / L'item est-il important ?	○	○	○	○
Is the item clear? / L'item est-il clair ?	○	○	○	○

	1	2	3
Is the item necessary? / L'item est-il nécessaire ?	○	○	○

Please provide any additional comments regarding the relevance, clarity and necessity of the items in this section.

Veuillez fournir tout commentaire supplémentaire concernant la pertinence, la clarté et la nécessité des items de cette section.

La version française suit.

For each scalability statement included in our tool, we are asking you to rate: Is it relevant* considering the aim to scale a health innovation for achieving greater impact?

1 = not relevant

2 = can't assess its relevance unless item is revised, or so much revision necessary that it would no longer be relevant

3 = relevant but needs minor alteration

4 = very relevant and succinct

Is it clear to all potential end users of the tool?

1 = not clear

2 = item needs some revision

3 = clear but needs minor revision

4 = very clear

Is it necessary* to be included in a tool for assessing the scalability of health innovations?

1 = not necessary

2 = useful but not essential

3 = essential

*Please note that while a relevant statement bring an important information, which would be interesting to consider regarding the scalability, a necessary statement express an information that is indispensable to decide about the scaling of a health innovation.

Open-ended (free text) questions

There are open-ended (free text) questions to collect comments and suggestions at the end of each section. You can suggest other assessment items and we ask you to explain why they would be useful. We also encourage you to provide reasons for your decision when you give the item a low score.

NOTE: If you cannot complete the survey in one setting, a "save and continue later" feature is available (button located at the bottom of each survey page). Once you have logged out, to return, simply click on the link received by email to return to the survey.

----Pour chaque énoncés inclus dans notre outil, nous vous demandons d'évaluer : Est-il important* compte tenu de l'objectif de mettre à l'échelle une innovation en santé pour obtenir un plus grand impact ?

1 = non pertinent

2 = impossible d'évaluer la pertinence sans révision de l'item, ou l'item a besoin d'une telle révision qu'il n'est même pas pertinent

3 = pertinent mais nécessite des modifications mineures

4 = très pertinent et succinct

Est-il clair pour tous les potentiel.le.s utilisateur.rice.s de l'outil ?

1 = pas clair

2 = l'item nécessite une certaine révision

3 = clair mais nécessite une révision mineure

4 = très clair

Est-il nécessaire* d'être inclus dans un outil dont l'objectif est d'évaluer le potentiel de mise à l'échelle des innovations en santé ?

1 = non nécessaire

2 = utile mais pas essentiel

3 = essentiel

*Les échelles d'évaluation seront disponibles à la fin de chaque session à titre de rappel.

*Veuillez noter que si un énoncé pertinent apporte une information importante, qu'il serait intéressant de considérer en ce qui concerne la mise à l'échelle, un énoncé nécessaire exprime une information indispensable pour décider sur la mise à l'échelle d'une innovation en santé.

Questions ouvertes (texte libre)

Vous trouverez des questions ouvertes (texte libre) à la fin de chaque section pour collecter vos commentaires et suggestions. Vous pouvez suggérer des autres items d'évaluation du potentiel de mise à l'échelle selon vos perceptions et nous vous demandons de justifier leur inclusion. Nous vous encourageons également à justifier vos décisions pour les scores faibles.

NOTE: Vous pourrez compléter le questionnaire à tout moment, en cliquant sur le lien dans votre courriel, qui vous redirigera automatiquement à l'endroit où vous étiez rendu. Une fois que vous vous êtes déconnecté, pour revenir, il suffit de cliquer sur le lien reçu par e-mail pour revenir à l'enquête.

M. Infrastructure required for scaling This component comprises statements related to the infrastructure required for innovation scaling. Evidence must be gathered concerning the feasibility of the innovation in relation to the infrastructure needed for its realisation. For scaling to be successful, it is important for the organisational infrastructure to be available throughout the process, including monitoring and assessment structures (professionals, processes, measures, and the like) and a skilled workforce.

M. Infrastructure requise pour la mise à l'échelle Cette composante rassemble des énoncés liés à l'infrastructure requise pour la mise à l'échelle de l'innovation. Il est nécessaire de rassembler des preuves de la faisabilité de l'innovation par rapport à l'infrastructure nécessaire à sa réalisation. Pour une mise à l'échelle réussie, il est important que l'infrastructure organisationnelle soit disponible tout au long du processus, y compris la main-d'œuvre qualifiée et les structures de suivi et d'évaluation (professionnels, processus, mesures, etc.).

*Please ensure that you have answered all questions before submitting.

*The meaning of the scales can be found at the end of the page.

*Veuillez vous assurer que vous avez répondu à toutes les questions avant de les soumettre.

*La signification des échelles se trouve en fin de page.

38. There are data on the feasibility of the innovation. 38. Il existe des données sur la faisabilité de l'innovation.

	1	2	3	4
Is the item important? / L'item est-il important ?	○	○	○	○
Is the item clear? / L'item est-il clair ?	○	○	○	○

	1	2	3
Is the item necessary? / L'item est-il nécessaire ?	○	○	○

39. Infrastructure requirements for scaling the innovation are achievable. 39. Les exigences d'infrastructure pour la mise à l'échelle de l'innovation sont réalisables.

	1	2	3	4
Is the item important? / L'item est-il important ?	○	○	○	○

Is the item clear? / L'item est-il clair ?	○	○	○	○

	1	2	3
Is the item necessary? / L'item est-il nécessaire ?	○	○	○

40. The organizational infrastructure required is available for scaling the innovation. 40. L'infrastructure organisationnelle requise est disponible pour la mise à l'échelle de l'innovation.

	1	2	3	4
Is the item important? / L'item est-il important ?	○	○	○	○
Is the item clear? / L'item est-il clair ?	○	○	○	○

	1	2	3
Is the item necessary? / L'item est-il nécessaire ?	○	○	○

41. Structures are in place for monitoring the scaling process. 41. Des structures sont en place pour surveiller le processus de mise à l'échelle.

	1	2	3	4
Is the item important? / L'item est-il important ?	○	○	○	○
Is the item clear? / L'item est-il clair ?	○	○	○	○

	1	2	3
Is the item necessary? / L'item est-il nécessaire ?	○	○	○

42. Structures are in place for evaluating the scaling process. 42. Des structures sont en place pour évaluer le processus de mise à l'échelle.

	1	2	3	4
Is the item important? / L'item est-il important ?	○	○	○	○
Is the item clear? / L'item est-il clair ?	○	○	○	○

	1	2	3
Is the item necessary? / L'item est-il nécessaire ?	○	○	○

Please provide any additional comments regarding the relevance, clarity and necessity of the items in this section.

Veuillez fournir tout commentaire supplémentaire concernant la pertinence, la clarté et la nécessité des items de cette section.

La version française suit.

For each scalability statement included in our tool, we are asking you to rate: Is it relevant* considering the aim to scale a health innovation for achieving greater impact?

1 = not relevant

2 = can't assess its relevance unless item is revised, or so much revision necessary that it would no longer be relevant

3 = relevant but needs minor alteration

4 = very relevant and succinct

Is it clear to all potential end users of the tool?

1 = not clear

2 = item needs some revision

3 = clear but needs minor revision

4 = very clear

Is it necessary* to be included in a tool for assessing the scalability of health innovations?

1 = not necessary

2 = useful but not essential

3 = essential

*Please note that while a relevant statement bring an important information, which would be interesting to consider regarding the scalability, a necessary statement express an information that is indispensable to decide about the scaling of a health innovation.

Open-ended (free text) questions

There are open-ended (free text) questions to collect comments and suggestions at the end of each section. You can suggest other assessment items and we ask you to explain why they would be useful. We also encourage you to provide reasons for your decision when you give the item a low score.

NOTE: If you cannot complete the survey in one setting, a "save and continue later" feature is available (button located at the bottom of each survey page). Once you have logged out, to return, simply click on the link received by email to return to the survey.

----Pour chaque énoncés inclus dans notre outil, nous vous demandons d'évaluer : Est-il important* compte tenu de l'objectif de mettre à l'échelle une innovation en santé pour obtenir un plus grand impact ?

1 = non pertinent

2 = impossible d'évaluer la pertinence sans révision de l'item, ou l'item a besoin d'une telle révision qu'il n'est même pas pertinent

3 = pertinent mais nécessite des modifications mineures

4 = très pertinent et succinct

Est-il clair pour tous les potentiel.le.s utilisateur.rice.s de l'outil ?

1 = pas clair

2 = l'item nécessite une certaine révision

3 = clair mais nécessite une révision mineure

4 = très clair

Est-il nécessaire* d'être inclus dans un outil dont l'objectif est d'évaluer le potentiel de mise à l'échelle des innovations en santé ?

1 = non nécessaire

2 = utile mais pas essentiel

3 = essentiel

*Les échelles d'évaluation seront disponibles à la fin de chaque session à titre de rappel.

*Veuillez noter que si un énoncé pertinent apporte une information importante, qu'il serait intéressant de considérer en ce qui concerne la mise à l'échelle, un énoncé nécessaire exprime une information indispensable pour décider sur la mise à l'échelle d'une innovation en santé.

Questions ouvertes (texte libre)

Vous trouverez des questions ouvertes (texte libre) à la fin de chaque section pour collecter vos commentaires et suggestions. Vous pouvez suggérer des autres items d'évaluation du potentiel de mise à l'échelle selon vos perceptions et nous vous demandons de justifier leur inclusion. Nous vous encourageons également à justifier vos décisions pour les scores faibles.

NOTE: Vous pourrez compléter le questionnaire à tout moment, en cliquant sur le lien dans votre courriel, qui vous redirigera automatiquement à l'endroit où vous étiez rendu. Une fois que vous vous êtes déconnecté, pour revenir, il suffit de cliquer sur le lien reçu par e-mail pour revenir à l'enquête.

N. Sustainability This component comprises statements related to the sustainability of the innovation and involves considering the availability of human and material resources over time and how long they will be available.

N. Pérennisation Cette composante rassemble des énoncés liés à la pérennisation de l'innovation. Cela implique de réfléchir à la disponibilité des ressources humaines et matérielles dans le temps et à la durée pendant lesquelles elles seront disponibles.

*Please ensure that you have answered all questions before submitting.

*The meaning of the scales can be found at the end of the page.

*Veuillez vous assurer que vous avez répondu à toutes les questions avant de les soumettre.

*La signification des échelles se trouve en fin de page.

43. The sustainability (maintaining the scaling on a lasting basis) has been considered. 43. La pérennisation de la mise à l'échelle a été considérée.

	1	2	3	4
Is the item important? / L'item est-il important ?	○	○	○	○
Is the item clear? / L'item est-il clair ?	○	○	○	○

	1	2	3
Is the item necessary? / L'item est-il nécessaire ?	○	○	○

44. The human and financial resources required for scaling are sustainable. 44. Les ressources humaines et financières requises pour la mise à l'échelle de l'innovation sont pérennes.

	1	2	3	4
Is the item important? / L'item est-il important ?	○	○	○	○
Is the item clear? / L'item est-il clair ?	○	○	○	○

	1	2	3
Is the item necessary? / L'item est-il nécessaire ?	○	○	○

45. There are data on how long the innovation can it be sustained at scale. 45. Il existe des données sur la durée de la pérennité de l'innovation à grande échelle.

	1	2	3	4
Is the item important? / L'item est-il important ?	◯	◯	◯	◯
Is the item clear? / L'item est-il clair ?	◯	◯	◯	◯

	1	2	3
Is the item necessary? / L'item est-il nécessaire ?	◯	◯	◯

Please provide any additional comments regarding the relevance, clarity and necessity of the items in this section.

Veuillez fournir tout commentaire supplémentaire concernant la pertinence, la clarté et la nécessité des items de cette section.

La version française suit.

For each scalability statement included in our tool, we are asking you to rate: Is it relevant* considering the aim to scale a health innovation for achieving greater impact?

1 = not relevant

2 = can't assess its relevance unless item is revised, or so much revision necessary that it would no longer be relevant

3 = relevant but needs minor alteration

4 = very relevant and succinct

Is it clear to all potential end users of the tool?

1 = not clear

2 = item needs some revision

3 = clear but needs minor revision

4 = very clear

Is it necessary* to be included in a tool for assessing the scalability of health innovations?

1 = not necessary

2 = useful but not essential

3 = essential

*Please note that while a relevant statement bring an important information, which would be interesting to consider regarding the scalability, a necessary statement express an information that is indispensable to decide about the scaling of a health innovation.

Open-ended (free text) questions

There are open-ended (free text) questions to collect comments and suggestions at the end of each section. You can suggest other assessment items and we ask you to explain why they would be useful. We also encourage you to provide reasons for your decision when you give the item a low score.

NOTE: If you cannot complete the survey in one setting, a "save and continue later" feature is available (button located at the bottom of each survey page). Once you have logged out, to return, simply click on the link received by email to return to the survey.

----Pour chaque énoncés inclus dans notre outil, nous vous demandons d'évaluer : Est-il important* compte tenu de l'objectif de mettre à l'échelle une innovation en santé pour obtenir un plus grand impact ?

1 = non pertinent

2 = impossible d'évaluer la pertinence sans révision de l'item, ou l'item a besoin d'une telle révision qu'il n'est même pas pertinent

3 = pertinent mais nécessite des modifications mineures

4 = très pertinent et succinct

Est-il clair pour tous les potentiel.le.s utilisateur.rice.s de l'outil ?1 = pas clair

2 = l'item nécessite une certaine révision

3 = clair mais nécessite une révision mineure

4 = très clair

Est-il nécessaire* d'être inclus dans un outil dont l'objectif est d'évaluer le potentiel de mise à l'échelle des innovations en santé ?

1 = non nécessaire

2 = utile mais pas essentiel

3 = essentiel

*Les échelles d'évaluation seront disponibles à la fin de chaque session à titre de rappel.

*Veuillez noter que si un énoncé pertinent apporte une information importante, qu'il serait intéressant de considérer en ce qui concerne la mise à l'échelle, un énoncé nécessaire exprime une information indispensable pour décider sur la mise à l'échelle d'une innovation en santé.

Questions ouvertes (texte libre)

Vous trouverez des questions ouvertes (texte libre) à la fin de chaque section pour collecter vos commentaires et suggestions. Vous pouvez suggérer des autres items d'évaluation du potentiel de mise à l'échelle selon vos perceptions et nous vous demandons de justifier leur inclusion. Nous vous encourageons également à justifier vos décisions pour les scores faibles.

NOTE: Vous pourrez compléter le questionnaire à tout moment, en cliquant sur le lien dans votre courriel, qui vous redirigera automatiquement à l'endroit où vous étiez rendu. Une fois que vous vous êtes déconnecté, pour revenir, il suffit de cliquer sur le lien reçu par e-mail pour revenir à l'enquête.
